# Expansion of signaling genes for adaptive immune system evolution in early vertebrates

**DOI:** 10.1186/1471-2164-9-218

**Published:** 2008-05-14

**Authors:** Kinya Okada, Kiyoshi Asai

**Affiliations:** 1 Department of Computational Biology, Graduate School of Frontier Sciences, The University of Tokyo, Kashiwa, Chiba, Japan; 2 Computational Biology Research Center, National Institute of Advanced Industrial Science and Technology (AIST), Koto-ku, Tokyo, Japan

## Abstract

**Background:**

The adaptive immune system (AIS) of jawed vertebrates is a sophisticated system mediated by numerous genes in specialized cells. Phylogenetic analysis indicates that emergence of the AIS followed the occurrence of two rounds of whole-genome duplication (2R-WGD) in early vertebrates, but little direct evidence linking these two events is available.

**Results:**

We examined the relationship between 2R-WGD and the gain of AIS-related functions by numerous genes. To analyze the evolution of the many genes related to signal transduction in the AIS (defined as AIS genes), we identified groups of genes (defined as AIS subfamilies) that included at least one human AIS gene, its paralogs (if any), and its *Drosophila *ortholog(s). Genomic mapping revealed that numerous pairs of AIS genes and their paralogs were part of paralogons – series of paralogous regions that derive from a common ancestor – throughout the human genome, indicating that the genes were retained as duplicates after 2R-WGD. Outgroup comparison analysis revealed that subfamilies in which human and fly genes shared a nervous system-related function were significantly enriched among AIS subfamilies, as compared with the overall incidence of shared nervous system-related functions among all subfamilies in bilaterians. This finding statistically supports the hypothesis that AIS-related signaling genes were ancestrally involved in the nervous system of urbilaterians.

**Conclusion:**

The current results suggest that 2R-WGD played a major role in the duplication of many signaling genes, ancestrally used in nervous system development and function, that were later co-opted for new functions during evolution of the AIS.

## Background

The AIS in jawed vertebrates is a sophisticated system mediated by numerous genes in specialized cells [1]. Because the AIS is conserved widely among jawed vertebrates but not invertebrates [2], it is widely held that the AIS appeared suddenly, as an immunologic "big bang," in the common ancestor of jawed vertebrates [3]. The recently sequenced genome of *Stringylocentrotus purpuratus *(purple sea urchin)[4], one of the closest relatives of chordates, has revealed that although they lack an AIS, sea urchins have almost all of the vertebrate gene repertoire, including many genes involved in the AIS [4,5]. This situation indicates that many of the genes involved in the AIS arose long before the emergence of jawed vertebrates (or at least that of the common ancestor of jawed vertebrates and sea urchins) and thereafter evolved concurrently to gain new functions in the AIS at the immunologic big bang.

Gene duplications are important evolutionary paths for the acquisition of new functions, because one copy of a duplicated gene can accumulate mutations and acquire novel functions while the other retains the original function. Such duplications can involve individual genes, genomic segments, or whole genomes. Because they generate a large number of gene duplicates concurrently, whole-genome duplications (WGDs) are considered particularly important evolutionary events [6]. It has long been hypothesized that a WGD occurred twice in early vertebrates (two rounds of whole-genome duplication, 2R-WGD)[6]. This hypothesis has recently been supported by clear patterns of four-way paralogous regions occurring throughout the human genome [7,8].

Phylogenetic analysis indicates that the occurrence of 2R-WGD preceded emergence of the AIS [2]; therefore, 2R-WGD may have been important in the acquisition of AIS-related functions by numerous genes at immunologic big bang. However, 2R-WGD has been directly linked to limited AIS-related genes that are located in only a few paralogons in the human genome [2,9,10], indicating that the genes arose from 2R-WGD. These currently available examples are too limited to reveal the precise relationship between 2R-WGD and the acquisition of AIS-related functions by the numerous genes involved in this system.

We focus here on many signaling genes in the AIS and investigate the extent to which 2R-WGD contributed to their gains of function in the AIS. In addition, to provide insight into the ancestral status of the AIS-related genes before 2R-WGD, we compared these genes with their orthologs in an invertebrate (*Drosophila*) that lacks an AIS.

## Results

### Molecular functions of paralogs in paralogons formed in early vertebrates

By using an anchoring strategy combined with a phylogenetic approach [8], we identified in the human genome 357 paralogons including 2842 BV paralogous genes (formed at the Base of the Vertebrate lineage). An important first step in demonstrating that 2R-WGD indeed was important in the evolution of genes involved in the AIS would be to show the types of genes that were retained after duplication by 2R-WGD. To this end, we used Gene Ontology (GO) annotations to investigate the molecular roles of the BV paralogous genes. GO terms such as "regulation of biological process" (GO term ID:0050789; *P *= 4.05 × 10^-14^) and "signal transduction" (GO term ID:0007165; *P *= 9.52 × 10^-24^) were over-represented among the BV paralogous genes (Table 1), as indicated in previous WGD studies [11-15]. We therefore focused on genes in the AIS with molecular roles related to the over-represented GO terms and examined the relationship between the evolution of these genes and 2R-WGD.

**Table 1:** Top five Gene Ontology (GO) slim terms of BV paralogous genes

GO ID	GO term	Observed number of BV paralogous genes (%)	*P *value*
GO:0050789	regulation of biological process	698 (25% = 698/2842)	4.05 × 10^-14^
GO:0007165	signal transduction	634 (22% = 634/2842)	9.52 × 10^-24^
GO:0007275	multicellular organismal development	397 (14% = 397/2842)	1.59 × 10^-24^
GO:0006350	transcription	388 (14% = 388/2842)	4.49 × 10^-10^
GO:0006464	protein modification process	326 (11% = 326/2842)	1.65 × 10^-27^

* *P *values are calculated by using hypergeometric distribution.

A BV paralogous gene is a paralogous gene that was formed at the Base of the Vertebrate lineage.

### Detection of AIS subfamilies

In the AIS, signal transduction machineries play indispensable roles as mediators between the diverse extracellular stimuli received by membrane-bound receptors and various induced biological processes. We focused on an antigen-recognition receptor pathway, chemokine receptor pathway, and cytokine receptor pathway as representatives of the various signal transduction machineries in the AIS. We prepared a list of the 61 families of human genes (defined as AIS families) that comprise these three signaling pathways (see Additional File 1) and that include many genes essential to the AIS [1].

If the genes in an AIS family have emerged at or before the divergence of protostomes and deuterostomes, then the AIS family will encompass one or more subfamilies, each of which will include all the descendants of a single ancestral gene in the common ancestor of protostomes and deuterostomes (i.e., the ancestral bilaterian). We comprehensively identified such subfamilies by applying a clustering algorithm to the complete gene sets of one invertebrate (*Drosophila*) and three vertebrates (human, mouse, and medaka) and investigated whether any of the AIS families contained one or more resulting subfamilies. Consequently, 93% (57 of 61) of the AIS families encompassed at least one subfamily (see Additional File 1), indicating that their ancestral genes existed in the ancestral bilaterian.

However, in the case of AIS families with multiple subfamilies, one or more subfamilies may not contain any AIS-related genes, because each AIS family can include genes that are not involved in the AIS. By using published information on the AIS-related functions of each gene in the subfamilies of all AIS families, we identified 50 subfamilies that each included at least one member gene involved in the AIS (defined as an AIS gene); the 50 subfamilies were defined as "AIS subfamilies" (see Additional Files 2, 3). Because each subfamily included all the descendants of a single ancestral gene in the common ancestor of protostomes (i.e., *Drosophila*) and deuterostomes (i.e., human, mouse, and medaka), an AIS subfamily included at least one human AIS gene, its paralog(s) (if any), and its *Drosophila *ortholog(s).

### Timing of gene duplications in AIS subfamilies

We found that 84% (42 of 50) of the AIS subfamilies included multiple human genes (Additional Files 2, 3), indicating that the numbers of subfamily members increased through gene duplication after the divergence of protostomes and deuterostomes. To clarify the timing of the duplications, we conducted phylogenetic analyses of the AIS subfamilies. Examination of the resulting phylogenetic trees showed that 88% of the total duplications (65 of 74) in the AIS subfamilies occurred in the early vertebrate lineage (i.e., before the divergence of fish and tetrapods; see Additional Files 4, 5).

### Genomic mapping analysis

**Figure 1:**
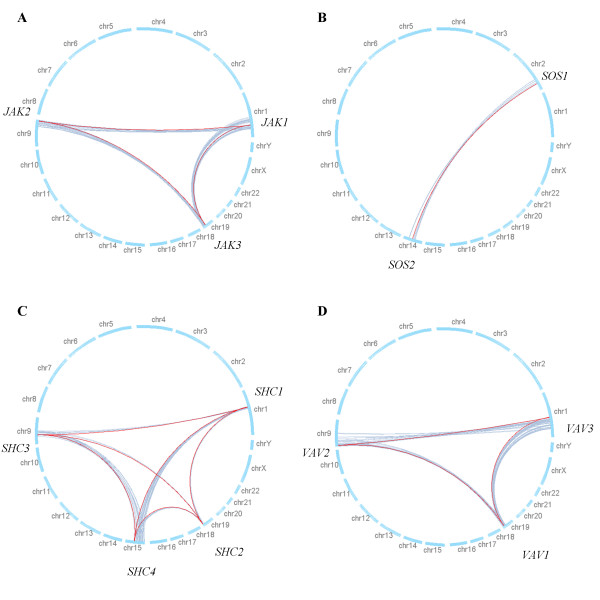
**Genomic maps of AIS subfamilies**. The chromosomes of the human genome from chromosome 1 (chr1) to chromosomes X, Y (chrX, Y) are arranged in a circle (blue line). Red lines connect the genomic map positions of the members in each subfamily: (A) JAK, Janus kinase; (B) SOS, son of sevenless homolog; (C) SHC, SHC (Src homology 2 domain containing)-transforming protein; and (D) VAV, *vav *oncogene. Gray lines connect the genomic map positions of the genes in each BV paralogous pair (a paralogous pair that were formed at the Base of the Vertebrate lineage) of paralogons. Additional File 7 contains the genomic maps of all AIS subfamilies.

To determine whether gene duplications in the AIS subfamilies occurred as a result of 2R-WGD, we conducted a genomic mapping analysis, in which we considered that if two genes of a given paralogous pair were located in the separate two regions of a paralogon, the genes were retained as duplicates from 2R-WGD. For example, three members of the JAK (Janus kinase) subfamily – *JAK1*, *JAK2*, and *JAK3 *– are located on human chromosomes 1, 9, and 19, respectively. The analysis revealed that the pairs of these members were part of paralogons (see Additional File 6), indicating that these genes were retained as duplicates from 2R-WGD. To visualize the relationships of the members' locations and paralogons, we used red lines to connect the genomic map positions of the members, whereas we used gray lines to connect the genes in each BV paralogous pair of paralogons in the human genome. The red lines lay within the groups of gray lines (Fig. 1A), indicating that all of the paralog pairs in the JAK subfamily were part of paralogons. A total of 67% (90 of 134) of the paralog pairs in all AIS subfamilies were part of paralogons (see Additional Files 6, 7), and this rate is significantly higher than that expected due to chance (*P *= 1.63 × 10^-50^, assuming binomial distribution under the null hypothesis that paralogs are located randomly throughout the human genome).

### Use of large-scale microarray analysis to detect tissues and organs in which the genes in AIS subfamilies are expressed at high levels

**Figure 2:**
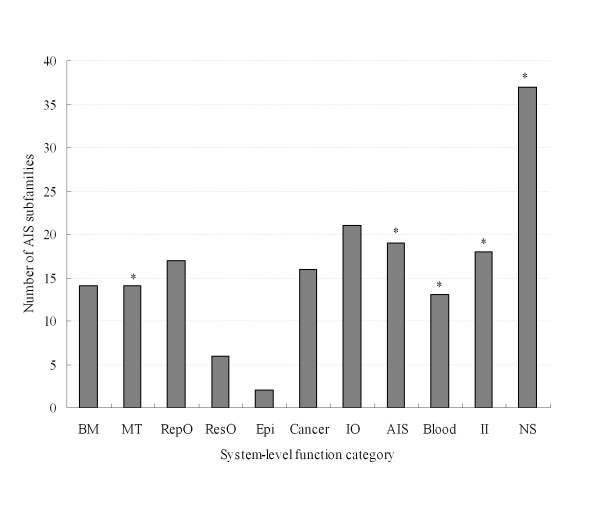
**Histogram showing the number of AIS subfamilies with each system-level function category**. Each bar shows the number of AIS subfamilies that have each system-level function category. Asterisks indicate statistically significant enrichment of categories (*P *< 0.05, calculated using hypergeometric distribution with Bonferroni correction). BM, bone marrow; MT, muscle tissue; RepO, reproductive organ; ResO, respiration organ; Epi, epithelium; IO, internal organ and metabolism system; AIS, adaptive immune system; II, innate immunity; NS, nervous system.

In the widely accepted "gene duplication and divergence" model [6], after gene duplication, a new function will be acquired by one of the duplicates as a result of accumulated mutations while the original function is retained by the other. Because AIS-related functions were acquired after 2R-WGD, ancestral non-AIS-related functions might be retained by the human genes in AIS subfamilies. To investigate the functional diversity among the human genes in the AIS subfamilies, we used large-scale microarray expression profiles of various human organs and tissues [16]. For a broad overview of tissue- and organ-specific expression, we used a classification scheme (see Additional File 8) that is a simplified version of Cell Catalogue [17] to classify all 79 tissues and organs used in the microarray data into 11 system-level function categories; we then assigned these categories to the human genes in the AIS subfamilies: if a gene was specifically expressed in at least one of the tissues or organs in a system-level function category, then the system-level function category was assigned to the gene (see Additional File 9). We then assigned a system-level function category to an AIS subfamily if the AIS subfamily included at least one member gene having the system-level function category. Consequently we found that the categories "nervous system" (NS), "innate immunity", "muscle tissue", AIS, and "blood" were significantly enriched (*P *< 0.05; calculated by using hypergeometric distribution with Bonferroni correction) among AIS subfamilies as compared with the overall distribution of these categories among all subfamilies (Fig. 2 and see Additional File 9) detected among the complete gene sets of three vertebrates (human, mouse, and medaka) and one invertebrate (*Drosophila*). This result indicates that the human genes in these AIS subfamilies have not only AIS-related functions but also other functions, particularly those related to the NS, innate immunity, muscle tissue, and blood, all of which can be considered as candidates of ancestral functions of AIS subfamilies.

### Outgroup comparison for inferring the ancestral functions of genes in AIS subfamilies

We assigned the categories NS, innate immunity, muscle tissue, and blood to the *Drosophila *genes in the AIS subfamilies according to their FlyBase annotations [18] (see Additional File 10). To infer the ancestral categories of the AIS subfamilies, we used the outgroup comparison method [19], which assumes that the categories shared between the ingroup and outgroup are relatively primitive with respect to those found only in the ingroup. That is, by comparing assigned categories of human (i.e., ingroup) and *Drosophila *(i.e., outgroup) genes in an AIS subfamily, we assigned the shared categories across these two species as ancestral functions of the subfamily. Consequently we found that at least one category was assigned to each of 71% (34 of 48; two subfamilies were not considered because their human genes lacked probes for this analysis) of the AIS subfamilies; particularly, the NS category was assigned to most (58%, 28 of 48) of the AIS subfamilies (Table 2). Furthermore, we found that the NS category was significantly over-represented among the AIS subfamilies as compared with the overall distribution of this category among all subfamilies (*P *= 9.26 × 10^-15^; calculated by using hypergeometric distribution); this over-representation of the NS category was more significant than that of any other category (Table 2). These results suggest that an ancestral function of the AIS subfamilies was associated with the NS.

**Table 2:** System-level function categories that are shared between *Drosophila *and human genes in AIS subfamilies

System-level function category	Number of AIS subfamilies (%)	*P *value*
muscle tissue	2 (4% = 2/48)	4.26 × 10^-2^
Blood	7 (15% = 7/48)	2.17 × 10^-10^
innate immunity	6 (13% = 6/48)	1.11 × 10^-9^
nervous system	28 (58% = 28/48)	9.26 × 10^-15^

AIS, adaptive immune system.

*P values are calculated by using hypergeometric distribution.

### Gene usage of AIS subfamilies in the NS and AIS

Large-scale microarray data [16] enabled us to examine the statistical significance of the enrichment of the system-level function categories among the AIS subfamilies and revealed that the most significantly enriched category among the AIS subfamilies was NS. To precisely assess gene usage in the NS and AIS among the AIS subfamilies, we listed the NS- and AIS-related functions of the human genes in the AIS subfamilies according to data in the published literature (see Additional File 11). Most of the human genes in the AIS families (43%, 56 of 129) were used in either the NS or AIS, suggesting that the functions of these genes are specialized for the respective system (see Additional File 11). In contrast, 44 genes (34%, 44 of 129) were used in both the NS and AIS (see Additional File 11), although whether the genes have different or similar functions in each system is unclear.

## Discussion

Because most traces of 2R-WGD have been deleted by subsequent gene loss and genomic rearrangement [8,20], direct evidence linking 2R-WGD and expansion of AIS subfamilies is difficult to obtain by analyzing the membership of AIS subfamilies. A 4:1 ratio in the number of vertebrate to invertebrate genes in a family (e.g., Hox clusters [21]) is considered to reflect an effect of 2R-WGD. However, because of massive gene loss after 2R-WGD, few AIS subfamilies include four human genes (see Additional File 3). Likewise, the phylogenetic timing of duplications in AIS subfamilies is inconclusive, because duplications can occur on every branch. Although the number of duplications was increased somewhat in the early vertebrate lineage (Additional File 5 and as found previously [22]), this increase may simply indicate that this evolutionary period was associated with accelerated small-scale gene duplication or a reduced rate of gene loss, rather than WGD. However, the genomic mapping analysis, which examined the relationship between the genomic map positions of members in AIS subfamilies and those of BV paralogous genes in paralogons, yielded conclusive evidence of the role of 2R-WGD in the expansion of the AIS subfamilies. This analysis revealed that most of the paralogous pairs in AIS subfamilies were part of paralogons in the human genome (Fig. 1 and see Additional File 7), indicating that most AIS genes were retained as duplicates from 2R-WGD.

Our outgroup comparison analysis suggested the category NS as the ancestral function of most AIS genes (Table 2), as in a previous study [22]. Although suggested by several previous studies [22-25], the apparent association between the AIS and NS may have arisen simply because the number of genes involved in the NS is larger than that of genes involved in other systems. We therefore examined the relative enrichment of assigned categories among AIS subfamilies as compared with the overall distribution of these categories among all subfamilies. This analysis revealed that the NS category was significantly over-represented as an ancestral function among AIS subfamilies, supporting statistically the hypothesis [22] that AIS-related signaling genes were ancestrally involved in the development and/or function of the NS in urbilaterians.

Our study did not address the specific evolutionary roles of each round of 2R-WGD. Because of the unavailability of the genome sequence of jawless vertebrates, which presumably diverged from jawed vertebrates before the second round of 2R-WGD [26], it is difficult to differentiate the effect of the first round of 2R-WGD in vertebrate genomes from the second. It has recently been shown that jawless vertebrates evolved their own AIS, which is different from the AIS of jawed vertebrates [27,28]. We anticipate that the ongoing genome project (Washington University, St Louis, MO, USA) of a jawless vertebrate (lamprey) will clarify the individual roles of each round of 2R-WGD in the evolution of the different AISs of these organisms.

To date, studies using small-scale gene sets have hinted at relationships between the AIS and 2R-WGD [2,10,22] and between the AIS and NS [22-25]. Although such studies are informative, they cannot yield conclusions that are broadly applicable to the role of 2R-WGD. However, our use of large-scale data sets (i.e., genomic sequence information, microarray-based expression data, and functional annotation) enabled us to address these relationships in a genome-wide fashion. In future research, integration of other large-scale data sets (e.g., other types of data and from additional species) into similar analyses will facilitate efforts to describe the evolution of the AIS precisely and in detail and will help to further unravel the biologic significance of 2R-WGD in that process.

## Conclusion

The occurrence of 2R-WGD preceding emergence of the AIS enhances our understanding of the biologic significance of WGD. Comprehensive identification of the paralogons in the human genome enabled us to conduct a genomic mapping analysis to examine the relationship between 2R-WGD and the evolution of many of the genes involved in the AIS. This analysis revealed that numerous pairs of signaling genes in the AIS (i.e., AIS genes) and their paralogs were part of paralogons, indicating that the genes were retained as duplicates from 2R-WGD.

The large-scale biologic data enabled us to examine the statistical significance of enrichment of system-level function categories among the AIS subfamilies as compared with the overall distribution of these categories among all subfamilies. This examination revealed that the category NS was significantly over-represented among AIS subfamilies, supporting statistically the hypothesis that the AIS-related signaling genes were involved in the nervous system of ancestral bilaterians. Our analysis uncovered the evolutionary role of 2R-WGD in duplicating numerous signaling genes used in the ancestral NS and thereafter in leading to the coordinated evolution of the resulting duplicates to gain new functions in the AIS. We believe that our findings provide a basis for further detailed exploration of the diverse roles of 2R-WGD in the evolutionary success of the vertebrate lineage.

## Methods

### Data preparation

Protein-coding sequences for *Homo sapiens *(human; version 42.36d), *Mus musculus *(mouse; version 42.36c), *Oryzias latipes *(medaka; version 44.1a), and *Drosophila melanogaster *(fly; version 42.43) were obtained from the Ensembl project website [29], and those for *Ciona intestinalis *came from the JGI website [30]. For genes with multiple transcripts, only the longest sequence was retrieved, resulting in 15 852 *Ciona*, 24 125 mouse, 19 938 medaka, 13 545 *Drosophila*, and 22 102 human protein-coding genes (total, 95 562 genes).

### Clustering for detecting subfamilies of chordates

The objective of gene clustering was to reconstruct groups of genes such that each included all of (and only) the descendents of a single gene in the ancestral chordate. The underlying assumption was that all of the vertebrate genes in such a group were more similar to each other than to their ortholog in *Ciona*, because they arose by either gene duplication or lineage splitting after the urochordate-vertebrate divergence. We generated the translated protein sequences for all genes, conducted all-to-all BLASTP analysis using the default parameters [31], and identified reciprocal best-hit pairs between *Ciona *and vertebrate protein sequences (*C *and *V*, respectively). Such a pair recursively recruited other vertebrate sequences of which the best hit in *Ciona *was *C *if they were more similar to each other than *C *was to *V*; this constraint ensured that genes with similarity due to duplication before the urochordate-vertebrate divergence were allocated appropriately into separate groups. The resulting groups of genes were defined as subfamilies. We consequently generated 7109 subfamilies that included 39 407 (48%) of the 82 017 total chordate genes.

### Phylogenetic analysis for subfamilies

For multiple sequence alignment of each subfamily, MAFFT 5.852 [32] was run using the parameters: global alignment option; model, BLOSUM62; maximal iteration, 1000. This alignment then was trimmed by eliminating all positions with gap characters. Only a multiple alignment with a remaining length of at least 100 amino acids was used for further analysis. Phylogenetic trees were inferred by a maximum likelihood method as implemented in TREE-PUZZLE 5.2 [33] with the JTT model of amino acid substitution [34] and a gamma distribution of rates over eight rate categories; 10 000 puzzling steps were used to assess reliability. Any trees with nodes that did not bifurcate strictly were eliminated.

### Paralogon detection and genomic mapping analysis

By using strictly bifurcating trees, we retrieved gene duplications that occurred before the divergence of fish and tetrapods and identified 2774 paralogous pairs of genes in humans. Paralogous pairs duplicated at the base of the vertebrate branch were defined as BV paralogous pairs. A paralogon was defined as two separate regions in the same genome, each having one gene of each of two (or more) BV paralogous pairs, with a maximum of 100 unduplicated genes between the two BV paralogous genes. Through a sliding window analysis (for details, see [8]), we comprehensively identified paralogons in the human genome. For any paralogous gene pair *P*, we examined whether the genomic map position of *P *was part of one of the paralogons.

### Clustering for detecting subfamilies of bilaterians

Although the general procedure was similar to the algorithm for detecting subfamilies of chordate genes, two additional modifications were incorporated. First, we used genes of flies instead of those of *Ciona*. Second, we took account of paralogous genes in *Drosophila *that were duplicated in the lineage leading to *Drosophila*. After clustering groups with complete gene sets of one invertebrate (*Drosophila*) and three vertebrates in the same way as for detecting subfamilies of chordate genes, we obtained singleton *Drosophila *sequences that were not included in any subfamily. For any singleton *Drosophila *sequence *S*, if *S *was more similar to the *Drosophila *sequence *D *in a subfamily than *D *was to any vertebrate sequences in the subfamily, then *S *was re-recruited into the subfamily.

### Gene Ontology

GO terms [35] assigned to human genes were downloaded from the Ensembl project website [29] (Dec 2006). By mapping the GO terms into the more general parent GO Slim terms (Generic GO Slim; obtained from the website of the Gene Ontology consortium [36]), we assigned GO Slim terms to each human gene. We focused on the descendant terms of "biological process" (GO: 0008150).

### Normalization of microarray expression data

Large-scale microarray expression data for 79 human tissues and organs were obtained from the GEO website [37,38] (GEO accession ID: GDS596). We excluded probes for control sequences and those with names that carried the suffix_x_at; such probes do not uniquely complement the target sequence and hence are likely to cross-hybridize. For resulting data, the arithmetic mean of the two replicates in the microarray data was calculated. Next, the data were normalized (Z-score normalization) according to sample to exclude differences associated with sample preparation, for example, and then further normalized (Z-score normalization) according to probe to exclude factors such as housekeeping genes, which are highly expressed in all tissues and organs. If the Z-score of a gene in a particular tissue or organ was greater than or equal to 2, we considered that the gene was expressed specifically in that tissue or organ.

### Listing the NS- and AIS-related functions of human genes in the AIS subfamilies according to data in the literature

Information regarding the NS- and AIS-related functions of human genes in the AIS subfamilies was assigned on the basis of published literature (Dec 2006). Most of the literature data supporting the functions of human genes are based on analyses that have used their orthologs in other vertebrate species (e.g., mouse, chicken, rat). The assigned information regarding function reflected one of two types of data. The first type of assigned information was that concluded from phenotypical changes in experiments, including those involving gene mutation or knockout, overexpression or ectopic expression of wild-type or mutant genes, antisense RNA or RNAi, specific protein inhibitors, or gene interaction and rescue. However, for precise assignment, experiments involving multiple members of a single subfamily (e.g., double knockout experiments) were not considered. The second type of annotated information was that concluded from studies addressing causes of diseases.

### Assignment of system-level function categories to human genes in the AIS subfamilies on the basis of microarray data

System-level function categories were assigned to the human genes in the subfamilies according to the following criterion: if, on the basis of microarray data (GEO accession ID: GDS596), a gene is specifically expressed in at least one of the tissues or organs represented in a system-level function category, then that system-level function category was assigned to the gene.

### Assignment of system-level function categories to *Drosophila *genes on the basis of FlyBase annotation

To assign system-level function categories to *Drosophila *genes, we used allele phenotype data [18] that are manually curated with hierarchically structured controlled vocabularies (CVs) in the FlyBase database [39]. The CV term "nervous system" (FBbt:00005093) corresponded to the system-level function category NS, that of "muscle system" (FBbt:00005069) corresponded to "muscle tissue", and that of "circulatory system" (FBbt:00005057) corresponded to "blood." The CV terms "lamellocyte" (FBbt:00001687), "crystal cell" (FBbt:00001690), and "hemocyte" (FBbt:00005063) corresponded to the system-level function category "innate immunity." If a *Drosophila *gene in a subfamily was annotated with the CV corresponding to a system-level function category or with any of the descendents of the CV, then the corresponding system-level function category was assigned to the gene.

## Abbreviations

AIS: adaptive immune system; 2R-WGD: two rounds of whole-genome duplication; GO: Gene Ontology; CV: controlled vocabulary; NS: nervous system.

## Authors' contributions

KO performed all of the experiments and the data analyses. KO and KA wrote the manuscript.

## Supplementary Material

Additional file 1 Families related to the adaptive immune system. †The number after "TF" uniquely identifies the phylogenetic tree in the TreeFam database; we listed the family members that formed a single clade in the cited phylogenetic tree. "Literature" indicates that the listed family members were obtained by referring to published data. ‡The number of subfamilies included in the described family. Click here for file

Additional file 2 Functions of AIS subfamily members in the AIS. Hs, *Homo sapiens*; Dm, *Drosophila melanogaster*; AIS, adaptive immune system. Click here for file

Additional file 3 AIS subfamilies and Ensembl IDs. Hs, *Homo sapiens*; Dm, *Drosophila melanogaster*; AIS, adaptive immune system. Click here for file

Additional file 4 Phylogenetic trees of AIS subfamilies. AIS, adaptive immune system. The name of each AIS subfamily is in boldface, and each operational taxonomic unit (OTU) is labeled with its Ensembl ID by using the following species abbreviations: Dm, *Drosophila melanogaster*; Ol, *Oryzias latipes*; Hs, *Homo sapiens*; Mm, *Mus musculus*. Support values found by TREE-PUZZLE are at interior nodes. The scale bar indicates substitutions per site. The consensus tree for each AIS subfamily was drawn by using NJplot. Click here for file

Additional file 5 Timing of duplications in AIS subfamilies. *The duplications in each AIS subfamily are counted on the basis of its phylogenetic tree. Hs, *Homo sapiens*; AIS, adaptive immune system. Click here for file

Additional file 6 Paralogous pairs that are part of paralogons. Hs, *Homo sapiens*; AIS, adaptive immune system. Click here for file

Additional file 7 Genomic maps of all AIS subfamilies. AIS, adaptive immune system. The chromosomes of the human genome from chromosome 1 (chr1) to chromosomes X and Y (chrX, Y) are arranged in a circle (blue line). Red lines connect the genomic map positions of the human genes in an AIS subfamily. A gray line connects the genomic map positions of the genes in each BV paralogous pair (a paralogous pair that were formed at the Base of the Vertebrate lineage) of paralogons. Click here for file

Additional file 8 Tissue/organ classification scheme. Click here for file

Additional file 9 System-level function category profile of AIS subfamilies. * *P *values are calculated by using hypergeometric distribution (before Bonferroni correction). BM, bone marrow; MT, muscle tissue; RepO, reproductive organ; ResO, respiration organ; Epi, epithelium; IO, internal organ and metabolism system; AIS, adaptive immune system; II, innate immunity; NS, Nerve system. System-level function category *C *is assigned to subfamily *F *if and only if *F *is expressed in at least one tissue or organ that is classified into *C*. "+" indicates that the system-level function category is assigned to the subfamily; otherwise "-" is indicated. Click here for file

Additional file 10 Assignment of system-level function categories to human and *Drosophila *genes in AIS subfamilies by using microarray expression data and FlyBase annotations. "+" indicates that a human gene is specifically expressed (Z-score ≧ 2) in at least one tissue or organ that is classified into the listed system-level function category; otherwise, "-" is indicated. For *Drosophila *genes, anatomic ontology terms are listed according to FlyBase annotation; descriptions of terms are given in parentheses. Dm, *Drosophila melanogaster*; Hs, *Homo sapiens*; AIS, adaptive immune system. Click here for file

Additional file 11 Assignment of system-level function categories to human and *Drosophila *genes in AIS subfamilies according to published data and FlyBase annotations. For *Drosophila *genes, anatomic ontology terms are listed according to FlyBase annotation; descriptions of terms are given in parentheses. Dm, *Drosophila melanogaster*; Hs, *Homo sapiens*; AIS, adaptive immune system. Click here for file
