# Supplementary material for: Expansion of signaling genes for adaptive immune system evolution in early vertebrates
Source: BMC Genomics. 2008 May 14;9:218. doi: 10.1186/1471-2164-9-218 (PMC2391169; doi:10.1186/1471-2164-9-218)
Supplement: Additional file 1 — Families related to the adaptive immune system. †The number after "TF" uniquely identifies the phylogenetic tree in the TreeFam database; we listed the family members that formed a single clade in the cited phylogenetic tree. "Literature" indicates that the listed family members were obtained by referring to published data. ‡The number of subfamilies included in the described family. [file 1471-2164-9-218-S1.doc]

| **Additional file 1. Families related to the adaptive immune system** | | |  |  |
| --- | --- | --- | --- | --- |
| Family name | | Family member | Supporting information† | Number of subfamilies‡ |
| JAK | (Janus kinase) | *JAK1 JAK2 JAK3 TYK2* | TF327041 | 1 |
| PIAS | (protein inhibitor of activated stats) | *PIAS1 PIAS2 PIAS3 PIAS4* | TF323787 | 1 |
| STAT | (signal transducer and activator of transcription) | *STAT1 STAT2 STAT3 STAT4 STAT5A STAT5B STAT6* | TF318648 | 1 |
| SOCS | (suppressor of cytokine signaling) | *CISH SOCS1 SOCS2 SOCS3 SOCS4 SOCS5 SOCS6 SOCS7* | TF321368 | 2 |
| SHP | (SH2 domain-containing protein tyrosine phosphatase) | *PTPN6 PTPN11 PTPN12 PTPN22* | TF318263 | 1 |
| PRKAR | (protein kinase A, regulatory subunit) | *PRKAR1A PRKAR1B PRKAR2A PRKAR2B* | TF314920 | 2 |
| PRKAC | (protein kinase A, catalytic subunit) | *PRKACA PRKACB PRKACG* | TF313399 | 1 |
| PDE | (phosphodiesterase) | *PDE1A PDE1B PDE1C PDE4A PDE4B PDE4C PDE4D PDE7A PDE7B PDE8A PDE8B PDE9A* | TF314638 | 4 |
| ADCY | (adenylate cyclase) | *ADCY1 ADCY2 ADCY3 ADCY4 ADCY5 ADCY6 ADCY7 ADCY8 ADCY9* | TF313845 | 4 |
| GNG | (guanine nucleotide binding protein, gamma subunit) | *GNG2 GNG3 GNG4 GNG5 GNG7 GNG8 GNG10 GNG11 GNG12 GNG13 GNGT1 GNGT2* ENSG00000182625 ENSG00000133136 | TF319909 | 2 |
| GNB | (guanine nucleotide binding protein, beta subunit) | *GNB1 GNB2 GNB3 GNB4 GNB5* | TF106149 | 2 |
| GNA | (guanine nucleotide binding protein, alpha subunit) | *GNA11 GNA12 GNA13 GNA14 GNA15 GNAI1 GNAI2 GNAI3 GNAL GNAO1 GNAQ GNAS GNAT1 GNAT2 GNAZ* | TF300673 | 5 |
| RGS | (regulator of G-protein signaling) | *RGS1 RGS2 RGS3 RGS4 RGS5 RGS6 RGS7 RGS8 RGS9 RGS10 RGS11 RGS13 RGS16 RGS17 RGS18 RGS19 RGS20* | TF315837 | 3 |
| RHO | (ras homolog gene family) | *RHOA RHOB RHOC* | TF300837 | 1 |
| DGK | (diacylglycerol kinase) | *DGKA DGKB DGKG DGKD DGKE DGKH DGKQ DGKK* | TF313104 | 4 |
| PLCB | (phospholipase C, beta) | *PLCB1 PLCB2 PLCB3 PLCB4* | TF313216 | 1 |
| PLCG | (phospholipase C, gamma) | *PLCG1 PLCG2* | TF313216 | 1 |
| aPKC | (atypical protein kinase C) | *PRKCZ PRKCI* | TF102004 | 1 |
| nPKC | (novel protein kinase C) | *PRKCD PRKCE PRKCH PRKCQ* | TF102004 | 2 |
| cPKC | (classical protein kinase C) | *PRKCA PRKCB1 PRKCG* | TF102004 | 1 |
| PKD | (protein kinase D) | *PRKD1 PRKD2 PRKD3* | TF314320 | 1 |
| CAMKK | (Ca2+/calmodulin kinase kinase) | *CAMKK1 CAMKK2* | TF313013 | 1 |
| CAMK1 | (Ca2+/calmodulin kinase I) | *CAMK1 CAMK1G CAMK1D PNCK* | TF501392 | 1 |
| CAMK2 | (Ca2+/calmodulin kinase II) | *CAMK2A CAMK2B CAMK2G CAMK2D* | TF501392 | 1 |
| CALM | (calmodulin) | *CALM1 CALM2 CALML3*  CALM_HUMAN | TF300912 | 1 |
| CALNA | (calcineurin, catalytic subunit) | *PPP3CA PPP3CB PPP3CC* | TF105557 | 1 |
| CALNB | (calcineurin, regulatory subunit) | *PPP3R1 PPP3R2* | TF105558 | 1 |
| NFAT | (nuclear factor of activated T-cells) | *NFAT5 NFATC1 NFATC2 NFATC3 NFATC4* | TF326480 | 1 |
| IKBK | (inhibitor of kappa light polypeptide gene enhancer in B-cells kinase) | *CHUK IKBKB IKBKE TBK1* | TF324269 | 2 |
| NFKB | (nuclear factor of kappa light polypeptide gene enhancer in B-cells) | *NFKB1 NFKB2 REL RELA RELB* | TF325632 | 1 |
| NFKBI | (nuclear factor of kappa light polypeptide gene enhancer in B-cells inhibitor) | *BCL3 NFKBIA NFKBIB NFKBIE* | TF320166 | 1 |
| PIK3C | (phosphoinositide-3-kinase, catalytic subunit) | *PIK3CA PIK3CB PIK3CG PIK3CD PIK3C2A PIK3C2B PIK3C2G* | TF102031 | 2 |
| PIK3R | (phosphoinositide-3-kinase, regulatory subunit) | *PIK3R1 PIK3R2 PIK3R3* | TF102033 | 1 |
| PTEN | (phosphatase and tensin homolog) | *PTEN* | TF324513 | 1 |
| AKT | (v-akt murine thymoma viral oncogene homolog) | *AKT1 AKT2 AKT3* | TF102004 | 1 |
| SYK | (spleen tyrosine kinase) | *SYK ZAP70* | TF316643 | 0 |
| SRC | (v-src sarcoma viral oncogene homolog) | *BLK CSK FGR FRK FYN HCK LCK LYN MATK PTK6 SRC SRMS YES1* | TF314013 | 2 |
| ABL | (v-abl Abelson murine leukemia viral oncogene homolog) | *ABL1 ABL2* | TF105081 | 1 |
| TEC | (tec protein tyrosine kinase) | *BMX BTK ITK TEC TXK* | TF315363 | 1 |
| GRB2 | (growth factor receptor-bound protein 2) | *GRAP GRAP2 GRB2* | TF314013 | 1 |
| BLNK | (B-cell linker) | *BLNK* | TF326567 | 1 |
| LCP2 | (lymphocyte cytosolic protein 2) | *LCP2* | TF326567 | 0 |
| SOS | (son of sevenless homolog) | *SOS1 SOS2* | TF315204 | 1 |
| RAS | (ras oncogene family) | *HRAS KRAS NRAS* | TF312796 | 2 |
| RAF | (v-raf murine sarcoma viral oncogene homolog) | *ARAF BRAF RAF1* | TF317006 | 1 |
| ELK | (member of ETS oncogene family) | *ELK1 ELK3 ELK4* | TF317732 | 0 |
| p38 | (mitogen-activated protein kinase) | *MAPK11 MAPK12 MAPK13 MAPK14* | TF105100 | 1 |
| FOS | (v-fos FBJ murine osteosarcoma viral oncogene homolog) | *FOS FOSB FOSL1 FOSL2* | TF326301 | 1 |
| JUN | (v-jun avian sarcoma virus 17 oncogene homolog) | *JUN JUNB JUND* | TF323952 | 1 |
| MAP3K | (mitogen-activated protein kinase kinase kinase) | *MAP3K1 MAP3K2 MAP3K3 MAP3K4 MAP3K5 MAP3K6 MAP3K7* | literature [1] | 3 |
| JNK | (c-Jun N-terminal kinase) | *MAPK8 MAPK9 MAPK10* | TF105100 | 1 |
| aMAPK | (atypical mitogen-activated protein kinase) | *MAPK4 MAPK6 MAPK15 NLK* | literature [1] | 2 |
| cMAPK | (classical mitogen-activated protein kinase) | *MAPK1 MAPK3* | TF105097 | 1 |
| MAP2K | (mitogen-activated protein kinase kinase) | *MAP2K1 MAP2K2 MAP2K3 MAP2K4 MAP2K5 MAP2K6 MAP2K7* | literature [1] | 4 |
| RAC | (ras-related C3 botulinum toxin substrate) | *RAC1 RAC2 RAC3* ENSG00000172895 ENSG00000134598 | TF101109 | 1 |
| CDC42 | (cell division control protein 42 homolog precursor) | *CDC42* ENSG00000152994 | TF101109 | 1 |
| RAP1 | (ras-related protein) | *RAP1A RAP1B*  ENSG00000176276 | TF313014 | 1 |
| VAV | (vav oncogene) | *VAV1 VAV2 VAV3* | TF316171 | 1 |
| RASGRP | (ras guanyl releasing protein) | *RASGRP1 RASGRP2 RASGRP3 RASGRP4* | TF312918 | 0 |
| SHC | (SHC-transforming protein) | *SHC1 SHC2 SHC3 SHC4* | TF315807 | 1 |
| GAB | (GRB2-associated-binding protein) | *GAB1 GAB2 GAB3* | TF329487 | 1 |
| †The number after “TF” uniquely identifies the phylogenetic tree in the TreeFam database [2]; we listed the family members that formed a single clade in the cited phylogenetic tree. “Literature” indicates that the listed family members were obtained by referring to published data. | | | | |
| ‡The number of subfamilies included in the described family. | | | | |

**Supplementary References**

1. Coulombe P, Meloche S: **Atypical mitogen-activated protein kinases: Structure, regulation and functions**. *Biochim Biophys Acta* 2007, **1773**:1376-1387.

2. Li H, Coghlan A, Ruan J, Coin LJ, Heriche JK, Osmotherly L, Li R, Liu T, Zhang Z, Bolund L *et al*: **TreeFam: a curated database of phylogenetic trees of animal gene families**. *Nucleic Acids Res* 2006, **34**(Database issue):D572-580.
