# Supplementary material for: Expansion of signaling genes for adaptive immune system evolution in early vertebrates
Source: BMC Genomics. 2008 May 14;9:218. doi: 10.1186/1471-2164-9-218 (PMC2391169; doi:10.1186/1471-2164-9-218)
Supplement: Additional file 2 — Functions of AIS subfamily members in the AIS. Hs, Homo sapiens; Dm, Drosophila melanogaster; AIS, adaptive immune system. [file 1471-2164-9-218-S2.doc]

| **Additional file 2. Functions of AIS subfamily members in the AIS** | | | |
| --- | --- | --- | --- |
| AIS subfamily | Species | Member | Function in the AIS |
| JAK | Hs | *JAK1* | T and B cell development [1] |
|  | Hs | *JAK2* |  |
|  | Hs | *JAK3* | T and B cell development [2] |
|  | Dm | *hop* |  |
| PIAS | Hs | *PIAS1* |  |
|  | Hs | *PIAS2* | interleukin-12 signal transduction in T cells [3] |
|  | Hs | *PIAS3* |  |
|  | Hs | *PIAS4* |  |
|  | Dm | *Su(var)2-10* |  |
| STAT | Hs | *STAT5A* | T cell differentiation [4] |
|  | Hs | *STAT5B* | T cell proliferation and B cell development [5] |
|  | Hs | *STAT6* | T cell differentiation and immunoglobulin class switch in B cells [6]; B cell migration [7] |
|  | Dm | *Stat92E* |  |
| SOCS | Hs | *SOCS4* |  |
|  | Hs | *SOCS5* | T cell differentiation [8] |
|  | Dm | *Socs36E* |  |
| SHP | Hs | *PTPN11* | T cell development and proliferation [9] |
|  | Hs | *PTPN6* | T cell differentiation [10] |
|  | Dm | *csw* |  |
| PRKAR | Hs | *PRKAR1A* | responsibility for systemic lupus erythematosus [11] |
|  | Hs | *PRKAR1B* |  |
|  | Dm | *Pka-R1* |  |
| GNG | Hs | *GNG12* |  |
|  | Hs | *GNG2* |  |
|  | Hs | *GNG3* | antibody responses [12] |
|  | Hs | *GNG4* |  |
|  | Hs | *GNG5* |  |
|  | Hs | *GNG8* |  |
|  | Dm | *Ggamma1* |  |
| GNB | Hs | *GNB1* |  |
|  | Hs | *GNB2* |  |
|  | Hs | *GNB3* | T cell proliferation and migration [13] |
|  | Hs | *GNB4* |  |
|  | Dm | *Gbeta13F* |  |
| GNA | Hs | *GNAI1* |  |
|  | Hs | *GNAI2* | B cell development [14]; proliferation and cytokine production in T cells [15] |
|  | Hs | *GNAI3* |  |
|  | Dm | *G-ialpha65A* |  |
| RHO | Hs | *RHOA* | T cell adhesion [16] |
|  | Hs | *RHOC* |  |
|  | Dm | *Rho1* |  |
| DGK | Hs | *DGKA* | T cell anergy [17] |
|  | Hs | *DGKB* |  |
|  | Hs | *DGKG* |  |
|  | Dm | *Dgk* |  |
| PLCG | Hs | *PLCG1* | development and proliferation in B cells [18] |
|  | Hs | *PLCG2* | B cell development [18] |
|  | Dm | *sl* |  |
| aPKC | Hs | *PRKCI* |  |
|  | Hs | *PRKCZ* | differentiation and cytokine production in T cells [19]; proliferation and survival in B cells [20] |
|  | Dm | *aPKC* |  |
| nPKC | Hs | *PRKCD* | interleukin-6 production and proliferation in B cells [21] |
|  | Hs | *PRKCQ* | interleukin-2 production and proliferation in T cells [22] |
|  | Dm | *Pkcdelta* |  |
| cPKC | Hs | *PRKCA* | degranulation in cytotoxic T lymphocytes [23] |
|  | Hs | *PRKCB1* | B cell survival [24] |
|  | Hs | *PRKCG* |  |
|  | Dm | *Pkc53E* |  |
| CAMK2 | Hs | *CAMK2A* |  |
|  | Hs | *CAMK2B* |  |
|  | Hs | *CAMK2D* |  |
|  | Hs | *CAMK2G* | differentiation and survival in T cells [25] |
|  | Dm | *CaMKII* |  |
| CALNA | Hs | *PPP3CA* | T cell proliferation [26] |
|  | Hs | *PPP3CB* | development and proliferation in T cells [27] |
|  | Dm | *CanA-14F* |  |
|  | Dm | *Pp2B-14D* |  |
| CALNB | Hs | *PPP3R1* | T cell development [28] |
|  | Dm | CG14353 |  |
| NFAT | Hs | *NFAT5* | T cell development [29] |
|  | Dm | *NFAT* |  |
| IKBK | Hs | *CHUK* | development and immunoglobulin production in B cells [30]; T cell survival [31] |
|  | Hs | *IKBKB* | T cell proliferation and survival [31]; survival and immunoglobulin production in B cells [32] |
|  | Dm | *ird5* |  |
| NFKB | Hs | *NFKB1* | T cell differentiation [33] |
|  | Hs | *NFKB2* | differentiation and cytokine production in T cells [34] |
|  | Hs | *REL* | T cell differentiation [33]; B cell proliferation and survival [35] |
|  | Hs | *RELA* | immunoglobulin production in B cells [36] |
|  | Hs | *RELB* | differentiation and cytokine production in T cells [34] |
|  | Dm | *dl* |  |
|  | Dm | *Dif* |  |
| NFKBI | Hs | *BCL3* | differentiation and cytokine production in T cells [34] |
|  | Hs | *NFKBIA* | T cell proliferation; proliferation and immunoglobulin production in B cells [37] |
|  | Hs | *NFKBIB* |  |
|  | Hs | *NFKBIE* | T cell development [38] |
|  | Dm | *cact* |  |
| PIK3C | Hs | *PIK3CA* |  |
|  | Hs | *PIK3CB* |  |
|  | Hs | *PIK3CD* | B cell migration [39] |
|  | Dm | *Pi3K92E* |  |
| PIK3R | Hs | *PIK3R1* | B cell development [40] |
|  | Hs | *PIK3R2* | proliferation and survival in T cells [41] |
|  | Hs | *PIK3R3* |  |
|  | Dm | *Pi3K21B* |  |
| PTEN | Hs | *PTEN* | migration, proliferation and survival in B cells [42] |
|  | Dm | *Pten* |  |
| AKT | Hs | *AKT1* | T cell activation [43] |
|  | Hs | *AKT2* |  |
|  | Hs | *AKT3* |  |
|  | Dm | *Akt1* |  |
| SRC | Hs | *CSK* | T cell development [44] |
|  | Hs | *MATK* | development and cytokine production in B cells [45] |
|  | Dm | *csk* |  |
| ABL | Hs | *ABL1* | B cell proliferation [46] |
|  | Hs | *ABL2* |  |
|  | Dm | *Abl* |  |
| TEC | Hs | *BMX* |  |
|  | Hs | *BTK* | B cell development [47] |
|  | Hs | *ITK* | cytokine production and differentiation in T cells [48] |
|  | Hs | *TEC* | T cell activation [49] |
|  | Hs | *TXK* | development and cytokine production in T cells [50] |
|  | Dm | *Btk29A* |  |
| GRB2 | Hs | *GRAP* | proliferation and cytokine production in T cells [51] |
|  | Hs | *GRB2* | T cell development [52] |
|  | Dm | *drk* |  |
| BLNK | Hs | *BLNK* | B cell development [53] |
|  | Dm | CG15529 |  |
| SOS | Hs | *SOS1* | T cell proliferation [54] |
|  | Hs | *SOS2* |  |
|  | Dm | *Sos* |  |
| RAS | Hs | *HRAS* | proliferation and development in T cells [55] |
|  | Hs | *KRAS* |  |
|  | Dm | *Ras85D* |  |
| RAF | Hs | *ARAF* |  |
|  | Hs | *BRAF* | cytokine production [56] |
|  | Hs | *RAF1* | interleukin-2 production [57] |
|  | Dm | *phl* |  |
| FOS | Hs | *FOS* | B cell proliferation [58] |
|  | Hs | *FOSB* | T cell proliferation [59] |
|  | Hs | *FOSL2* |  |
|  | Dm | *kay* |  |
| JUN | Hs | *JUN* | interleukin-2 expression in T cells [60] |
|  | Hs | *JUNB* | differentiation and cytokine production in T cells [61] |
|  | Hs | *JUND* | proliferation, differentiation and cytokine production in T cells [62] |
|  | Dm | *Jra* |  |
| MAP3K-1 | Hs | *MAP3K7* | B cell proliferation [63] |
|  | Dm | *Tak1* |  |
| MAP3K-2 | Hs | *MAP3K4* | cytokine production in T cells [64] |
|  | Dm | *Mekk1* |  |
| JNK | Hs | *MAPK10* |  |
|  | Hs | *MAPK8* | differentiation, cytokine production and proliferation in T cells [65] |
|  | Hs | *MAPK9* | differentiation and cytokine production in T cells [66] |
|  | Dm | *bsk* |  |
| cMAPK | Hs | *MAPK1* | T cell development [67] |
|  | Hs | *MAPK3* | T cell development [68] |
|  | Dm | *rl* |  |
| MAP2K-1 | Hs | *MAP2K3* | T cell survival [69] |
|  | Hs | *MAP2K6* | T cell survival [69] |
|  | Dm | *lic* |  |
| MAP2K-2 | Hs | *MAP2K1* | T cell development [70] |
|  | Hs | *MAP2K2* |  |
|  | Dm | *Dsor1* |  |
| MAP2K-3 | Hs | *MAP2K7* | cytokine production and proliferation in T cells [71] |
|  | Dm | *hep* |  |
| MAP2K-4 | Hs | *MAP2K4* | T cell development [72]; cytokine production and proliferation in T cells [73] |
|  | Dm | *Mkk4* |  |
| RAC | Hs | ENSG00000172895 |  |
|  | Hs | *RAC1* | T cell development [74] |
|  | Hs | *RAC2* | B cell development [75] |
|  | Hs | *RAC3* |  |
|  | Dm | *Rac1* |  |
|  | Dm | *Rac2* |  |
| CDC42 | Hs | *CDC42* | T cell activation [76] |
|  | Hs | ENSG00000152994 |  |
|  | Dm | *Cdc42* |  |
| RAP1 | Hs | ENSG00000176276 |  |
|  | Hs | *RAP1A* | cytokine production and proliferation in T cells [77] |
|  | Hs | *RAP1B* |  |
|  | Dm | *R* |  |
| VAV | Hs | *VAV1* | development and activation in T cells, B cell development [78] |
|  | Hs | *VAV2* | immunoglobulin production in B cells [79] |
|  | Hs | *VAV3* |  |
|  | Dm | *vav* |  |
| SHC | Hs | *SHC1* | T cell development [80] |
|  | Hs | *SHC2* |  |
|  | Hs | *SHC3* |  |
|  | Hs | *SHC4* |  |
|  | Dm | *Shc* |  |
| GAB | Hs | *GAB1* | development in T cells [81]; development and immunoglobulin production in B cells [81] |
|  | Hs | *GAB2* | activation and cytokine production in T cells [82] |
|  | Hs | *GAB3* |  |
|  | Dm | *dos* |  |
| Hs, *Homo sapiens*; Dm, *Drosophila melanogaster*; AIS, adaptive immune system. | | | |

**Supplementary References**

1. Rodig SJ, Meraz MA, White JM, Lampe PA, Riley JK, Arthur CD, King KL, Sheehan KC, Yin L, Pennica D *et al*: **Disruption of the Jak1 gene demonstrates obligatory and nonredundant roles of the Jaks in cytokine-induced biologic responses**. *Cell* 1998, **93**(3):373-383.

2. Park SY, Saijo K, Takahashi T, Osawa M, Arase H, Hirayama N, Miyake K, Nakauchi H, Shirasawa T, Saito T: **Developmental defects of lymphoid cells in Jak3 kinase-deficient mice**. *Immunity* 1995, **3**(6):771-782.

3. Arora T, Liu B, He H, Kim J, Murphy TL, Murphy KM, Modlin RL, Shuai K: **PIASx is a transcriptional co-repressor of signal transducer and activator of transcription 4**. *J Biol Chem* 2003, **278**(24):21327-21330.

4. Kagami S, Nakajima H, Suto A, Hirose K, Suzuki K, Morita S, Kato I, Saito Y, Kitamura T, Iwamoto I: **Stat5a regulates T helper cell differentiation by several distinct mechanisms**. *Blood* 2001, **97**(8):2358-2365.

5. Burchill MA, Goetz CA, Prlic M, O'Neil JJ, Harmon IR, Bensinger SJ, Turka LA, Brennan P, Jameson SC, Farrar MA: **Distinct effects of STAT5 activation on CD4+ and CD8+ T cell homeostasis: development of CD4+CD25+ regulatory T cells versus CD8+ memory T cells**. *J Immunol* 2003, **171**(11):5853-5864.

6. Shimoda K, van Deursen J, Sangster MY, Sarawar SR, Carson RT, Tripp RA, Chu C, Quelle FW, Nosaka T, Vignali DA *et al*: **Lack of IL-4-induced Th2 response and IgE class switching in mice with disrupted Stat6 gene**. *Nature* 1996, **380**(6575):630-633.

7. Davey EJ, Greicius G, Thyberg J, Severinson E: **STAT6 is required for the regulation of IL-4-induced cytoskeletal events in B cells**. *Int Immunol* 2000, **12**(7):995-1003.

8. Seki Y, Hayashi K, Matsumoto A, Seki N, Tsukada J, Ransom J, Naka T, Kishimoto T, Yoshimura A, Kubo M: **Expression of the suppressor of cytokine signaling-5 (SOCS5) negatively regulates IL-4-dependent STAT6 activation and Th2 differentiation**. *Proc Natl Acad Sci U S A* 2002, **99**(20):13003-13008.

9. Nguyen TV, Ke Y, Zhang EE, Feng GS: **Conditional deletion of Shp2 tyrosine phosphatase in thymocytes suppresses both pre-TCR and TCR signals**. *J Immunol* 2006, **177**(9):5990-5996.

10. Kamata T, Yamashita M, Kimura M, Murata K, Inami M, Shimizu C, Sugaya K, Wang CR, Taniguchi M, Nakayama T: **src homology 2 domain-containing tyrosine phosphatase SHP-1 controls the development of allergic airway inflammation**. *J Clin Invest* 2003, **111**(1):109-119.

11. Laxminarayana D, Khan IU, Kammer G: **Transcript mutations of the alpha regulatory subunit of protein kinase A and up-regulation of the RNA-editing gene transcript in lupus T lymphocytes**. *Lancet* 2002, **360**(9336):842-849.

12. Dubeykovskiy A, McWhinney C, Robishaw JD: **Runx-dependent regulation of G-protein gamma3 expression in T-cells**. *Cell Immunol* 2006, **240**(2):86-95.

13. Lindemann M, Virchow S, Ramann F, Barsegian V, Kreuzfelder E, Siffert W, Muller N, Grosse-Wilde H: **The G protein beta3 subunit 825T allele is a genetic marker for enhanced T cell response**. *FEBS Lett* 2001, **495**(1-2):82-86.

14. Dalwadi H, Wei B, Schrage M, Spicher K, Su TT, Birnbaumer L, Rawlings DJ, Braun J: **B cell developmental requirement for the G alpha i2 gene**. *J Immunol* 2003, **170**(4):1707-1715.

15. Huang TT, Zong Y, Dalwadi H, Chung C, Miceli MC, Spicher K, Birnbaumer L, Braun J, Aranda R: **TCR-mediated hyper-responsiveness of autoimmune Galphai2(-/-) mice is an intrinsic naive CD4(+) T cell disorder selective for the Galphai2 subunit**. *Int Immunol* 2003, **15**(11):1359-1367.

16. Vielkind S, Gallagher-Gambarelli M, Gomez M, Hinton HJ, Cantrell DA: **Integrin regulation by RhoA in thymocytes**. *J Immunol* 2005, **175**(1):350-357.

17. Zha Y, Marks R, Ho AW, Peterson AC, Janardhan S, Brown I, Praveen K, Stang S, Stone JC, Gajewski TF: **T cell anergy is reversed by active Ras and is regulated by diacylglycerol kinase-alpha**. *Nat Immunol* 2006, **7**(11):1166-1173.

18. Wen R, Chen Y, Schuman J, Fu G, Yang S, Zhang W, Newman DK, Wang D: **An important role of phospholipase Cgamma1 in pre-B-cell development and allelic exclusion**. *Embo J* 2004, **23**(20):4007-4017.

19. Martin P, Villares R, Rodriguez-Mascarenhas S, Zaballos A, Leitges M, Kovac J, Sizing I, Rennert P, Marquez G, Martinez AC *et al*: **Control of T helper 2 cell function and allergic airway inflammation by PKCzeta**. *Proc Natl Acad Sci U S A* 2005, **102**(28):9866-9871.

20. Martin P, Duran A, Minguet S, Gaspar ML, Diaz-Meco MT, Rennert P, Leitges M, Moscat J: **Role of zeta PKC in B-cell signaling and function**. *Embo J* 2002, **21**(15):4049-4057.

21. Miyamoto A, Nakayama K, Imaki H, Hirose S, Jiang Y, Abe M, Tsukiyama T, Nagahama H, Ohno S, Hatakeyama S *et al*: **Increased proliferation of B cells and auto-immunity in mice lacking protein kinase Cdelta**. *Nature* 2002, **416**(6883):865-869.

22. Sun Z, Arendt CW, Ellmeier W, Schaeffer EM, Sunshine MJ, Gandhi L, Annes J, Petrzilka D, Kupfer A, Schwartzberg PL *et al*: **PKC-theta is required for TCR-induced NF-kappaB activation in mature but not immature T lymphocytes**. *Nature* 2000, **404**(6776):402-407.

23. Pardo J, Buferne M, Martinez-Lorenzo MJ, Naval J, Schmitt-Verhulst AM, Boyer C, Anel A: **Differential implication of protein kinase C isoforms in cytotoxic T lymphocyte degranulation and TCR-induced Fas ligand expression**. *Int Immunol* 2003, **15**(12):1441-1450.

24. Su TT, Guo B, Kawakami Y, Sommer K, Chae K, Humphries LA, Kato RM, Kang S, Patrone L, Wall R *et al*: **PKC-beta controls I kappa B kinase lipid raft recruitment and activation in response to BCR signaling**. *Nat Immunol* 2002, **3**(8):780-786.

25. Bui JD, Calbo S, Hayden-Martinez K, Kane LP, Gardner P, Hedrick SM: **A role for CaMKII in T cell memory**. *Cell* 2000, **100**(4):457-467.

26. Zhang BW, Zimmer G, Chen J, Ladd D, Li E, Alt FW, Wiederrecht G, Cryan J, O'Neill EA, Seidman CE *et al*: **T cell responses in calcineurin A alpha-deficient mice**. *J Exp Med* 1996, **183**(2):413-420.

27. Bueno OF, Brandt EB, Rothenberg ME, Molkentin JD: **Defective T cell development and function in calcineurin A beta -deficient mice**. *Proc Natl Acad Sci U S A* 2002, **99**(14):9398-9403.

28. Neilson JR, Winslow MM, Hur EM, Crabtree GR: **Calcineurin B1 is essential for positive but not negative selection during thymocyte development**. *Immunity* 2004, **20**(3):255-266.

29. Trama J, Go WY, Ho SN: **The osmoprotective function of the NFAT5 transcription factor in T cell development and activation**. *J Immunol* 2002, **169**(10):5477-5488.

30. Kaisho T, Takeda K, Tsujimura T, Kawai T, Nomura F, Terada N, Akira S: **IkappaB kinase alpha is essential for mature B cell development and function**. *J Exp Med* 2001, **193**(4):417-426.

31. Ren H, Schmalstieg A, van Oers NS, Gaynor RB: **I-kappa B kinases alpha and beta have distinct roles in regulating murine T cell function**. *J Immunol* 2002, **168**(8):3721-3731.

32. Li ZW, Omori SA, Labuda T, Karin M, Rickert RC: **IKK beta is required for peripheral B cell survival and proliferation**. *J Immunol* 2003, **170**(9):4630-4637.

33. Hilliard BA, Mason N, Xu L, Sun J, Lamhamedi-Cherradi SE, Liou HC, Hunter C, Chen YH: **Critical roles of c-Rel in autoimmune inflammation and helper T cell differentiation**. *J Clin Invest* 2002, **110**(6):843-850.

34. Corn RA, Hunter C, Liou HC, Siebenlist U, Boothby MR: **Opposing roles for RelB and Bcl-3 in regulation of T-box expressed in T cells, GATA-3, and Th effector differentiation**. *J Immunol* 2005, **175**(4):2102-2110.

35. Feng B, Cheng S, Hsia CY, King LB, Monroe JG, Liou HC: **NF-kappaB inducible genes BCL-X and cyclin E promote immature B-cell proliferation and survival**. *Cell Immunol* 2004, **232**(1-2):9-20.

36. Horwitz BH, Zelazowski P, Shen Y, Wolcott KM, Scott ML, Baltimore D, Snapper CM: **The p65 subunit of NF-kappa B is redundant with p50 during B cell proliferative responses, and is required for germline CH transcription and class switching to IgG3**. *J Immunol* 1999, **162**(4):1941-1946.

37. Chen CL, Singh N, Yull FE, Strayhorn D, Van Kaer L, Kerr LD: **Lymphocytes lacking I kappa B-alpha develop normally, but have selective defects in proliferation and function**. *J Immunol* 2000, **165**(10):5418-5427.

38. Memet S, Laouini D, Epinat JC, Whiteside ST, Goudeau B, Philpott D, Kayal S, Sansonetti PJ, Berche P, Kanellopoulos J *et al*: **IkappaBepsilon-deficient mice: reduction of one T cell precursor subspecies and enhanced Ig isotype switching and cytokine synthesis**. *J Immunol* 1999, **163**(11):5994-6005.

39. Reif K, Okkenhaug K, Sasaki T, Penninger JM, Vanhaesebroeck B, Cyster JG: **Cutting edge: differential roles for phosphoinositide 3-kinases, p110gamma and p110delta, in lymphocyte chemotaxis and homing**. *J Immunol* 2004, **173**(4):2236-2240.

40. Donahue AC, Hess KL, Ng KL, Fruman DA: **Altered splenic B cell subset development in mice lacking phosphoinositide 3-kinase p85alpha**. *Int Immunol* 2004, **16**(12):1789-1798.

41. Deane JA, Trifilo MJ, Yballe CM, Choi S, Lane TE, Fruman DA: **Enhanced T cell proliferation in mice lacking the p85beta subunit of phosphoinositide 3-kinase**. *J Immunol* 2004, **172**(11):6615-6625.

42. Anzelon AN, Wu H, Rickert RC: **Pten inactivation alters peripheral B lymphocyte fate and reconstitutes CD19 function**. *Nat Immunol* 2003, **4**(3):287-294.

43. Kane LP, Mollenauer MN, Weiss A: **A proline-rich motif in the C terminus of Akt contributes to its localization in the immunological synapse**. *J Immunol* 2004, **172**(9):5441-5449.

44. Schmedt C, Saijo K, Niidome T, Kuhn R, Aizawa S, Tarakhovsky A: **Csk controls antigen receptor-mediated development and selection of T-lineage cells**. *Nature* 1998, **394**(6696):901-904.

45. Lee BC, Avraham S, Imamoto A, Avraham HK: **Identification of the nonreceptor tyrosine kinase MATK/CHK as an essential regulator of immune cells using Matk/CHK-deficient mice**. *Blood* 2006, **108**(3):904-907.

46. Zipfel PA, Grove M, Blackburn K, Fujimoto M, Tedder TF, Pendergast AM: **The c-Abl tyrosine kinase is regulated downstream of the B cell antigen receptor and interacts with CD19**. *J Immunol* 2000, **165**(12):6872-6879.

47. Kouro T, Nagata K, Takaki S, Nisitani S, Hirano M, Wahl MI, Witte ON, Karasuyama H, Takatsu K: **Bruton's tyrosine kinase is required for signaling the CD79b-mediated pro-B to pre-B cell transition**. *Int Immunol* 2001, **13**(4):485-493.

48. Schaeffer EM, Yap GS, Lewis CM, Czar MJ, McVicar DW, Cheever AW, Sher A, Schwartzberg PL: **Mutation of Tec family kinases alters T helper cell differentiation**. *Nat Immunol* 2001, **2**(12):1183-1188.

49. Garcon F, Bismuth G, Isnardon D, Olive D, Nunes JA: **Tec kinase migrates to the T cell-APC interface independently of its pleckstrin homology domain**. *J Immunol* 2004, **173**(2):770-775.

50. Sommers CL, Rabin RL, Grinberg A, Tsay HC, Farber J, Love PE: **A role for the Tec family tyrosine kinase Txk in T cell activation and thymocyte selection**. *J Exp Med* 1999, **190**(10):1427-1438.

51. Shen R, Ouyang YB, Qu CK, Alonso A, Sperzel L, Mustelin T, Kaplan MH, Feng GS: **Grap negatively regulates T-cell receptor-elicited lymphocyte proliferation and interleukin-2 induction**. *Mol Cell Biol* 2002, **22**(10):3230-3236.

52. Gong Q, Cheng AM, Akk AM, Alberola-Ila J, Gong G, Pawson T, Chan AC: **Disruption of T cell signaling networks and development by Grb2 haploid insufficiency**. *Nat Immunol* 2001, **2**(1):29-36.

53. Flemming A, Brummer T, Reth M, Jumaa H: **The adaptor protein SLP-65 acts as a tumor suppressor that limits pre-B cell expansion**. *Nat Immunol* 2003, **4**(1):38-43.

54. Salojin K, Zhang J, Cameron M, Gill B, Arreaza G, Ochi A, Delovitch TL: **Impaired plasma membrane targeting of Grb2-murine son of sevenless (mSOS) complex and differential activation of the Fyn-T cell receptor (TCR)-zeta-Cbl pathway mediate T cell hyporesponsiveness in autoimmune nonobese diabetic mice**. *J Exp Med* 1997, **186**(6):887-897.

55. Swan KA, Alberola-Ila J, Gross JA, Appleby MW, Forbush KA, Thomas JF, Perlmutter RM: **Involvement of p21ras distinguishes positive and negative selection in thymocytes**. *Embo J* 1995, **14**(2):276-285.

56. Tsukamoto H, Irie A, Nishimura Y: **B-Raf contributes to sustained extracellular signal-regulated kinase activation associated with interleukin-2 production stimulated through the T cell receptor**. *J Biol Chem* 2004, **279**(46):48457-48465.

57. Owaki H, Varma R, Gillis B, Bruder JT, Rapp UR, Davis LS, Geppert TD: **Raf-1 is required for T cell IL2 production**. *Embo J* 1993, **12**(11):4367-4373.

58. Koizumi T, Ochi Y, Kobayashi S, Nakanishi M, Tokuhisa T: **Deregulated expression of c-fos disturbs proliferative responses of B cells to sIg cross-linking**. *Cell Immunol* 1994, **155**(2):384-393.

59. Carrozza ML, Jacobs H, Acton D, Verma I, Berns A: **Overexpression of the FosB2 gene in thymocytes causes aberrant development of T cells and thymic epithelial cells**. *Oncogene* 1997, **14**(9):1083-1091.

60. Petrak D, Memon SA, Birrer MJ, Ashwell JD, Zacharchuk CM: **Dominant negative mutant of c-Jun inhibits NF-AT transcriptional activity and prevents IL-2 gene transcription**. *J Immunol* 1994, **153**(5):2046-2051.

61. Hartenstein B, Teurich S, Hess J, Schenkel J, Schorpp-Kistner M, Angel P: **Th2 cell-specific cytokine expression and allergen-induced airway inflammation depend on JunB**. *Embo J* 2002, **21**(23):6321-6329.

62. Meixner A, Karreth F, Kenner L, Wagner EF: **JunD regulates lymphocyte proliferation and T helper cell cytokine expression**. *Embo J* 2004, **23**(6):1325-1335.

63. Sato S, Sanjo H, Takeda K, Ninomiya-Tsuji J, Yamamoto M, Kawai T, Matsumoto K, Takeuchi O, Akira S: **Essential function for the kinase TAK1 in innate and adaptive immune responses**. *Nat Immunol* 2005, **6**(11):1087-1095.

64. Chi H, Lu B, Takekawa M, Davis RJ, Flavell RA: **GADD45beta/GADD45gamma and MEKK4 comprise a genetic pathway mediating STAT4-independent IFNgamma production in T cells**. *Embo J* 2004, **23**(7):1576-1586.

65. Dong C, Yang DD, Wysk M, Whitmarsh AJ, Davis RJ, Flavell RA: **Defective T cell differentiation in the absence of Jnk1**. *Science* 1998, **282**(5396):2092-2095.

66. Yang DD, Conze D, Whitmarsh AJ, Barrett T, Davis RJ, Rincon M, Flavell RA: **Differentiation of CD4+ T cells to Th1 cells requires MAP kinase JNK2**. *Immunity* 1998, **9**(4):575-585.

67. Fischer AM, Katayama CD, Pages G, Pouyssegur J, Hedrick SM: **The role of erk1 and erk2 in multiple stages of T cell development**. *Immunity* 2005, **23**(4):431-443.

68. Pages G, Guerin S, Grall D, Bonino F, Smith A, Anjuere F, Auberger P, Pouyssegur J: **Defective thymocyte maturation in p44 MAP kinase (Erk 1) knockout mice**. *Science* 1999, **286**(5443):1374-1377.

69. Tanaka N, Kamanaka M, Enslen H, Dong C, Wysk M, Davis RJ, Flavell RA: **Differential involvement of p38 mitogen-activated protein kinase kinases MKK3 and MKK6 in T-cell apoptosis**. *EMBO Rep* 2002, **3**(8):785-791.

70. Alberola-Ila J, Hogquist KA, Swan KA, Bevan MJ, Perlmutter RM: **Positive and negative selection invoke distinct signaling pathways**. *J Exp Med* 1996, **184**(1):9-18.

71. Dong C, Yang DD, Tournier C, Whitmarsh AJ, Xu J, Davis RJ, Flavell RA: **JNK is required for effector T-cell function but not for T-cell activation**. *Nature* 2000, **405**(6782):91-94.

72. Nishina H, Fischer KD, Radvanyi L, Shahinian A, Hakem R, Rubie EA, Bernstein A, Mak TW, Woodgett JR, Penninger JM: **Stress-signalling kinase Sek1 protects thymocytes from apoptosis mediated by CD95 and CD3**. *Nature* 1997, **385**(6614):350-353.

73. Nishina H, Bachmann M, Oliveira-dos-Santos AJ, Kozieradzki I, Fischer KD, Odermatt B, Wakeham A, Shahinian A, Takimoto H, Bernstein A *et al*: **Impaired CD28-mediated interleukin 2 production and proliferation in stress kinase SAPK/ERK1 kinase (SEK1)/mitogen-activated protein kinase kinase 4 (MKK4)-deficient T lymphocytes**. *J Exp Med* 1997, **186**(6):941-953.

74. Gomez M, Kioussis D, Cantrell DA: **The GTPase Rac-1 controls cell fate in the thymus by diverting thymocytes from positive to negative selection**. *Immunity* 2001, **15**(5):703-713.

75. Walmsley MJ, Ooi SK, Reynolds LF, Smith SH, Ruf S, Mathiot A, Vanes L, Williams DA, Cancro MP, Tybulewicz VL: **Critical roles for Rac1 and Rac2 GTPases in B cell development and signaling**. *Science* 2003, **302**(5644):459-462.

76. Tskvitaria-Fuller I, Seth A, Mistry N, Gu H, Rosen MK, Wulfing C: **Specific patterns of Cdc42 activity are related to distinct elements of T cell polarization**. *J Immunol* 2006, **177**(3):1708-1720.

77. Duchniewicz M, Zemojtel T, Kolanczyk M, Grossmann S, Scheele JS, Zwartkruis FJ: **Rap1A-deficient T and B cells show impaired integrin-mediated cell adhesion**. *Mol Cell Biol* 2006, **26**(2):643-653.

78. Fischer KD, Zmuldzinas A, Gardner S, Barbacid M, Bernstein A, Guidos C: **Defective T-cell receptor signalling and positive selection of Vav-deficient CD4+ CD8+ thymocytes**. *Nature* 1995, **374**(6521):474-477.

79. Doody GM, Bell SE, Vigorito E, Clayton E, McAdam S, Tooze R, Fernandez C, Lee IJ, Turner M: **Signal transduction through Vav-2 participates in humoral immune responses and B cell maturation**. *Nat Immunol* 2001, **2**(6):542-547.

80. Zhang L, Camerini V, Bender TP, Ravichandran KS: **A nonredundant role for the adapter protein Shc in thymic T cell development**. *Nat Immunol* 2002, **3**(8):749-755.

81. Itoh S, Itoh M, Nishida K, Yamasaki S, Yoshida Y, Narimatsu M, Park SJ, Hibi M, Ishihara K, Hirano T: **Adapter molecule Grb2-associated binder 1 is specifically expressed in marginal zone B cells and negatively regulates thymus-independent antigen-2 responses**. *J Immunol* 2002, **168**(10):5110-5116.

82. Yamasaki S, Nishida K, Hibi M, Sakuma M, Shiina R, Takeuchi A, Ohnishi H, Hirano T, Saito T: **Docking protein Gab2 is phosphorylated by ZAP-70 and negatively regulates T cell receptor signaling by recruitment of inhibitory molecules**. *J Biol Chem* 2001, **276**(48):45175-45183.
