# Supplementary material for: Expansion of signaling genes for adaptive immune system evolution in early vertebrates
Source: BMC Genomics. 2008 May 14;9:218. doi: 10.1186/1471-2164-9-218 (PMC2391169; doi:10.1186/1471-2164-9-218)
Supplement: Additional file 3 — AIS subfamilies and Ensembl IDs. Hs, Homo sapiens; Dm, Drosophila melanogaster; AIS, adaptive immune system. [file 1471-2164-9-218-S3.doc]

| **Additional file 3. AIS subfamilies and Ensembl IDs** | | | |  |
| --- | --- | --- | --- | --- |
|  |  |  | Ensembl ID |  |
| AIS subfamily | Species | Member | Gene ID | Peptide ID |
| JAK | Hs | *JAK1* | ENSG00000162434 | ENSP00000343204 |
|  | Hs | *JAK2* | ENSG00000096968 | ENSP00000371067 |
|  | Hs | *JAK3* | ENSG00000105639 | ENSP00000222246 |
|  | Dm | *hop* | CG1594 | CG1594-PA |
| PIAS | Hs | *PIAS1* | ENSG00000033800 | ENSP00000249636 |
|  | Hs | *PIAS2* | ENSG00000078043 | ENSP00000262161 |
|  | Hs | *PIAS3* | ENSG00000131788 | ENSP00000358305 |
|  | Hs | *PIAS4* | ENSG00000105229 | ENSP00000262971 |
|  | Dm | *Su(var)2-10* | CG8068 | CG8068-PI |
| STAT | Hs | *STAT5A* | ENSG00000126561 | ENSP00000341208 |
|  | Hs | *STAT5B* | ENSG00000173757 | ENSP00000293328 |
|  | Hs | *STAT6* | ENSG00000166888 | ENSP00000300134 |
|  | Dm | *Stat92E* | CG4257 | CG4257-PE |
| SOCS | Hs | *SOCS4* | ENSG00000180008 | ENSP00000341327 |
|  | Hs | *SOCS5* | ENSG00000171150 | ENSP00000305133 |
|  | Dm | *Socs36E* | CG15154 | CG15154-PA |
| SHP | Hs | *PTPN11* | ENSG00000179295 | ENSP00000340944 |
|  | Hs | *PTPN6* | ENSG00000111679 | ENSP00000326010 |
|  | Dm | *csw* | CG3954 | CG3954-PA |
| PRKAR | Hs | *PRKAR1A* | ENSG00000108946 | ENSP00000351410 |
|  | Hs | *PRKAR1B* | ENSG00000188191 | ENSP00000353415 |
|  | Dm | *Pka-R1* | CG3263 | CG3263-PC |
| GNG | Hs | *GNG12* | ENSG00000172380 | ENSP00000360021 |
|  | Hs | *GNG2* | ENSG00000186469 | ENSP00000334448 |
|  | Hs | *GNG3* | ENSG00000162188 | ENSP00000294117 |
|  | Hs | *GNG4* | ENSG00000168243 | ENSP00000355557 |
|  | Hs | *GNG5* | ENSG00000174021 | ENSP00000359679 |
|  | Hs | *GNG8* | ENSG00000167414 | ENSP00000300873 |
|  | Dm | *Ggamma1* | CG8261 | CG8261-PA |
| GNB | Hs | *GNB1* | ENSG00000078369 | ENSP00000367872 |
|  | Hs | *GNB2* | ENSG00000172354 | ENSP00000305260 |
|  | Hs | *GNB3* | ENSG00000111664 | ENSP00000229264 |
|  | Hs | *GNB4* | ENSG00000114450 | ENSP00000232564 |
|  | Dm | *Gbeta13F* | CG10545 | CG10545-PA |
| GNA | Hs | *GNAI1* | ENSG00000127955 | ENSP00000369601 |
|  | Hs | *GNAI2* | ENSG00000114353 | ENSP00000266027 |
|  | Hs | *GNAI3* | ENSG00000065135 | ENSP00000358867 |
|  | Dm | *G-ialpha65A* | CG10060 | CG10060-PA |
| RHO | Hs | *RHOA* | ENSG00000067560 | ENSP00000265538 |
|  | Hs | *RHOC* | ENSG00000155366 | ENSP00000358656 |
|  | Dm | *Rho1* | CG8416 | CG8416-PB |
| DGK | Hs | *DGKA* | ENSG00000065357 | ENSP00000328405 |
|  | Hs | *DGKB* | ENSG00000136267 | ENSP00000258767 |
|  | Hs | *DGKG* | ENSG00000058866 | ENSP00000265022 |
|  | Dm | *Dgk* | CG18654 | CG18654-PB |
| PLCG | Hs | *PLCG1* | ENSG00000124181 | ENSP00000362369 |
|  | Hs | *PLCG2* | ENSG00000197943 | ENSP00000352336 |
|  | Dm | *sl* | CG4200 | CG4200-PA |
| aPKC | Hs | *PRKCI* | ENSG00000163558 | ENSP00000295797 |
|  | Hs | *PRKCZ* | ENSG00000067606 | ENSP00000367830 |
|  | Dm | *aPKC* | CG10261 | CG10261-PA |
| nPKC | Hs | *PRKCD* | ENSG00000163932 | ENSP00000331602 |
|  | Hs | *PRKCQ* | ENSG00000065675 | ENSP00000263125 |
|  | Dm | *Pkcdelta* | CG10524 | CG10524-PA |
| cPKC | Hs | *PRKCA* | ENSG00000154229 | ENSP00000284384 |
|  | Hs | *PRKCB1* | ENSG00000166501 | ENSP00000318315 |
|  | Hs | *PRKCG* | ENSG00000126583 | ENSP00000263431 |
|  | Dm | *Pkc53E* | CG6622 | CG6622-PB |
| CAMK2 | Hs | *CAMK2A* | ENSG00000070808 | ENSP00000261793 |
|  | Hs | *CAMK2B* | ENSG00000058404 | ENSP00000258682 |
|  | Hs | *CAMK2D* | ENSG00000145349 | ENSP00000296402 |
|  | Hs | *CAMK2G* | ENSG00000148660 | ENSP00000277853 |
|  | Dm | *CaMKII* | CG18069 | CG18069-PD |
| CALNA | Hs | *PPP3CA* | ENSG00000138814 | ENSP00000320580 |
|  | Hs | *PPP3CB* | ENSG00000107758 | ENSP00000353881 |
|  | Dm | *CanA-14F* | CG9819 | CG9819-PA |
|  | Dm | *Pp2B-14D* | CG9842 | CG9842-PA |
| CALNB | Hs | *PPP3R1* | ENSG00000115953 | ENSP00000367201 |
|  | Dm | CG14353 | CG14353 | CG14353-PA |
| NFAT | Hs | *NFAT5* | ENSG00000102908 | ENSP00000338806 |
|  | Dm | *NFAT* | CG11172 | CG11172-PA |
| IKBK | Hs | *CHUK* | ENSG00000107566 | ENSP00000359424 |
|  | Hs | *IKBKB* | ENSG00000104365 | ENSP00000339151 |
|  | Dm | *ird5* | CG4201 | CG4201-PA |
| NFKB | Hs | *NFKB1* | ENSG00000109320 | ENSP00000226574 |
|  | Hs | *NFKB2* | ENSG00000077150 | ENSP00000337001 |
|  | Hs | *REL* | ENSG00000162924 | ENSP00000295025 |
|  | Hs | *RELA* | ENSG00000173039 | ENSP00000310229 |
|  | Hs | *RELB* | ENSG00000104856 | ENSP00000221452 |
|  | Dm | *dl* | CG6667 | CG6667-PA |
|  | Dm | *Dif* | CG6794 | CG6794-PA |
| NFKBI | Hs | *BCL3* | ENSG00000069399 | ENSP00000164227 |
|  | Hs | *NFKBIA* | ENSG00000100906 | ENSP00000216797 |
|  | Hs | *NFKBIB* | ENSG00000104825 | ENSP00000312988 |
|  | Hs | *NFKBIE* | ENSG00000146232 | ENSP00000275015 |
|  | Dm | *cact* | CG5848 | CG5848-PA |
| PIK3C | Hs | *PIK3CA* | ENSG00000121879 | ENSP00000263967 |
|  | Hs | *PIK3CB* | ENSG00000051382 | ENSP00000289153 |
|  | Hs | *PIK3CD* | ENSG00000171608 | ENSP00000366563 |
|  | Dm | *Pi3K92E* | CG4141 | CG4141-PA |
| PIK3R | Hs | *PIK3R1* | ENSG00000145675 | ENSP00000274335 |
|  | Hs | *PIK3R2* | ENSG00000105647 | ENSP00000222254 |
|  | Hs | *PIK3R3* | ENSG00000117461 | ENSP00000361075 |
|  | Dm | *Pi3K21B* | CG2699 | CG2699-PA |
| PTEN | Hs | *PTEN* | ENSG00000171862 | ENSP00000361021 |
|  | Dm | *Pten* | CG5671 | CG5671-PA |
| AKT | Hs | *AKT1* | ENSG00000142208 | ENSP00000270202 |
|  | Hs | *AKT2* | ENSG00000105221 | ENSP00000309428 |
|  | Hs | *AKT3* | ENSG00000117020 | ENSP00000336943 |
|  | Dm | *Akt1* | CG4006 | CG4006-PA |
| SRC | Hs | *CSK* | ENSG00000103653 | ENSP00000220003 |
|  | Hs | *MATK* | ENSG00000007264 | ENSP00000313859 |
|  | Dm | *csk* | CG17309 | CG17309-PB |
| ABL | Hs | *ABL1* | ENSG00000097007 | ENSP00000361423 |
|  | Hs | *ABL2* | ENSG00000143322 | ENSP00000356595 |
|  | Dm | *Abl* | CG4032 | CG4032-PA |
| TEC | Hs | *BMX* | ENSG00000102010 | ENSP00000369754 |
|  | Hs | *BTK* | ENSG00000010671 | ENSP00000308176 |
|  | Hs | *ITK* | ENSG00000113263 | ENSP00000231189 |
|  | Hs | *TEC* | ENSG00000135605 | ENSP00000370912 |
|  | Hs | *TXK* | ENSG00000074966 | ENSP00000370926 |
|  | Dm | *Btk29A* | CG8049 | CG8049-PB |
| GRB2 | Hs | *GRAP* | ENSG00000154016 | ENSP00000284154 |
|  | Hs | *GRB2* | ENSG00000177885 | ENSP00000339007 |
|  | Dm | *drk* | CG6033 | CG6033-PA |
| BLNK | Hs | *BLNK* | ENSG00000095585 | ENSP00000224337 |
|  | Dm | CG15529 | CG15529 | CG15529-PA |
| SOS | Hs | *SOS1* | ENSG00000115904 | ENSP00000263879 |
|  | Hs | *SOS2* | ENSG00000100485 | ENSP00000346183 |
|  | Dm | *Sos* | CG7793 | CG7793-PA |
| RAS | Hs | *HRAS* | ENSG00000174775 | ENSP00000312305 |
|  | Hs | *KRAS* | ENSG00000133703 | ENSP00000256078 |
|  | Dm | *Ras85D* | CG9375 | CG9375-PA |
| RAF | Hs | *ARAF* | ENSG00000078061 | ENSP00000366244 |
|  | Hs | *BRAF* | ENSG00000157764 | ENSP00000288602 |
|  | Hs | *RAF1* | ENSG00000132155 | ENSP00000251849 |
|  | Dm | *phl* | CG2845 | CG2845-PA |
| FOS | Hs | *FOS* | ENSG00000170345 | ENSP00000306245 |
|  | Hs | *FOSB* | ENSG00000125740 | ENSP00000245919 |
|  | Hs | *FOSL2* | ENSG00000075426 | ENSP00000368939 |
|  | Dm | *kay* | CG33956 | CG33956-PD |
| JUN | Hs | *JUN* | ENSG00000177606 | ENSP00000360266 |
|  | Hs | *JUNB* | ENSG00000171223 | ENSP00000303315 |
|  | Hs | *JUND* | ENSG00000130522 | ENSP00000252818 |
|  | Dm | *Jra* | CG2275 | CG2275-PB |
| MAP3K-1 | Hs | *MAP3K7* | ENSG00000135341 | ENSP00000358335 |
|  | Dm | *Tak1* | CG18492 | CG18492-PA |
| MAP3K-2 | Hs | *MAP3K4* | ENSG00000085511 | ENSP00000297332 |
|  | Dm | *Mekk1* | CG7717 | CG7717-PA |
| JNK | Hs | *MAPK10* | ENSG00000109339 | ENSP00000309857 |
|  | Hs | *MAPK8* | ENSG00000107643 | ENSP00000363304 |
|  | Hs | *MAPK9* | ENSG00000050748 | ENSP00000321410 |
|  | Dm | *bsk* | CG5680 | CG5680-PB |
| cMAPK | Hs | *MAPK1* | ENSG00000100030 | ENSP00000215832 |
|  | Hs | *MAPK3* | ENSG00000102882 | ENSP00000263025 |
|  | Dm | *rl* | CG12559 | CG12559-PC |
| MAP2K-1 | Hs | *MAP2K3* | ENSG00000034152 | ENSP00000345083 |
|  | Hs | *MAP2K6* | ENSG00000108984 | ENSP00000351997 |
|  | Dm | *lic* | CG12244 | CG12244-PA |
| MAP2K-2 | Hs | *MAP2K1* | ENSG00000169032 | ENSP00000302486 |
|  | Hs | *MAP2K2* | ENSG00000126934 | ENSP00000262948 |
|  | Dm | *Dsor1* | CG15793 | CG15793-PA |
| MAP2K-3 | Hs | *MAP2K7* | ENSG00000076984 | ENSP00000314228 |
|  | Dm | *hep* | CG4353 | CG4353-PA |
| MAP2K-4 | Hs | *MAP2K4* | ENSG00000065559 | ENSP00000262445 |
|  | Dm | *Mkk4* | CG9738 | CG9738-PA |
| RAC | Hs | ENSG00000172895 | ENSG00000172895 | ENSP00000309219 |
|  | Hs | *RAC1* | ENSG00000136238 | ENSP00000348461 |
|  | Hs | *RAC2* | ENSG00000128340 | ENSP00000249071 |
|  | Hs | *RAC3* | ENSG00000169750 | ENSP00000304283 |
|  | Dm | *Rac1* | CG2248 | CG2248-PA |
|  | Dm | *Rac2* | CG8556 | CG8556-PA |
| CDC42 | Hs | *CDC42* | ENSG00000070831 | ENSP00000337669 |
|  | Hs | ENSG00000152994 | ENSG00000152994 | ENSP00000282947 |
|  | Dm | *Cdc42* | CG12530 | CG12530-PA |
| RAP1 | Hs | ENSG00000176276 | ENSG00000176276 | ENSP00000345280 |
|  | Hs | *RAP1A* | ENSG00000116473 | ENSP00000348786 |
|  | Hs | *RAP1B* | ENSG00000127314 | ENSP00000250559 |
|  | Dm | *R* | CG1956 | CG1956-PA |
| VAV | Hs | *VAV1* | ENSG00000141968 | ENSP00000302269 |
|  | Hs | *VAV2* | ENSG00000160293 | ENSP00000360917 |
|  | Hs | *VAV3* | ENSG00000134215 | ENSP00000359073 |
|  | Dm | *vav* | CG7893 | CG7893-PB |
| SHC | Hs | *SHC1* | ENSG00000160691 | ENSP00000357438 |
|  | Hs | *SHC2* | ENSG00000129946 | ENSP00000264554 |
|  | Hs | *SHC3* | ENSG00000148082 | ENSP00000364995 |
|  | Hs | *SHC4* | ENSG00000185634 | ENSP00000329668 |
|  | Dm | *Shc* | CG3715 | CG3715-PA |
| GAB | Hs | *GAB1* | ENSG00000109458 | ENSP00000262995 |
|  | Hs | *GAB2* | ENSG00000033327 | ENSP00000302452 |
|  | Hs | *GAB3* | ENSG00000160219 | ENSP00000358588 |
|  | Dm | *dos* | CG1044 | CG1044-PA |
| Hs, *Homo sapiens*; Dm, *Drosophila melanogaster*; AIS, adaptive immune system. | | | | |
