# Supplementary material for: Expansion of signaling genes for adaptive immune system evolution in early vertebrates
Source: BMC Genomics. 2008 May 14;9:218. doi: 10.1186/1471-2164-9-218 (PMC2391169; doi:10.1186/1471-2164-9-218)
Supplement: Additional file 4 — Phylogenetic trees of AIS subfamilies. AIS, adaptive immune system. The name of each AIS subfamily is in boldface, and each operational taxonomic unit (OTU) is labeled with its Ensembl ID by using the following species abbreviations: Dm, Drosophila melanogaster; Ol, Oryzias latipes; Hs, Homo sapiens; Mm, Mus musculus. Support values found by TREE-PUZZLE are at interior nodes. The scale bar indicates substitutions per site. The consensus tree for each AIS subfamily was drawn by using NJplot. [file 1471-2164-9-218-S4.doc]

**Additional file 4. Phylogenetic trees of AIS subfamilies**

**JAK**

**PIAS**

**STAT**

**SOCS**

**SHP**

**PRKAR**

**GNG**

no phylogenetic tree

**GNB**

**GNA**

**RHO**

**DGK**

**PLCG**

**aPKC**

**nPKC**

**cPKC**

**CAMK2**

**CALNA**

**CALNB**

no phylogenetic tree

**NFAT**

**IKBK**

**NFKB**

**NFKBI**

**PIK3C**

**PIK3R**

**PTEN**

**AKT**

**SRC**

**ABL**

**TEC**

**GRB2**

**BLNK**

**SOS**

**RAS**

**RAF**

**FOS**

**JUN**

**MAP3K-1**

**MAP3K-2**

**JNK**

**cMAPK**

**MAP2K-1**

**MAP2K-2**

**MAP2K-3**

**MAP2K-4**

**RAC**

**CDC42**

**RAP1**

**VAV**

**SHC**

**GAB**

AIS, adaptive immune system. The name of each AIS subfamily is in boldface, and each OUT (operational taxonomic unit) is labeled with its Ensembl ID by using the following species abbreviations: Dm, *Drosophila melanogaster*; Ol, *Oryzias latipes*; Hs, *Homo sapiens*; Mm, *Mus musculus*. Support values found by TREE-PUZZLE [1] are at interior nodes. The scale bar indicates substitutions per site. The consensus tree for each AIS subfamily was drawn by using NJplot [2] .

**Supplementary References**

1. Schmidt HA, Strimmer K, Vingron M, von Haeseler A: **TREE-PUZZLE: maximum likelihood phylogenetic analysis using quartets and parallel computing**. *Bioinformatics* 2002, **18**(3):502-504.

2. Perriere G, Gouy M: **WWW-query: an on-line retrieval system for biological sequence banks**. *Biochimie* 1996, **78**(5):364-369.
