# Supplementary material for: Expansion of signaling genes for adaptive immune system evolution in early vertebrates
Source: BMC Genomics. 2008 May 14;9:218. doi: 10.1186/1471-2164-9-218 (PMC2391169; doi:10.1186/1471-2164-9-218)
Supplement: Additional file 6 — Paralogous pairs that are part of paralogons. Hs, Homo sapiens; AIS, adaptive immune system. [file 1471-2164-9-218-S6.doc]

| **Additional file 6. Paralogous pairs that are part of paralogons** | | | |
| --- | --- | --- | --- |
| AIS subfamily | Number of Hs members | Number of paralogous pairs | Number of paralogous pairs that are part of paralogons |
| JAK | 3 | 3 | 3 |
| PIAS | 4 | 6 | 4 |
| STAT | 3 | 3 | 3 |
| SOCS | 2 | 1 | 1 |
| SHP | 2 | 1 | 0 |
| PRKAR | 2 | 1 | 1 |
| GNG | 6 | 15 | 5 |
| GNB | 4 | 6 | 4 |
| GNA | 3 | 3 | 2 |
| RHO | 2 | 1 | 1 |
| DGK | 3 | 3 | 2 |
| PLCG | 2 | 1 | 1 |
| aPKC | 2 | 1 | 1 |
| nPKC | 2 | 1 | 1 |
| cPKC | 3 | 3 | 2 |
| CAMK2 | 4 | 6 | 4 |
| CALNA | 2 | 1 | 1 |
| CALNB | 1 | 0 | 0 |
| NFAT | 1 | 0 | 0 |
| IKBK | 2 | 1 | 1 |
| NFKB | 5 | 10 | 5 |
| NFKBI | 4 | 6 | 2 |
| PIK3C | 3 | 3 | 3 |
| PIK3R | 3 | 3 | 3 |
| PTEN | 1 | 0 | 0 |
| AKT | 3 | 3 | 2 |
| SRC | 2 | 1 | 0 |
| ABL | 2 | 1 | 1 |
| TEC | 5 | 10 | 7 |
| GRB2 | 2 | 1 | 1 |
| BLNK | 1 | 0 | 0 |
| SOS | 2 | 1 | 1 |
| RAS | 2 | 1 | 0 |
| RAF | 3 | 3 | 2 |
| FOS | 3 | 3 | 1 |
| JUN | 3 | 3 | 3 |
| MAP3K-1 | 1 | 0 | 0 |
| MAP3K-2 | 1 | 0 | 0 |
| JNK | 3 | 3 | 3 |
| cMAPK | 2 | 1 | 1 |
| MAP2K-1 | 2 | 1 | 1 |
| MAP2K-2 | 2 | 1 | 1 |
| MAP2K-3 | 1 | 0 | 0 |
| MAP2K-4 | 1 | 0 | 0 |
| RAC | 4 | 6 | 3 |
| CDC42 | 2 | 1 | 0 |
| RAP1 | 3 | 3 | 2 |
| VAV | 3 | 3 | 3 |
| SHC | 4 | 6 | 6 |
| GAB | 3 | 3 | 2 |
| Total | 129 | 134 | 90 |
| Hs, *Homo sapiens*; AIS, adaptive immune system. | | | |
