# Supplementary material for: Expansion of signaling genes for adaptive immune system evolution in early vertebrates
Source: BMC Genomics. 2008 May 14;9:218. doi: 10.1186/1471-2164-9-218 (PMC2391169; doi:10.1186/1471-2164-9-218)
Supplement: Additional file 7 — Genomic maps of all AIS subfamilies. AIS, adaptive immune system. The chromosomes of the human genome from chromosome 1 (chr1) to chromosomes X and Y (chrX, Y) are arranged in a circle (blue line). Red lines connect the genomic map positions of the human genes in an AIS subfamily. A gray line connects the genomic map positions of the genes in each BV paralogous pair (a paralogous pair that were formed at the Base of the Vertebrate lineage) of paralogons. [file 1471-2164-9-218-S7.doc]

**Additional file 7. Genomic maps of all AIS subfamilies**

AIS, adaptive immune system. The chromosomes of the human genome from chromosome 1 (chr1) to chromosomes X and Y (chrX, Y) are arranged in a circle (blue line). Red lines connect the genomic map positions of the human genes in an AIS subfamily. A gray line connects the genomic map positions of the genes in each BV paralogous pair (a paralogous pair that were formed at the Base of the Vertebrate lineage) of paralogons.
