# Supplementary material for: Expansion of signaling genes for adaptive immune system evolution in early vertebrates
Source: BMC Genomics. 2008 May 14;9:218. doi: 10.1186/1471-2164-9-218 (PMC2391169; doi:10.1186/1471-2164-9-218)
Supplement: Additional file 8 — Tissue/organ classification scheme. [file 1471-2164-9-218-S8.doc]

| **Additional file 8. Tissue/organ classification scheme** | |
| --- | --- |
| System-level function category | Tissue/organ name in the microarray data |
| Bone marrow | Bone marrow |
|  | BM-CD34+ |
|  | BM-CD105+endothelial |
|  | BM-CD71+early erythroid |
|  | BM-CD33+myeloid |
| Muscle tissue | Skeletal muscle |
|  | Smooth muscle |
|  | Cardiac myocytes |
| Reproductive organ | Uterus |
|  | Uterus corpus |
|  | Ovary |
|  | Prostate |
|  | Testis |
|  | Testis seminiferous tubule |
|  | Testis germ cell |
|  | Testis interstitial |
|  | Testis Leydig Cell |
| Respiration organ | Lung |
|  | Fetal lung |
|  | Trachea |
|  | Bronchial epithelial cells |
| Epithelium | Salivary gland |
|  | Tongue |
|  | Tonsil |
|  | Skin |
| Internal organ and metabolism system | Pituitary |
|  | Placenta |
|  | Adrenal gland |
|  | Adrenal cortex |
|  | Pancreas |
|  | Pancreatic islets |
|  | Thyroid |
|  | Fetal thyroid |
|  | Liver |
|  | Fetal liver |
|  | Appendix |
|  | Kidney |
|  | Atrioventricular node |
|  | Heart |
|  | Adipocyte |
| Adaptive immune system | 721 B lymphoblasts |
|  | Lymph node |
|  | Thymus |
|  | PB-CD19+B cells |
|  | PB-CD4+T cells |
|  | PB-CD8+T cells |
| Cancer | Leukemia chronic myelogenous (k562) |
|  | Colorectal adenocarcinoma |
|  | Leukemia lymphoblastic (molt4) |
|  | Leukemia promyelocytic (hl60) |
|  | Lymphomaburkitts Daudi |
|  | Lymphomaburkitts Raji |
| Blood | Whole blood |
| Innate immunity | PB-CD56+NK cells |
|  | PB-CD14+monocytes |
|  | PB-BDCA4+dentritic cells |
| Nervous system | Spinal cord |
|  | Ciliary ganglion |
|  | Superior Cervical Ganglion |
|  | DRG |
|  | Whole brain |
|  | Fetal brain |
|  | Amygdala |
|  | Cerebellum |
|  | Cerebellum peduncles |
|  | Cingulate cortex |
|  | Hypothalamus |
|  | Medulla oblongata |
|  | Occipital lobe |
|  | Olfactory bulb |
|  | Parietal lobe |
|  | Pons |
|  | Prefrontal cortex |
|  | Temporal lobe |
|  | Thalamus |
|  | Subthalamic nucleus |
|  | Caudate nucleus |
|  | Globus pallidus |
|  | Trigeminal ganglion |
