# Supplementary material for: Expansion of signaling genes for adaptive immune system evolution in early vertebrates
Source: BMC Genomics. 2008 May 14;9:218. doi: 10.1186/1471-2164-9-218 (PMC2391169; doi:10.1186/1471-2164-9-218)
Supplement: Additional file 9 — System-level function category profile of AIS subfamilies. * P values are calculated by using hypergeometric distribution (before Bonferroni correction). BM, bone marrow; MT, muscle tissue; RepO, reproductive organ; ResO, respiration organ; Epi, epithelium; IO, internal organ and metabolism system; AIS, adaptive immune system; II, innate immunity; NS, Nerve system. System-level function category C is assigned to subfamily F if and only if F is expressed in at least one tissue or organ that is classified into C. "+" indicates that the system-level function category is assigned to the subfamily; otherwise "-" is indicated. [file 1471-2164-9-218-S9.doc]

| **Additional file 9. System-level function category profile of AIS subfamilies** | | | | | | | | | |  |  |
| --- | --- | --- | --- | --- | --- | --- | --- | --- | --- | --- | --- |
|  | System-level function category | | | |  |  |  |  |  |  |  |
| AIS subfamily | BM | MT | RepO | ResO | Epi | Cancer | IO | AIS | Blood | II | NS |
| JAK | - | - | + | - | - | - | - | + | - | + | - |
| PIAS | - | + | + | - | - | - | - | - | - | - | + |
| STAT | + | + | + | - | - | + | + | - | + | + | + |
| SOCS | - | - | + | - | - | - | - | - | - | - | + |
| SHP | - | - | - | - | - | - | + | + | + | + | + |
| PRKAR | - | - | + | - | - | - | + | - | - | - | + |
| GNG | - | + | + | - | - | - | + | + | - | - | + |
| GNB | - | - | - | - | - | - | - | + | - | + | + |
| GNA | + | + | - | + | - | - | - | + | + | + | + |
| RHO | + | + | - | + | - | + | + | + | - | + | - |
| DGK | - | - | - | - | - | - | + | + | - | - | + |
| PLCG | - | - | - | - | - | + | - | + | - | - | + |
| aPKC | - | - | - | - | - | - | - | - | - | - | + |
| nPKC | + | - | - | - | - | - | + | - | + | + | + |
| cPKC | - | - | - | - | - | - | - | - | - | - | + |
| CAMK2 | - | - | - | - | - | - | - | - | - | - | + |
| CALNA | - | - | - | - | - | - | - | - | - | - | + |
| CALNB | no members with any probes | | |  |  |  |  |  |  |  |  |
| NFAT | - | - | - | - | - | - | + | - | - | - | + |
| IKBK | + | - | - | - | - | - | - | + | - | + | + |
| NFKB | + | - | + | - | - | + | + | + | - | - | + |
| NFKBI | + | + | + | + | - | + | - | + | + | + | - |
| PIK3C | - | + | - | - | - | + | - | + | + | + | + |
| PIK3R | - | - | + | - | - | - | + | - | - | - | + |
| PTEN | - | - | + | - | - | - | - | + | + | - | - |
| AKT | - | + | + | + | - | + | - | - | - | - | + |
| SRC | - | - | - | - | - | + | - | + | - | + | - |
| ABL | - | - | + | - | - | + | + | - | - | - | + |
| TEC | + | - | - | - | - | + | + | + | - | + | + |
| GRB2 | - | + | - | - | - | - | + | - | - | - | - |
| BLNK | - | - | - | - | + | - | - | + | - | + | - |
| SOS | - | - | + | - | - | - | + | + | + | - | + |
| RAS | - | + | - | - | - | + | + | - | - | - | + |
| RAF | - | - | + | - | - | + | + | - | + | - | + |
| FOS | + | + | + | - | - | - | + | - | - | + | + |
| JUN | + | - | + | + | - | - | + | - | - | + | - |
| MAP3K-1 | - | - | - | - | - | - | - | - | - | - | + |
| MAP3K-2 | - | - | - | - | - | - | - | + | - | - | - |
| JNK | - | - | - | - | - | + | - | - | - | - | + |
| cMAPK | - | - | - | - | - | - | - | - | - | - | + |
| MAP2K-1 | + | + | - | - | - | - | + | - | - | - | + |
| MAP2K-2 | + | - | + | - | - | + | - | - | - | - | + |
| MAP2K-3 | - | + | - | - | - | - | + | - | - | - | + |
| MAP2K-4 | no members with any probes | | |  |  |  |  |  |  |  |  |
| RAC | + | - | - | - | - | + | - | + | + | + | + |
| CDC42 | + | - | - | - | - | - | - | - | + | + | - |
| RAP1 | - | - | - | - | - | - | - | - | + | + | - |
| VAV | - | - | - | - | + | - | + | - | - | - | + |
| SHC | - | + | - | + | - | + | - | - | - | - | + |
| GAB | - | - | - | - | - | - | - | - | + | - | + |
| Total | 14 | 14 | 17 | 6 | 2 | 16 | 21 | 19 | 13 | 18 | 37 |
| *P* value* | 2.64E-2 | 4.52E-4 | 8.27E-2 | 8.42E-2 | 2.77E-1 | 5.44E-2 | 8.71E-2 | 1.51E-3 | 9.46E-7 | 9.04E-8 | 2.21E-3 |
| **P* values are calculated by using hypergeometric distribution (before Bonferroni correction). | | | | | | | | | | | |
| BM, bone marrow; MT, muscle tissue; RepO, reproductive organ; ResO, respiration organ; Epi, epithelium; IO, internal organ and metabolism system; AIS, adaptive immune system; II, innate immunity; NS, nervous system. | | | | | | | | | | | |
| System-level function category *C* is assigned to subfamily *F* if and only if *F* is expressed in at least one tissue or organ that is classified into *C*. “+” indicates that the system-level function category is assigned to the subfamily; otherwise “–” is indicated. | | | | | | | | | | | |
