# Supplementary material for: Expansion of signaling genes for adaptive immune system evolution in early vertebrates
Source: BMC Genomics. 2008 May 14;9:218. doi: 10.1186/1471-2164-9-218 (PMC2391169; doi:10.1186/1471-2164-9-218)
Supplement: Additional file 10 — Assignment of system-level function categories to human and Drosophila genes in AIS subfamilies by using microarray expression data and FlyBase annotations. "+" indicates that a human gene is specifically expressed (Z-score ≧ 2) in at least one tissue or organ that is classified into the listed system-level function category; otherwise, "-" is indicated. For Drosophila genes, anatomic ontology terms are listed according to FlyBase annotation; descriptions of terms are given in parentheses. Dm, Drosophila melanogaster; Hs, Homo sapiens; AIS, adaptive immune system. [file 1471-2164-9-218-S10.doc]

| **Additional file 10. Assignment of system-level function categories to human and *Drosophila* genes in AIS subfamilies by using microarray expression data and FlyBase annotations** | | | | | | |
| --- | --- | --- | --- | --- | --- | --- |
|  |  |  | System-level function category |  |  |  |
| AIS subfamily | Species | Member | Muscle tissue | Blood | Innate immunity | Nervous system |
| JAK | Hs | *JAK1* | - | - | + | - |
|  | Hs | *JAK2* | no probes |  |  |  |
|  | Hs | *JAK3* | - | - | - | - |
|  | Dm | *hop* | - | FBbt:00005064 (melanotic mass), FBbt:00001684 (embryonic/larval hemocyte), FBbt:00001668 (lymph gland), FBbt:00001685 (plasmatocyte), FBbt:00005063 (hemocyte), FBbt:00001687 (lamellocyte), FBbt:00001690 (crystal cell), FBbt:00005701 (larval hemocyte), FBbt:00001688 (podocyte) | FBbt:00005064 (melanotic mass), FBbt:00001684 (embryonic/larval hemocyte), FBbt:00001685 (plasmatocyte), FBbt:00005063 (hemocyte), FBbt:00001687 (lamellocyte), FBbt:00001690 (crystal cell), FBbt:00005701 (larval hemocyte), FBbt:00001688 (podocyte) | FBbt:00004510 (ommatidium), FBbt:00004508 (eye), FBbt:00004509 (eye equator), FBbt:00006009 (eye photoreceptor cell), FBbt:00004133 (interommatidial bristle), FBbt:00005098 (peripheral nervous system), FBbt:00001920 (larval brain) |
| PIAS | Hs | *PIAS1* | - | - | - | - |
|  | Hs | *PIAS2* | + | - | - | - |
|  | Hs | *PIAS3* | - | - | - | - |
|  | Hs | *PIAS4* | - | - | - | + |
|  | Dm | *Su(var)2-10* | - | - | - | FBbt:00004199 (lens), FBbt:00004508 (eye), FBbt:00004510 (ommatidium), FBbt:00004211 (photoreceptor cell) |
| STAT | Hs | *STAT5A* | - | - | - | + |
|  | Hs | *STAT5B* | + | + | - | + |
|  | Hs | *STAT6* | - | + | + | - |
|  | Dm | *Stat92E* | - | - | - | FBbt:00005094 (central nervous system), FBbt:00005103 (commissure), FBbt:00001103 (longitudinal connective), FBbt:00005098 (peripheral nervous system), FBbt:00004508 (eye), FBbt:00004510 (ommatidium) |
| SOCS | Hs | *SOCS4* | no probes |  |  |  |
|  | Hs | *SOCS5* | - | - | - | + |
|  | Dm | *Socs36E* | - | - | - | FBbt:00004230 (pigment cell) |
| SHP | Hs | *PTPN11* | - | - | - | + |
|  | Hs | *PTPN6* | - | + | + | - |
|  | Dm | *csw* | FBbt:00000591 (abdominal dorsal acute muscle 1), FBbt:00000596 (abdominal ventral acute muscle 2) | FBbt:00001667 (embryonic/larval pericardial cell) | - | FBbt:00000113 (neurectoderm), FBbt:00005103 (commissure), FBbt:00001103 (longitudinal connective), FBbt:00004510 (ommatidium), FBbt:00004133 (interommatidial bristle), FBbt:00004217 (photoreceptor cell R3), FBbt:00004219 (photoreceptor cell R4), FBbt:00004225 (photoreceptor cell R7), FBbt:00006009 (eye photoreceptor cell), FBbt:00006007 (outer photoreceptor cell), FBbt:00004508 (eye), FBbt:00004211 (photoreceptor cell), FBbt:00005162 (photoreceptor) |
| PRKAR | Hs | *PRKAR1A* | - | - | - | + |
|  | Hs | *PRKAR1B* | - | - | - | + |
|  | Dm | *Pka-R1* | - | - | - | FBbt:00004312 (scutellar bristle), FBbt:00004246 (leg sensillum) |
| GNG | Hs | *GNG12* | + | - | - | - |
|  | Hs | *GNG2* | no probes |  |  |  |
|  | Hs | *GNG3* | - | - | - | + |
|  | Hs | *GNG4* | - | - | - | + |
|  | Hs | *GNG5* | - | - | - | - |
|  | Hs | *GNG8* | no probes |  |  |  |
|  | Dm | *Ggamma1* | - | FBbt:00005678 (embryonic pericardial cell), FBbt:00001666 (cardioblast) | - | FBbt:00005146 (neuroblast), FBbt:00005149 (ganglion mother cell), FBbt:00001332 (embryonic neuroblast) |
| GNB | Hs | *GNB1* | - | - | + | + |
|  | Hs | *GNB2* | no probes |  |  |  |
|  | Hs | *GNB3* | - | - | - | - |
|  | Hs | *GNB4* | no probes |  |  |  |
|  | Dm | *Gbeta13F* | - | - | - | FBbt:00001332 (embryonic neuroblast), FBbt:00005146 (neuroblast), FBbt:00001572 (EL neuron) |
| GNA | Hs | *GNAI1* | - | - | - | + |
|  | Hs | *GNAI2* | - | - | + | - |
|  | Hs | *GNAI3* | + | + | + | - |
|  | Dm | *G-ialpha65A* | - | - | - | FBbt:00005146 (neuroblast), FBbt:00001454 (RP2sib neuron), FBbt:00001453 (RP2 neuron), FBbt:00005177 (chaeta), FBbt:00001332 (embryonic neuroblast), FBbt:00004331 (medial triple row) |
| RHO | Hs | *RHOA* | - | - | + | - |
|  | Hs | *RHOC* | + | - | - | - |
|  | Dm | *Rho1* | FBbt:00005647 (embryonic muscle system), FBbt:00000482 (ventral longitudinal muscle) | FBbt:00005700 (embryonic hemocyte) | FBbt:00005700 (embryonic hemocyte) | FBbt:00004510 (ommatidium), FBbt:00006009 (eye photoreceptor cell), FBbt:00005801 (mushroom body), FBbt:00001327 (peripheral glial cell), FBbt:00004232 (secondary pigment cell), FBbt:00004233 (tertiary pigment cell), FBbt:00004211 (photoreceptor cell), FBbt:00004508 (eye), FBbt:00004200 (retina), FBbt:00004230 (pigment cell), FBbt:00005164 (external sensory organ precursor cell), FBbt:00001967 (Bolwig's nerve), FBbt:00005407 (dorsal lobe), FBbt:00001455 (RP3 neuron) |
| DGK | Hs | *DGKA* | - | - | - | + |
|  | Hs | *DGKB* | - | - | - | + |
|  | Hs | *DGKG* | - | - | - | + |
|  | Dm | *Dgk* | - | - | - | - |
| PLCG | Hs | *PLCG1* | - | - | - | - |
|  | Hs | *PLCG2* | - | - | - | + |
|  | Dm | *sl* | - | - | - | FBbt:00004510 (ommatidium), FBbt:00004225 (photoreceptor cell R7), FBbt:00004508 (eye), FBbt:00004133 (interommatidial bristle), FBbt:00006009 (eye photoreceptor cell), FBbt:00004193 (cone cell), FBbt:00004231 (primary pigment cell) |
| aPKC | Hs | *PRKCI* | no probes |  |  |  |
|  | Hs | *PRKCZ* | - | - | - | + |
|  | Dm | *aPKC* | - | - | - | FBbt:00004510 (ommatidium), FBbt:00006009 (eye photoreceptor cell), FBbt:00005116 (bouton) |
| nPKC | Hs | *PRKCD* | - | + | + | - |
|  | Hs | *PRKCQ* | - | - | - | + |
|  | Dm | *Pkcdelta* | - | - | - | - |
| cPKC | Hs | *PRKCA* | no probes |  |  |  |
|  | Hs | *PRKCB1* | - | - | - | + |
|  | Hs | *PRKCG* | - | - | - | + |
|  | Dm | *Pkc53E* | - | - | - | - |
| CAMK2 | Hs | *CAMK2A* | no probes |  |  |  |
|  | Hs | *CAMK2B* | - | - | - | + |
|  | Hs | *CAMK2D* | no probes |  |  |  |
|  | Hs | *CAMK2G* | - | - | - | + |
|  | Dm | *CaMKII* | - | - | - | - |
| CALNA | Hs | *PPP3CA* | - | - | - | + |
|  | Hs | *PPP3CB* | - | - | - | + |
|  | Dm | *CanA-14F* | - | - | - | - |
|  | Dm | *Pp2B-14D* | - | - | - | FBbt:00004508 (eye), FBbt:00004510 (ommatidium), FBbt:00004133 (interommatidial bristle), FBbt:00006009 (eye photoreceptor cell), FBbt:00004225 (photoreceptor cell R7), FBbt:00004217 (photoreceptor cell R3), FBbt:00004219 (photoreceptor cell R4) |
| CALNB | Hs | *PPP3R1* | no probes |  |  |  |
|  | Dm | CG14353 | - | - | - | - |
| NFAT | Hs | *NFAT5* | - | - | - | + |
|  | Dm | *NFAT* | - | - | - | FBbt:00001997 (anterior fascicle), FBbt:00004508 (eye), FBbt:00004306 (dorsocentral bristle), FBbt:00004312 (scutellar bristle), FBbt:00005169 (trichogen cell) |
| IKBK | Hs | *CHUK* | - | - | - | + |
|  | Hs | *IKBKB* | - | - | + | - |
|  | Dm | *ird5* | - | - | - | - |
| NFKB | Hs | *NFKB1* | - | - | - | + |
|  | Hs | *NFKB2* | - | - | - | - |
|  | Hs | *REL* | - | - | - | - |
|  | Hs | *RELA* | - | - | - | + |
|  | Hs | *RELB* | - | - | - | - |
|  | Dm | *dl* | - | FBbt:00001685 (plasmatocyte) | FBbt:00001685 (plasmatocyte) | FBbt:00005801 (mushroom body), FBbt:00003686 (Kenyon cell) |
|  | Dm | *Dif* | - | FBbt:00005701 (larval hemocyte), FBbt:00001687 (lamellocyte), FBbt:00001685 (plasmatocyte) | FBbt:00005701 (larval hemocyte), FBbt:00001687 (lamellocyte), FBbt:00001685 (plasmatocyte) | - |
| NFKBI | Hs | *BCL3* | + | + | - | - |
|  | Hs | *NFKBIA* | - | - | + | - |
|  | Hs | *NFKBIB* | no probes |  |  |  |
|  | Hs | *NFKBIE* | - | - | - | - |
|  | Dm | *cact* | - | FBbt:00005064 (melanotic mass), FBbt:00001687 (lamellocyte), FBbt:00001683 (embryonic/larval hemolymph), FBbt:00001668 (lymph gland), FBbt:00005701 (larval hemocyte), FBbt:00001688 (podocyte) | FBbt:00005064 (melanotic mass), FBbt:00001687 (lamellocyte), FBbt:00005701 (larval hemocyte), FBbt:00001688 (podocyte) | FBbt:00001860 (dorsal fold) |
| PIK3C | Hs | *PIK3CA* | - | - | - | - |
|  | Hs | *PIK3CB* | + | - | - | + |
|  | Hs | *PIK3CD* | - | + | + | + |
|  | Dm | *Pi3K92E* | - | - | - | FBbt:00004510 (ommatidium), FBbt:00004508 (eye), FBbt:00005177 (chaeta), FBbt:00004133 (interommatidial bristle) |
| PIK3R | Hs | *PIK3R1* | - | - | - | + |
|  | Hs | *PIK3R2* | - | - | - | + |
|  | Hs | *PIK3R3* | - | - | - | + |
|  | Dm | *Pi3K21B* | - | - | - | - |
| PTEN | Hs | *PTEN* | - | + | - | - |
|  | Dm | *Pten* | - | FBbt:00003154 (adult heart) | - | FBbt:00004508 (eye), FBbt:00004510 (ommatidium), FBbt:00004133 (interommatidial bristle), FBbt:00004211 (photoreceptor cell), FBbt:00001725 (larval corpus allatum), FBbt:00006009 (eye photoreceptor cell), FBbt:00004230 (pigment cell), FBbt:00004199 (lens), FBbt:00004326 (wing margin bristle) |
| AKT | Hs | *AKT1* | - | - | - | - |
|  | Hs | *AKT2* | + | - | - | + |
|  | Hs | *AKT3* | no probes |  |  |  |
|  | Dm | *Akt1* | - | - | - | FBbt:00004510 (ommatidium), FBbt:00004508 (eye), FBbt:00004133 (interommatidial bristle), FBbt:00004326 (wing margin bristle) |
| SRC | Hs | *CSK* | - | - | + | - |
|  | Hs | *MATK* | - | - | + | - |
|  | Dm | *csk* | - | - | - | FBbt:00004508 (eye), FBbt:00004510 (ommatidium) |
| ABL | Hs | *ABL1* | - | - | - | + |
|  | Hs | *ABL2* | - | - | - | + |
|  | Dm | *Abl* | - | - | - | FBbt:00005103 (commissure), FBbt:00001103 (longitudinal connective), FBbt:00001056 (embryonic central nervous system), FBbt:00001997 (anterior fascicle), FBbt:00001104 (ventral nerve cord commissure), FBbt:00001446 (embryonic neuron), FBbt:00005094 (central nervous system), FBbt:00004510 (ommatidium), FBbt:00004133 (interommatidial bristle), FBbt:00006009 (eye photoreceptor cell), FBbt:00003925 (antennal glomerulus), FBbt:00003979 (antennal commissure), FBbt:00004508 (eye) |
| TEC | Hs | *BMX* | - | - | - | + |
|  | Hs | *BTK* | - | - | + | - |
|  | Hs | *ITK* | - | - | - | - |
|  | Hs | *TEC* | - | - | - | - |
|  | Hs | *TXK* | - | - | + | - |
|  | Dm | *Btk29A* | FBbt:00003524 (basal apodeme of penis) | - | - | - |
| GRB2 | Hs | *GRAP* | + | - | - | - |
|  | Hs | *GRB2* | - | - | - | - |
|  | Dm | *drk* | - | - | - | FBbt:00001997 (anterior fascicle), FBbt:00005179 (macrochaeta), FBbt:00004225 (photoreceptor cell R7), FBbt:00002905 (abdominal 1 ventral monoscolopidial chordotonal organ vch1), FBbt:00002906 (abdominal 2 ventral monoscolopidial chordotonal organ vch1), FBbt:00002907 (abdominal 3 ventral monoscolopidial chordotonal organ vch1), FBbt:00002908 (abdominal 4 ventral monoscolopidial chordotonal organ vch1), FBbt:00002909 (abdominal 5 ventral monoscolopidial chordotonal organ vch1), FBbt:00002910 (abdominal 6 ventral monoscolopidial chordotonal organ vch1), FBbt:00002911 (abdominal 7 ventral monoscolopidial chordotonal organ vch1), FBbt:00002832 (abdominal 1 lateral pentascolopidial chordotonal organ lch5), FBbt:00002833 (abdominal 2 lateral pentascolopidial chordotonal organ lch5), FBbt:00002834 (abdominal 3 lateral pentascolopidial chordotonal organ lch5), FBbt:00002835 (abdominal 4 lateral pentascolopidial chordotonal organ lch5), FBbt:00002836 (abdominal 5 lateral pentascolopidial chordotonal organ lch5), FBbt:00002837 (abdominal 6 lateral pentascolopidial chordotonal organ lch5), etc |
| BLNK | Hs | *BLNK* | - | - | + | - |
|  | Dm | CG15529 | - | - | - | - |
| SOS | Hs | *SOS1* | - | - | - | + |
|  | Hs | *SOS2* | - | + | - | + |
|  | Dm | *Sos* | - | - | - | FBbt:00004510 (ommatidium), FBbt:00005179 (macrochaeta), FBbt:00004213 (photoreceptor cell R1), FBbt:00004215 (photoreceptor cell R2), FBbt:00004217 (photoreceptor cell R3), FBbt:00004219 (photoreceptor cell R4), FBbt:00004221 (photoreceptor cell R5), FBbt:00004223 (photoreceptor cell R6), FBbt:00004225 (photoreceptor cell R7), FBbt:00004227 (photoreceptor cell R8), FBbt:00004508 (eye), FBbt:00001448 (pCC neuron), FBbt:00001602 (dMP2 neuron), FBbt:00001603 (vMP2 neuron), FBbt:00001103 (longitudinal connective), FBbt:00002905 (abdominal 1 ventral monoscolopidial chordotonal organ vch1), FBbt:00002906 (abdominal 2 ventral monoscolopidial chordotonal organ vch1), FBbt:00002907 (abdominal 3 ventral monoscolopidial chordotonal organ vch1), FBbt:00002908 (abdominal 4 ventral monoscolopidial chordotonal organ vch1), FBbt:00002909 (abdominal 5 ventral monoscolopidial chordotonal organ vch1), etc. |
| RAS | Hs | *HRAS* | + | - | - | + |
|  | Hs | *KRAS* | - | - | - | + |
|  | Dm | *Ras85D* | FBbt:00000596 (abdominal ventral acute muscle 2), FBbt:00000464 (embryonic/larval somatic muscle), FBbt:00000467 (dorsal oblique muscle), FBbt:00000475 (dorsal acute muscle), FBbt:00005084 (muscle founder cell) | FBbt:00005058 (pericardial cell), FBbt:00005701 (larval hemocyte), FBbt:00005064 (melanotic mass), FBbt:00005063 (hemocyte) | FBbt:00005701 (larval hemocyte), FBbt:00005064 (melanotic mass), FBbt:00005063 (hemocyte) | FBbt:00004215 (photoreceptor cell R2), FBbt:00004217 (photoreceptor cell R3), FBbt:00004219 (photoreceptor cell R4), FBbt:00004221 (photoreceptor cell R5), FBbt:00004227 (photoreceptor cell R8), FBbt:00004510 (ommatidium), FBbt:00005839 (type I bouton), FBbt:00004225 (photoreceptor cell R7), FBbt:00006009 (eye photoreceptor cell), FBbt:00004211 (photoreceptor cell), FBbt:00004508 (eye), FBbt:00001311 (interface glial cell), FBbt:00001448 (pCC neuron), FBbt:00001602 (dMP2 neuron), FBbt:00001603 (vMP2 neuron), FBbt:00001134 (embryonic peripheral nervous system), FBbt:00005116 (bouton), FBbt:00004193 (cone cell), FBbt:00004133 (interommatidial bristle), FBbt:00001315 (midline glial cell), FBbt:00001137 (sensory mother cell), FBbt:00005177 (chaeta), FBbt:00005184 (sensillum trichodeum), FBbt:00004466 (gonopod thorn bristle), FBbt:00004232 (secondary pigment cell), FBbt:00004233 (tertiary pigment cell), FBbt:00004213 (photoreceptor cell R1), FBbt:00004223 (photoreceptor cell R6), FBbt:00004230 (pigment cell), FBbt:00005179 (macrochaeta), etc. |
| RAF | Hs | *ARAF* | - | - | - | - |
|  | Hs | *BRAF* | - | - | - | + |
|  | Hs | *RAF1* | - | + | - | + |
|  | Dm | *phl* | FBbt:00000596 (abdominal ventral acute muscle 2) | FBbt:00001687 (lamellocyte), FBbt:00005063 (hemocyte), FBbt:00005701 (larval hemocyte) | FBbt:00001687 (lamellocyte), FBbt:00005063 (hemocyte), FBbt:00005701 (larval hemocyte) | FBbt:00004508 (eye), FBbt:00004225 (photoreceptor cell R7), FBbt:00004510 (ommatidium), FBbt:00004213 (photoreceptor cell R1), FBbt:00004215 (photoreceptor cell R2), FBbt:00004217 (photoreceptor cell R3), FBbt:00004219 (photoreceptor cell R4), FBbt:00004221 (photoreceptor cell R5), FBbt:00004223 (photoreceptor cell R6), FBbt:00006009 (eye photoreceptor cell), FBbt:00004193 (cone cell), FBbt:00004227 (photoreceptor cell R8), FBbt:00005094 (central nervous system), FBbt:00005098 (peripheral nervous system), FBbt:00005215 (chordotonal organ), FBbt:00000093 (ventral midline), FBbt:00001920 (larval brain), FBbt:00001930 (larval optic lobe), FBbt:00002650 (larval labral sense organ), FBbt:00002951 (abdominal 11 anal tuft), FBbt:00004211 (photoreceptor cell), FBbt:00004133 (interommatidial bristle), FBbt:00005179 (macrochaeta), FBbt:00004323 (wing sensillum), FBbt:00004304 (anterior supraalar bristle), etc. |
| FOS | Hs | *FOS* | - | - | + | - |
|  | Hs | *FOSB* | - | - | - | - |
|  | Hs | *FOSL2* | + | - | - | + |
|  | Dm | *kay* | - | FBbt:00005684 (embryonic heart) | - | FBbt:00004508 (eye), FBbt:00004230 (pigment cell), FBbt:00004510 (ommatidium), FBbt:00005116 (bouton), FBbt:00006009 (eye photoreceptor cell), FBbt:00001056 (embryonic central nervous system), FBbt:00005103 (commissure), FBbt:00001134 (embryonic peripheral nervous system), FBbt:00001102 (ventral nerve cord), FBbt:00001103 (longitudinal connective), FBbt:00005144 (glial cell), FBbt:00001315 (midline glial cell) |
| JUN | Hs | *JUN* | - | - | - | - |
|  | Hs | *JUNB* | - | - | + | - |
|  | Hs | *JUND* | - | - | - | - |
|  | Dm | *Jra* | - | - | - | FBbt:00004510 (ommatidium), FBbt:00004217 (photoreceptor cell R3), FBbt:00004219 (photoreceptor cell R4), FBbt:00004211 (photoreceptor cell), FBbt:00004508 (eye), FBbt:00006009 (eye photoreceptor cell), FBbt:00004225 (photoreceptor cell R7), FBbt:00005103 (commissure), FBbt:00004193 (cone cell), FBbt:00005116 (bouton), FBbt:00004213 (photoreceptor cell R1), FBbt:00004215 (photoreceptor cell R2), FBbt:00004221 (photoreceptor cell R5), FBbt:00004223 (photoreceptor cell R6) |
| MAP3K-1 | Hs | *MAP3K7* | - | - | - | + |
|  | Dm | *Tak1* | - | - | - | FBbt:00005182 (microchaeta), FBbt:00004508 (eye), FBbt:00004133 (interommatidial bristle), FBbt:00004232 (secondary pigment cell), FBbt:00004233 (tertiary pigment cell), FBbt:00006009 (eye photoreceptor cell), FBbt:00004510 (ommatidium), FBbt:00004211 (photoreceptor cell) |
| MAP3K-2 | Hs | *MAP3K4* | - | - | - | - |
|  | Dm | *Mekk1* | - | - | - | - |
| JNK | Hs | *MAPK10* | - | - | - | + |
|  | Hs | *MAPK8* | no probes |  |  |  |
|  | Hs | *MAPK9* | - | - | - | + |
|  | Dm | *bsk* | - | - | - | FBbt:00004508 (eye), FBbt:00005116 (bouton), FBbt:00004510 (ommatidium), FBbt:00004211 (photoreceptor cell) |
| cMAPK | Hs | *MAPK1* | - | - | - | + |
|  | Hs | *MAPK3* | no probes |  |  |  |
|  | Dm | *rl* | - | - | - | FBbt:00004508 (eye), FBbt:00004213 (photoreceptor cell R1), FBbt:00004215 (photoreceptor cell R2), FBbt:00004217 (photoreceptor cell R3), FBbt:00004219 (photoreceptor cell R4), FBbt:00004221 (photoreceptor cell R5), FBbt:00004223 (photoreceptor cell R6), FBbt:00004225 (photoreceptor cell R7), FBbt:00004510 (ommatidium), FBbt:00004211 (photoreceptor cell), FBbt:00004193 (cone cell), FBbt:00005179 (macrochaeta), FBbt:00001315 (midline glial cell), FBbt:00004230 (pigment cell), FBbt:00006009 (eye photoreceptor cell), FBbt:00005116 (bouton) |
| MAP2K-1 | Hs | *MAP2K3* | - | - | - | - |
|  | Hs | *MAP2K6* | + | - | - | + |
|  | Dm | *lic* | - | - | - | - |
| MAP2K-2 | Hs | *MAP2K1* | - | - | - | + |
|  | Hs | *MAP2K2* | - | - | - | - |
|  | Dm | *Dsor1* | - | - | - | FBbt:00004508 (eye), FBbt:00004230 (pigment cell), FBbt:00004225 (photoreceptor cell R7), FBbt:00004510 (ommatidium), FBbt:00006009 (eye photoreceptor cell), FBbt:00004213 (photoreceptor cell R1), FBbt:00004215 (photoreceptor cell R2), FBbt:00004217 (photoreceptor cell R3), FBbt:00004219 (photoreceptor cell R4), FBbt:00004221 (photoreceptor cell R5), FBbt:00004223 (photoreceptor cell R6) |
| MAP2K-3 | Hs | *MAP2K7* | + | - | - | + |
|  | Dm | *hep* | - | FBbt:00001687 (lamellocyte), FBbt:00005064 (melanotic mass) | FBbt:00001687 (lamellocyte), FBbt:00005064 (melanotic mass) | FBbt:00005177 (chaeta), FBbt:00004508 (eye), FBbt:00005179 (macrochaeta), FBbt:00004510 (ommatidium), FBbt:00004133 (interommatidial bristle), FBbt:00004230 (pigment cell) |
| MAP2K-4 | Hs | *MAP2K4* | no probes |  |  |  |
|  | Dm | *Mkk4* | - | - | - | - |
| RAC | Hs | ENSG00000172895 | - | - | + | - |
|  | Hs | *RAC1* | - | - | + | + |
|  | Hs | *RAC2* | - | + | + | - |
|  | Hs | *RAC3* | - | - | - | - |
|  | Dm | *Rac1* | FBbt:00005083 (myoblast), FBbt:00003362 (dorsal medial muscle), FBbt:00003369 (lateral oblique dorsal muscle), FBbt:00003372 (tergosternal muscle), FBbt:00003376 (coxal tergal remotor muscle), FBbt:00003232 (adult myoblast), FBbt:00000599 (abdominal lateral longitudinal muscle), FBbt:00000464 (embryonic/larval somatic muscle) | FBbt:00005700 (embryonic hemocyte), FBbt:00005063 (hemocyte), FBbt:00001687 (lamellocyte), FBbt:00001690 (crystal cell), FBbt:00005064 (melanotic mass) | FBbt:00005700 (embryonic hemocyte), FBbt:00005063 (hemocyte), FBbt:00001687 (lamellocyte), FBbt:00001690 (crystal cell), FBbt:00005064 (melanotic mass) | FBbt:00004510 (ommatidium), FBbt:00004508 (eye), FBbt:00004133 (interommatidial bristle), FBbt:00005162 (photoreceptor), FBbt:00004230 (pigment cell), FBbt:00003684 (adult mushroom body), FBbt:00005213 (dendritic arborising neuron), FBbt:00002027 (dorsal multidendritic neuron ddaC), FBbt:00001327 (peripheral glial cell), FBbt:00004046 (ventral adult lateral neuron), FBbt:00002319 (abdominal anterior fascicle), FBbt:00003708 (lamina), FBbt:00003748 (medulla), FBbt:00002450 (abdominal posterior fascicle), FBbt:00005123 (motor neuron), FBbt:00001997 (anterior fascicle), FBbt:00000093 (ventral midline), FBbt:00005908 (medial longitudinal fascicle), FBbt:00001103 (longitudinal connective), FBbt:00006009 (eye photoreceptor cell), FBbt:00004211 (photoreceptor cell), FBbt:00005179 (macrochaeta), FBbt:00005183 (sensillum campaniformium), FBbt:00001448 (pCC neuron), FBbt:00001602 (dMP2 neuron), FBbt:00001603 (vMP2 neuron), FBbt:00001592 (VUM neuron), FBbt:00001104 (ventral nerve cord commissure), FBbt:00001102 (ventral nerve cord), etc. |
|  | Dm | *Rac2* | FBbt:00000463 (embryonic myoblast), FBbt:00005084 (muscle founder cell), FBbt:00005649 (embryonic somatic muscle) | FBbt:00005058 (pericardial cell), FBbt:00005678 (embryonic pericardial cell) | - | FBbt:00001327 (peripheral glial cell), FBbt:00005133 (serotonin neuron), FBbt:00004508 (eye), FBbt:00004510 (ommatidium), FBbt:00005162 (photoreceptor), FBbt:00004230 (pigment cell) |
| CDC42 | Hs | *CDC42* | - | + | + | - |
|  | Hs | ENSG00000152994 | - | + | + | - |
|  | Dm | *Cdc42* | FBbt:00005073 (somatic muscle) | FBbt:00005700 (embryonic hemocyte) | FBbt:00005700 (embryonic hemocyte) | FBbt:00004508 (eye), FBbt:00004510 (ommatidium), FBbt:00004133 (interommatidial bristle), FBbt:00001056 (embryonic central nervous system), FBbt:00001103 (longitudinal connective), FBbt:00003634 (vertical fiber system), FBbt:00005162 (photoreceptor), FBbt:00004230 (pigment cell), FBbt:00005182 (microchaeta), FBbt:00004326 (wing margin bristle), FBbt:00004331 (medial triple row), FBbt:00001134 (embryonic peripheral nervous system), FBbt:00002319 (abdominal anterior fascicle), FBbt:00001190 (dorsal abdominal cluster), FBbt:00003979 (antennal commissure), FBbt:00003925 (antennal glomerulus), FBbt:00005386 (glomerulus), FBbt:00001453 (RP2 neuron), FBbt:00001448 (pCC neuron), FBbt:00001602 (dMP2 neuron), FBbt:00001603 (vMP2 neuron), FBbt:00002450 (abdominal posterior fascicle), FBbt:00004020 (giant fibers) |
| RAP1 | Hs | ENSG00000176276 | - | + | + | - |
|  | Hs | *RAP1A* | no probes |  |  |  |
|  | Hs | *RAP1B* | - | + | + | - |
|  | Dm | *R* | - | FBbt:00001673 (embryonic/larval dorsal vessel), FBbt:00001686 (macrophage) | FBbt:00001686 (macrophage) | FBbt:00004232 (secondary pigment cell), FBbt:00004213 (photoreceptor cell R1), FBbt:00004215 (photoreceptor cell R2), FBbt:00004217 (photoreceptor cell R3), FBbt:00004219 (photoreceptor cell R4), FBbt:00004221 (photoreceptor cell R5), FBbt:00004223 (photoreceptor cell R6), FBbt:00004225 (photoreceptor cell R7), FBbt:00004227 (photoreceptor cell R8), FBbt:00004508 (eye), FBbt:00006009 (eye photoreceptor cell), FBbt:00004510 (ommatidium), FBbt:00001102 (ventral nerve cord) |
| VAV | Hs | *VAV1* | no probes |  |  |  |
|  | Hs | *VAV2* | - | - | - | - |
|  | Hs | *VAV3* | - | - | - | + |
|  | Dm | *vav* | - | - | - | - |
| SHC | Hs | *SHC1* | + | - | - | - |
|  | Hs | *SHC2* | - | - | - | + |
|  | Hs | *SHC3* | - | - | - | + |
|  | Hs | *SHC4* | no probes |  |  |  |
|  | Dm | *Shc* | - | - | - | FBbt:00004508 (eye), FBbt:00004510 (ommatidium), FBbt:00006009 (eye photoreceptor cell) |
| GAB | Hs | *GAB1* | - | - | - | + |
|  | Hs | *GAB2* | - | + | - | + |
|  | Hs | *GAB3* | no probes |  |  |  |
|  | Dm | *dos* | FBbt:00000596 (abdominal ventral acute muscle 2) | - | - | FBbt:00004508 (eye), FBbt:00005162 (photoreceptor), FBbt:00004225 (photoreceptor cell R7), FBbt:00006009 (eye photoreceptor cell), FBbt:00004510 (ommatidium), FBbt:00004193 (cone cell) |
| “+” indicates that a human gene is specifically expressed (Z-score ≧ 2) in at least one tissue or organ that is classified into the listed system-level function category; otherwise, “–” is indicated. For Drosophila genes, anatomic ontology terms are listed according to FlyBase annotation; descriptions of terms are given in parentheses. | | | | | | |
| Dm, *Drosophila melanogaster*; Hs, *Homo sapiens*; AIS, adaptive immune system. | | | | | | |
