# Supplementary material for: Expansion of signaling genes for adaptive immune system evolution in early vertebrates
Source: BMC Genomics. 2008 May 14;9:218. doi: 10.1186/1471-2164-9-218 (PMC2391169; doi:10.1186/1471-2164-9-218)
Supplement: Additional file 11 — Assignment of system-level function categories to human and Drosophila genes in AIS subfamilies according to published data and FlyBase annotations. For Drosophila genes, anatomic ontology terms are listed according to FlyBase annotation; descriptions of terms are given in parentheses. Dm, Drosophila melanogaster; Hs, Homo sapiens; AIS, adaptive immune system. [file 1471-2164-9-218-S11.doc]

| **Additional file 11. Assignment of system-level function categories to human and *Drosophila* genes in AIS subfamilies according to published data and FlyBase annotations** | | | | |
| --- | --- | --- | --- | --- |
| AIS subfamily | Species | Member | Nervous system | Adaptive immune system |
| JAK | Hs | *JAK1* | neuron survival [1] | T and B cell development [1] |
|  | Hs | *JAK2* | neuron survival [2] |  |
|  | Hs | *JAK3* | seizure activities and neuron survival [3] | T and B cell development [4] |
|  | Dm | *hop* | FBbt:00004510 (ommatidium), FBbt:00004508 (eye), FBbt:00004509 (eye equator), FBbt:00006009 (eye photoreceptor cell), FBbt:00004133 (interommatidial bristle), FBbt:00005098 (peripheral nervous system), FBbt:00001920 (larval brain) |  |
| PIAS | Hs | *PIAS1* |  |  |
|  | Hs | *PIAS2* | hindbrain development [5] | interleukin-12 signal transduction in T cells [6] |
|  | Hs | *PIAS3* |  |  |
|  | Hs | *PIAS4* |  |  |
|  | Dm | *Su(var)2-10* | FBbt:00004199 (lens), FBbt:00004508 (eye), FBbt:00004510 (ommatidium), FBbt:00004211 (photoreceptor cell) |  |
| STAT | Hs | *STAT5A* |  | T cell differentiation [7] |
|  | Hs | *STAT5B* | prolactin signal transduction in neurons [8] | T cell proliferation and B cell development [9] |
|  | Hs | *STAT6* | behavioral responses [10] | T cell differentiation and immunoglobulin class switch in B cells [11]; B cell migration [12] |
|  | Dm | *Stat92E* | FBbt:00005094 (central nervous system), FBbt:00005103 (commissure), FBbt:00001103 (longitudinal connective), FBbt:00005098 (peripheral nervous system), FBbt:00004508 (eye), FBbt:00004510 (ommatidium) |  |
| SOCS | Hs | *SOCS4* |  |  |
|  | Hs | *SOCS5* |  | T cell differentiation [13] |
|  | Dm | *Socs36E* | FBbt:00004230 (pigment cell) |  |
| SHP | Hs | *PTPN11* | neuron survival [14] | T cell development and proliferation [15] |
|  | Hs | *PTPN6* | microglia cell proliferation [16] | T cell differentiation [17] |
|  | Dm | *csw* | FBbt:00000113 (neurectoderm), FBbt:00005103 (commissure), FBbt:00001103 (longitudinal connective), FBbt:00004510 (ommatidium), FBbt:00004133 (interommatidial bristle), FBbt:00004217 (photoreceptor cell R3), FBbt:00004219 (photoreceptor cell R4), FBbt:00004225 (photoreceptor cell R7), FBbt:00006009 (eye photoreceptor cell), FBbt:00006007 (outer photoreceptor cell), FBbt:00004508 (eye), FBbt:00004211 (photoreceptor cell), FBbt:00005162 (photoreceptor) |  |
| PRKAR | Hs | *PRKAR1A* |  | responsibility for systemic lupus erythematosus [18] |
|  | Hs | *PRKAR1B* | long-term depression and depotentiation at synapse [19] |  |
|  | Dm | *Pka-R1* | FBbt:00004312 (scutellar bristle), FBbt:00004246 (leg sensillum) |  |
| GNG | Hs | *GNG12* |  |  |
|  | Hs | *GNG2* |  |  |
|  | Hs | *GNG3* | seizure activities and leptin signal transduction [20] | antibody responses [21] |
|  | Hs | *GNG4* |  |  |
|  | Hs | *GNG5* |  |  |
|  | Hs | *GNG8* |  |  |
|  | Dm | *Ggamma1* | FBbt:00005146 (neuroblast), FBbt:00005149 (ganglion mother cell), FBbt:00001332 (embryonic neuroblast) |  |
| GNB | Hs | *GNB1* | responsibility for Rd4 retinal disease [22] |  |
|  | Hs | *GNB2* |  |  |
|  | Hs | *GNB3* |  | T cell proliferation and migration [23] |
|  | Hs | *GNB4* |  |  |
|  | Dm | *Gbeta13F* | FBbt:00001332 (embryonic neuroblast), FBbt:00005146 (neuroblast), FBbt:00001572 (EL neuron) |  |
| GNA | Hs | *GNAI1* | memory formation and long-term potentiation at synapse [24] |  |
|  | Hs | *GNAI2* | vomeronasal neuron development [25] | B cell development [26]; proliferation and cytokine production in T cells [27] |
|  | Hs | *GNAI3* |  |  |
|  | Dm | *G-ialpha65A* | FBbt:00005146 (neuroblast), FBbt:00001454 (RP2sib neuron), FBbt:00001453 (RP2 neuron), FBbt:00005177 (chaeta), FBbt:00001332 (embryonic neuroblast), FBbt:00004331 (medial triple row) |  |
| RHO | Hs | *RHOA* | long-lasting potentiation [28] | T cell adhesion [29] |
|  | Hs | *RHOC* |  |  |
|  | Dm | *Rho1* | FBbt:00004510 (ommatidium), FBbt:00006009 (eye photoreceptor cell), FBbt:00005801 (mushroom body), FBbt:00001327 (peripheral glial cell), FBbt:00004232 (secondary pigment cell), FBbt:00004233 (tertiary pigment cell), FBbt:00004211 (photoreceptor cell), FBbt:00004508 (eye), FBbt:00004200 (retina), FBbt:00004230 (pigment cell), FBbt:00005164 (external sensory organ precursor cell), FBbt:00001967 (Bolwig's nerve), FBbt:00005407 (dorsal lobe), FBbt:00001455 (RP3 neuron) |  |
| DGK | Hs | *DGKA* |  | T cell anergy [30] |
|  | Hs | *DGKB* |  |  |
|  | Hs | *DGKG* |  |  |
|  | Dm | *Dgk* |  |  |
| PLCG | Hs | *PLCG1* |  | development and proliferation in B cells [31] |
|  | Hs | *PLCG2* |  | B cell development [31] |
|  | Dm | *sl* | FBbt:00004510 (ommatidium), FBbt:00004225 (photoreceptor cell R7), FBbt:00004508 (eye), FBbt:00004133 (interommatidial bristle), FBbt:00006009 (eye photoreceptor cell), FBbt:00004193 (cone cell), FBbt:00004231 (primary pigment cell) |  |
| aPKC | Hs | *PRKCI* |  |  |
|  | Hs | *PRKCZ* | neuron survival [32] | differentiation and cytokine production in T cells [33]; proliferation and survival in B cells [34] |
|  | Dm | *aPKC* | FBbt:00004510 (ommatidium), FBbt:00006009 (eye photoreceptor cell), FBbt:00005116 (bouton) |  |
| nPKC | Hs | *PRKCD* |  | interleukin-6 production and proliferation in B cells [35] |
|  | Hs | *PRKCQ* | synapse development [36] | interleukin-2 production and proliferation in T cells [37] |
|  | Dm | *Pkcdelta* |  |  |
| cPKC | Hs | *PRKCA* |  | degranulation in cytotoxic T lymphocytes [38] |
|  | Hs | *PRKCB1* | neural tube development; hindgut proliferation [39] | B cell survival [40] |
|  | Hs | *PRKCG* | neural tube development; hindgut proliferation [39] |  |
|  | Dm | *Pkc53E* |  |  |
| CAMK2 | Hs | *CAMK2A* | learning [41] |  |
|  | Hs | *CAMK2B* |  |  |
|  | Hs | *CAMK2D* |  |  |
|  | Hs | *CAMK2G* |  | differentiation and survival in T cells [42] |
|  | Dm | *CaMKII* |  |  |
| CALNA | Hs | *PPP3CA* | synaptic depontentiation [43] | T cell proliferation [44] |
|  | Hs | *PPP3CB* |  | development and proliferation in T cells [45] |
|  | Dm | *CanA-14F* |  |  |
|  | Dm | *Pp2B-14D* | FBbt:00004508 (eye), FBbt:00004510 (ommatidium), FBbt:00004133 (interommatidial bristle), FBbt:00006009 (eye photoreceptor cell), FBbt:00004225 (photoreceptor cell R7), FBbt:00004217 (photoreceptor cell R3), FBbt:00004219 (photoreceptor cell R4) |  |
| CALNB | Hs | *PPP3R1* | long-term depontentiation and learning [46] | T cell development [47] |
|  | Dm | CG14353 |  |  |
| NFAT | Hs | *NFAT5* |  | T cell development [48] |
|  | Dm | *NFAT* | FBbt:00001997 (anterior fascicle), FBbt:00004508 (eye), FBbt:00004306 (dorsocentral bristle), FBbt:00004312 (scutellar bristle), FBbt:00005169 (trichogen cell) |  |
| IKBK | Hs | *CHUK* |  | development and immunoglobulin production in B cells[49]; T cell survival [50] |
|  | Hs | *IKBKB* | neuron outgrowth [51] | T cell proliferation and survival [50]; survival and immunoglobulin production in B cells [52] |
|  | Dm | *ird5* |  |  |
| NFKB | Hs | *NFKB1* | learning [53] | T cell differentiation [54] |
|  | Hs | *NFKB2* |  | differentiation and cytokine production in T cells [55] |
|  | Hs | *REL* | long-term depression and memory formation [56] | T cell differentiation [54]; B cell proliferation and survival [57] |
|  | Hs | *RELA* | learning [58] | immunoglobulin production in B cells [59] |
|  | Hs | *RELB* |  | differentiation and cytokine production in T cells [55] |
|  | Dm | *dl* | FBbt:00005801 (mushroom body), FBbt:00003686 (Kenyon cell) |  |
|  | Dm | *Dif* |  |  |
| NFKBI | Hs | *BCL3* |  | differentiation and cytokine production in T cells [55] |
|  | Hs | *NFKBIA* | central nerve system protection [60]; neuron outgrowth [51] | T cell proliferation; proliferation and immunoglobulin production in B cells [61] |
|  | Hs | *NFKBIB* |  |  |
|  | Hs | *NFKBIE* |  | T cell development [62] |
|  | Dm | *cact* | FBbt:00001860 (dorsal fold) |  |
| PIK3C | Hs | *PIK3CA* |  |  |
|  | Hs | *PIK3CB* |  |  |
|  | Hs | *PIK3CD* |  | B cell migration [63] |
|  | Dm | *Pi3K92E* | FBbt:00004510 (ommatidium), FBbt:00004508 (eye), FBbt:00005177 (chaeta), FBbt:00004133 (interommatidial bristle) |  |
| PIK3R | Hs | *PIK3R1* | neuron survival [64] | B cell development [65] |
|  | Hs | *PIK3R2* |  | proliferation and survival in T cells [66] |
|  | Hs | *PIK3R3* |  |  |
|  | Dm | *Pi3K21B* |  |  |
| PTEN | Hs | *PTEN* | seizure activity and ataxia [67] | migration, proliferation and survival in B cells [68] |
|  | Dm | *Pten* | FBbt:00004508 (eye), FBbt:00004510 (ommatidium), FBbt:00004133 (interommatidial bristle), FBbt:00004211 (photoreceptor cell), FBbt:00001725 (larval corpus allatum), FBbt:00006009 (eye photoreceptor cell), FBbt:00004230 (pigment cell), FBbt:00004199 (lens) |  |
| AKT | Hs | *AKT1* | neuronal morphology and working memory formation [69] | T cell activation [70] |
|  | Hs | *AKT2* | photoreceptor cell survival [71] |  |
|  | Hs | *AKT3* | brain development [72] |  |
|  | Dm | *Akt1* | FBbt:00004510 (ommatidium), FBbt:00004508 (eye), FBbt:00004133 (interommatidial bristle), FBbt:00004326 (wing margin bristle) |  |
| SRC | Hs | *CSK* | neurite outgrowth [73] | T cell development [74] |
|  | Hs | *MATK* |  | development and cytokine production in B cells [75] |
|  | Dm | *csk* | FBbt:00004508 (eye), FBbt:00004510 (ommatidium) |  |
| ABL | Hs | *ABL1* | dendrite outgrowth [76] | B cell proliferation [77] |
|  | Hs | *ABL2* | axon and dendrite branching [78] |  |
|  | Dm | *Abl* | FBbt:00005103 (commissure), FBbt:00001103 (longitudinal connective), FBbt:00001056 (embryonic central nervous system), FBbt:00001997 (anterior fascicle), FBbt:00001104 (ventral nerve cord commissure), FBbt:00001446 (embryonic neuron), FBbt:00005094 (central nervous system), FBbt:00004510 (ommatidium), FBbt:00004133 (interommatidial bristle), FBbt:00006009 (eye photoreceptor cell), FBbt:00003925 (antennal glomerulus), FBbt:00003979 (antennal commissure), FBbt:00004508 (eye) |  |
| TEC | Hs | *BMX* | neurite outgrowth [79] |  |
|  | Hs | *BTK* |  | B cell development [80] |
|  | Hs | *ITK* |  | cytokine production and differentiation in T cells [81] |
|  | Hs | *TEC* |  | T cell activation [82] |
|  | Hs | *TXK* |  | development and cytokine production in T cells [83] |
|  | Dm | *Btk29A* |  |  |
| GRB2 | Hs | *GRAP* |  | proliferation and cytokine production in T cells [84] |
|  | Hs | *GRB2* | cranial nerve organization [85] | T cell development [86] |
|  | Dm | *drk* | FBbt:00001997 (anterior fascicle), FBbt:00005179 (macrochaeta), FBbt:00004225 (photoreceptor cell R7), FBbt:00002905 (abdominal 1 ventral monoscolopidial chordotonal organ vch1), FBbt:00002906 (abdominal 2 ventral monoscolopidial chordotonal organ vch1), FBbt:00002907 (abdominal 3 ventral monoscolopidial chordotonal organ vch1), FBbt:00002908 (abdominal 4 ventral monoscolopidial chordotonal organ vch1), FBbt:00002909 (abdominal 5 ventral monoscolopidial chordotonal organ vch1), FBbt:00002910 (abdominal 6 ventral monoscolopidial chordotonal organ vch1), FBbt:00002911 (abdominal 7 ventral monoscolopidial chordotonal organ vch1), FBbt:00002832 (abdominal 1 lateral pentascolopidial chordotonal organ lch5), FBbt:00002833 (abdominal 2 lateral pentascolopidial chordotonal organ lch5), FBbt:00002834 (abdominal 3 lateral pentascolopidial chordotonal organ lch5), FBbt:00002835 (abdominal 4 lateral pentascolopidial chordotonal organ lch5), FBbt:00002836 (abdominal 5 lateral pentascolopidial chordotonal organ lch5), FBbt:00002837 (abdominal 6 lateral pentascolopidial chordotonal organ lch5), etc. |  |
| BLNK | Hs | *BLNK* |  | B cell development [87] |
|  | Dm | CG15529 |  |  |
| SOS | Hs | *SOS1* | neurite outgrowth [88] | T cell proliferation [89] |
|  | Hs | *SOS2* | neurite outgrowth [88] |  |
|  | Dm | *Sos* | FBbt:00004510 (ommatidium), FBbt:00005179 (macrochaeta), FBbt:00004213 (photoreceptor cell R1), FBbt:00004215 (photoreceptor cell R2), FBbt:00004217 (photoreceptor cell R3), FBbt:00004219 (photoreceptor cell R4), FBbt:00004221 (photoreceptor cell R5), FBbt:00004223 (photoreceptor cell R6), FBbt:00004225 (photoreceptor cell R7), FBbt:00004227 (photoreceptor cell R8), FBbt:00004508 (eye), FBbt:00001448 (pCC neuron), FBbt:00001602 (dMP2 neuron), FBbt:00001603 (vMP2 neuron), FBbt:00001103 (longitudinal connective), FBbt:00002905 (abdominal 1 ventral monoscolopidial chordotonal organ vch1), FBbt:00002906 (abdominal 2 ventral monoscolopidial chordotonal organ vch1), FBbt:00002907 (abdominal 3 ventral monoscolopidial chordotonal organ vch1), FBbt:00002908 (abdominal 4 ventral monoscolopidial chordotonal organ vch1), FBbt:00002909 (abdominal 5 ventral monoscolopidial chordotonal organ vch1), etc. |  |
| RAS | Hs | *HRAS* | neuron survival [90] brain hypertrophy and neuron protection [91] | proliferation and development in T cells [92] |
|  | Hs | *KRAS* | long-term potentiation and behavioral responses [93] |  |
|  | Dm | *Ras85D* | FBbt:00004215 (photoreceptor cell R2), FBbt:00004217 (photoreceptor cell R3), FBbt:00004219 (photoreceptor cell R4), FBbt:00004221 (photoreceptor cell R5), FBbt:00004227 (photoreceptor cell R8), FBbt:00004510 (ommatidium), FBbt:00005839 (type I bouton), FBbt:00004225 (photoreceptor cell R7), FBbt:00006009 (eye photoreceptor cell), FBbt:00004211 (photoreceptor cell), FBbt:00004508 (eye), FBbt:00001311 (interface glial cell), FBbt:00001448 (pCC neuron), FBbt:00001602 (dMP2 neuron), FBbt:00001603 (vMP2 neuron), FBbt:00001134 (embryonic peripheral nervous system), FBbt:00005116 (bouton), FBbt:00004193 (cone cell), FBbt:00004133 (interommatidial bristle), FBbt:00001315 (midline glial cell), FBbt:00001137 (sensory mother cell), FBbt:00005177 (chaeta), FBbt:00005184 (sensillum trichodeum), FBbt:00004466 (gonopod thorn bristle), FBbt:00004232 (secondary pigment cell), FBbt:00004233 (tertiary pigment cell), FBbt:00004213 (photoreceptor cell R1), FBbt:00004223 (photoreceptor cell R6), FBbt:00004230 (pigment cell), FBbt:00005179 (macrochaeta), etc. |  |
| RAF | Hs | *ARAF* | behavioral responses and intestine development [94] |  |
|  | Hs | *BRAF* | long-term potentiation, lerning and memory [95] | cytokine production [96] |
|  | Hs | *RAF1* | ARIA signal transduction [97] | interleukin-2 production [98] |
|  | Dm | *phl* | FBbt:00004508 (eye), FBbt:00004225 (photoreceptor cell R7), FBbt:00004510 (ommatidium), FBbt:00004213 (photoreceptor cell R1), FBbt:00004215 (photoreceptor cell R2), FBbt:00004217 (photoreceptor cell R3), FBbt:00004219 (photoreceptor cell R4), FBbt:00004221 (photoreceptor cell R5), FBbt:00004223 (photoreceptor cell R6), FBbt:00006009 (eye photoreceptor cell), FBbt:00004193 (cone cell), FBbt:00004227 (photoreceptor cell R8), FBbt:00005094 (central nervous system), FBbt:00005098 (peripheral nervous system), FBbt:00005215 (chordotonal organ), FBbt:00000093 (ventral midline), FBbt:00001920 (larval brain), FBbt:00001930 (larval optic lobe), FBbt:00002650 (larval labral sense organ), FBbt:00002951 (abdominal 11 anal tuft), FBbt:00004211 (photoreceptor cell), FBbt:00004133 (interommatidial bristle), FBbt:00005179 (macrochaeta), FBbt:00004323 (wing sensillum), FBbt:00004304 (anterior supraalar bristle), etc. |  |
| FOS | Hs | *FOS* | long-term potentiation and learning [99] | B cell proliferation [100] |
|  | Hs | *FOSB* | electrically induced seizures [101] | T cell proliferation [102] |
|  | Hs | *FOSL2* |  |  |
|  | Dm | *kay* | FBbt:00004508 (eye), FBbt:00004230 (pigment cell), FBbt:00004510 (ommatidium), FBbt:00005116 (bouton), FBbt:00006009 (eye photoreceptor cell), FBbt:00001056 (embryonic central nervous system), FBbt:00005103 (commissure), FBbt:00001134 (embryonic peripheral nervous system), FBbt:00001102 (ventral nerve cord), FBbt:00001103 (longitudinal connective), FBbt:00005144 (glial cell), FBbt:00001315 (midline glial cell) |  |
| JUN | Hs | *JUN* | neuron survival [103] | interleukin-2 expression in T cells [104] |
|  | Hs | *JUNB* |  | differentiation and cytokine production in T cells [105] |
|  | Hs | *JUND* |  | proliferation, differentiation and cytokine production in T cells [106] |
|  | Dm | *Jra* | FBbt:00004510 (ommatidium), FBbt:00004217 (photoreceptor cell R3), FBbt:00004219 (photoreceptor cell R4), FBbt:00004211 (photoreceptor cell), FBbt:00004508 (eye), FBbt:00006009 (eye photoreceptor cell), FBbt:00004225 (photoreceptor cell R7), FBbt:00005103 (commissure), FBbt:00004193 (cone cell), FBbt:00005116 (bouton), FBbt:00004213 (photoreceptor cell R1), FBbt:00004215 (photoreceptor cell R2), FBbt:00004221 (photoreceptor cell R5), FBbt:00004223 (photoreceptor cell R6) |  |
| MAP3K-1 | Hs | *MAP3K7* |  | B cell proliferation [107] |
|  | Dm | *Tak1* | FBbt:00005182 (microchaeta), FBbt:00004508 (eye), FBbt:00004133 (interommatidial bristle), FBbt:00004232 (secondary pigment cell), FBbt:00004233 (tertiary pigment cell), FBbt:00006009 (eye photoreceptor cell), FBbt:00004510 (ommatidium), FBbt:00004211 (photoreceptor cell) |  |
| MAP3K-2 | Hs | *MAP3K4* | neural tube development [108] | cytokine production in T cells [109] |
|  | Dm | *Mekk1* |  |  |
| JNK | Hs | *MAPK10* | neuron outgrowth [110] |  |
|  | Hs | *MAPK8* | neurite outgrowth [111] | differentiation, cytokine production and proliferation in T cells [112] |
|  | Hs | *MAPK9* | long-term potentiation at synapse [113] | differentiation and cytokine production in T cells [114] |
|  | Dm | *bsk* | FBbt:00004508 (eye), FBbt:00005116 (bouton), FBbt:00004510 (ommatidium), FBbt:00004211 (photoreceptor cell) |  |
| cMAPK | Hs | *MAPK1* |  | T cell development [115] |
|  | Hs | *MAPK3* | locomotion activity, learning and long-term memory [116] | T cell development [117] |
|  | Dm | *rl* | FBbt:00004508 (eye), FBbt:00004213 (photoreceptor cell R1), FBbt:00004215 (photoreceptor cell R2), FBbt:00004217 (photoreceptor cell R3), FBbt:00004219 (photoreceptor cell R4), FBbt:00004221 (photoreceptor cell R5), FBbt:00004223 (photoreceptor cell R6), FBbt:00004225 (photoreceptor cell R7), FBbt:00004510 (ommatidium), FBbt:00004211 (photoreceptor cell), FBbt:00004193 (cone cell), FBbt:00005179 (macrochaeta), FBbt:00001315 (midline glial cell), FBbt:00004230 (pigment cell), FBbt:00006009 (eye photoreceptor cell), FBbt:00005116 (bouton) |  |
| MAP2K-1 | Hs | *MAP2K3* | neuron survival [118] | T cell survival [119] |
|  | Hs | *MAP2K6* |  | T cell survival [119] |
|  | Dm | *lic* |  |  |
| MAP2K-2 | Hs | *MAP2K1* |  | T cell development [120] |
|  | Hs | *MAP2K2* |  |  |
|  | Dm | *Dsor1* | FBbt:00004508 (eye), FBbt:00004230 (pigment cell), FBbt:00004225 (photoreceptor cell R7), FBbt:00004510 (ommatidium), FBbt:00006009 (eye photoreceptor cell), FBbt:00004213 (photoreceptor cell R1), FBbt:00004215 (photoreceptor cell R2), FBbt:00004217 (photoreceptor cell R3), FBbt:00004219 (photoreceptor cell R4), FBbt:00004221 (photoreceptor cell R5), FBbt:00004223 (photoreceptor cell R6) |  |
| MAP2K-3 | Hs | *MAP2K7* |  | cytokine production and proliferation in T cells [121] |
|  | Dm | *hep* | FBbt:00005177 (chaeta), FBbt:00004508 (eye), FBbt:00005179 (macrochaeta), FBbt:00004510 (ommatidium), FBbt:00004133 (interommatidial bristle), FBbt:00004230 (pigment cell) |  |
| MAP2K-4 | Hs | *MAP2K4* |  | T cell development [122]; cytokine production and proliferation in T cells [123] |
|  | Dm | *Mkk4* |  |  |
| RAC | Hs | ENSG00000172895 |  |  |
|  | Hs | *RAC1* | dendrite development [124] | T cell development [125] |
|  | Hs | *RAC2* |  | B cell development [126] |
|  | Hs | *RAC3* | neuritogenesis [127] |  |
|  | Dm | *Rac1* | FBbt:00004510 (ommatidium), FBbt:00004508 (eye), FBbt:00004133 (interommatidial bristle), FBbt:00005162 (photoreceptor), FBbt:00004230 (pigment cell), FBbt:00003684 (adult mushroom body), FBbt:00005213 (dendritic arborising neuron), FBbt:00002027 (dorsal multidendritic neuron ddaC), FBbt:00001327 (peripheral glial cell), FBbt:00004046 (ventral adult lateral neuron), FBbt:00002319 (abdominal anterior fascicle), FBbt:00003708 (lamina), FBbt:00003748 (medulla), FBbt:00002450 (abdominal posterior fascicle), FBbt:00005123 (motor neuron), FBbt:00001997 (anterior fascicle), FBbt:00000093 (ventral midline), FBbt:00005908 (medial longitudinal fascicle), FBbt:00001103 (longitudinal connective), FBbt:00006009 (eye photoreceptor cell), FBbt:00004211 (photoreceptor cell), FBbt:00005179 (macrochaeta), FBbt:00005183 (sensillum campaniformium), FBbt:00001448 (pCC neuron), FBbt:00001602 (dMP2 neuron), FBbt:00001603 (vMP2 neuron), FBbt:00001592 (VUM neuron), FBbt:00001104 (ventral nerve cord commissure), FBbt:00001102 (ventral nerve cord), etc. |  |
|  | Dm | *Rac2* | FBbt:00001327 (peripheral glial cell), FBbt:00005133 (serotonin neuron), FBbt:00004508 (eye), FBbt:00004510 (ommatidium), FBbt:00005162 (photoreceptor), FBbt:00004230 (pigment cell) |  |
| CDC42 | Hs | *CDC42* | dendrite initiation [124] | T cell activation [128] |
|  | Hs | ENSG00000152994 |  |  |
|  | Dm | *Cdc42* | FBbt:00004508 (eye), FBbt:00004510 (ommatidium), FBbt:00004133 (interommatidial bristle), FBbt:00001056 (embryonic central nervous system), FBbt:00001103 (longitudinal connective), FBbt:00003634 (vertical fiber system), FBbt:00005162 (photoreceptor), FBbt:00004230 (pigment cell), FBbt:00005182 (microchaeta), FBbt:00004326 (wing margin bristle), FBbt:00004331 (medial triple row), FBbt:00001134 (embryonic peripheral nervous system), FBbt:00002319 (abdominal anterior fascicle), FBbt:00001190 (dorsal abdominal cluster), FBbt:00003979 (antennal commissure), FBbt:00003925 (antennal glomerulus), FBbt:00005386 (glomerulus), FBbt:00001453 (RP2 neuron), FBbt:00001448 (pCC neuron), FBbt:00001602 (dMP2 neuron), FBbt:00001603 (vMP2 neuron), FBbt:00002450 (abdominal posterior fascicle), FBbt:00004020 (giant fibers) |  |
| RAP1 | Hs | ENSG00000176276 |  |  |
|  | Hs | *RAP1A* |  | cytokine production and proliferation in T cells [129] |
|  | Hs | *RAP1B* | neuron survival [130] |  |
|  | Dm | *R* | FBbt:00004232 (secondary pigment cell), FBbt:00004213 (photoreceptor cell R1), FBbt:00004215 (photoreceptor cell R2), FBbt:00004217 (photoreceptor cell R3), FBbt:00004219 (photoreceptor cell R4), FBbt:00004221 (photoreceptor cell R5), FBbt:00004223 (photoreceptor cell R6), FBbt:00004225 (photoreceptor cell R7), FBbt:00004227 (photoreceptor cell R8), FBbt:00004508 (eye), FBbt:00006009 (eye photoreceptor cell), FBbt:00004510 (ommatidium), FBbt:00001102 (ventral nerve cord) |  |
| VAV | Hs | *VAV1* |  | development and activation in T cells, B cell development [131] |
|  | Hs | *VAV2* | neurite outgrowth [88] | immunoglobulin production in B cells [132] |
|  | Hs | *VAV3* | neurite outgrowth [88] |  |
|  | Dm | *vav* |  |  |
| SHC | Hs | *SHC1* | responsibility for microencephaly [133] | T cell development [134] |
|  | Hs | *SHC2* | neural development [135] |  |
|  | Hs | *SHC3* | learning, memory and long-term potentiation [136] |  |
|  | Hs | *SHC4* |  |  |
|  | Dm | *Shc* | FBbt:00004508 (eye), FBbt:00004510 (ommatidium), FBbt:00006009 (eye photoreceptor cell) |  |
| GAB | Hs | *GAB1* | neurite outgrowth and survival [137] | development in T cells [138]; development and immunoglobulin production in B cells [138] |
|  | Hs | *GAB2* |  | activation and cytokine production in T cells [139] |
|  | Hs | *GAB3* |  |  |
|  | Dm | *dos* | FBbt:00004508 (eye), FBbt:00005162 (photoreceptor), FBbt:00004225 (photoreceptor cell R7), FBbt:00006009 (eye photoreceptor cell), FBbt:00004510 (ommatidium), FBbt:00004193 (cone cell) |  |
| For *Drosophila* genes, anatomic ontology terms are listed according to FlyBase annotations; descriptions of terms are given in parentheses. | | | | |
| Dm, *Drosophila melanogaster*; Hs, *Homo sapiens*; AIS, adaptive immune system. | | | | |

**Supplementary References**

1. Rodig SJ, Meraz MA, White JM, Lampe PA, Riley JK, Arthur CD, King KL, Sheehan KC, Yin L, Pennica D *et al*: **Disruption of the Jak1 gene demonstrates obligatory and nonredundant roles of the Jaks in cytokine-induced biologic responses**. *Cell* 1998, **93**(3):373-383.

2. Digicaylioglu M, Lipton SA: **Erythropoietin-mediated neuroprotection involves cross-talk between Jak2 and NF-kappaB signalling cascades**. *Nature* 2001, **412**(6847):641-647.

3. Yang DD, Kuan CY, Whitmarsh AJ, Rincon M, Zheng TS, Davis RJ, Rakic P, Flavell RA: **Absence of excitotoxicity-induced apoptosis in the hippocampus of mice lacking the Jnk3 gene**. *Nature* 1997, **389**(6653):865-870.

4. Park SY, Saijo K, Takahashi T, Osawa M, Arase H, Hirayama N, Miyake K, Nakauchi H, Shirasawa T, Saito T: **Developmental defects of lymphoid cells in Jak3 kinase-deficient mice**. *Immunity* 1995, **3**(6):771-782.

5. Garcia-Dominguez M, Gilardi-Hebenstreit P, Charnay P: **PIASxbeta acts as an activator of Hoxb1 and is antagonized by Krox20 during hindbrain segmentation**. *Embo J* 2006, **25**(11):2432-2442.

6. Arora T, Liu B, He H, Kim J, Murphy TL, Murphy KM, Modlin RL, Shuai K: **PIASx is a transcriptional co-repressor of signal transducer and activator of transcription 4**. *J Biol Chem* 2003, **278**(24):21327-21330.

7. Kagami S, Nakajima H, Suto A, Hirose K, Suzuki K, Morita S, Kato I, Saito Y, Kitamura T, Iwamoto I: **Stat5a regulates T helper cell differentiation by several distinct mechanisms**. *Blood* 2001, **97**(8):2358-2365.

8. Grattan DR, Xu J, McLachlan MJ, Kokay IC, Bunn SJ, Hovey RC, Davey HW: **Feedback regulation of PRL secretion is mediated by the transcription factor, signal transducer, and activator of transcription 5b**. *Endocrinology* 2001, **142**(9):3935-3940.

9. Burchill MA, Goetz CA, Prlic M, O'Neil JJ, Harmon IR, Bensinger SJ, Turka LA, Brennan P, Jameson SC, Farrar MA: **Distinct effects of STAT5 activation on CD4+ and CD8+ T cell homeostasis: development of CD4+CD25+ regulatory T cells versus CD8+ memory T cells**. *J Immunol* 2003, **171**(11):5853-5864.

10. Yukawa K, Iso H, Tanaka T, Tsubota Y, Owada-Makabe K, Bai T, Takeda K, Akira S, Maeda M: **Down-regulation of dopamine transporter and abnormal behavior in STAT6-deficient mice**. *Int J Mol Med* 2005, **15**(5):819-825.

11. Shimoda K, van Deursen J, Sangster MY, Sarawar SR, Carson RT, Tripp RA, Chu C, Quelle FW, Nosaka T, Vignali DA *et al*: **Lack of IL-4-induced Th2 response and IgE class switching in mice with disrupted Stat6 gene**. *Nature* 1996, **380**(6575):630-633.

12. Davey EJ, Greicius G, Thyberg J, Severinson E: **STAT6 is required for the regulation of IL-4-induced cytoskeletal events in B cells**. *Int Immunol* 2000, **12**(7):995-1003.

13. Seki Y, Hayashi K, Matsumoto A, Seki N, Tsukada J, Ransom J, Naka T, Kishimoto T, Yoshimura A, Kubo M: **Expression of the suppressor of cytokine signaling-5 (SOCS5) negatively regulates IL-4-dependent STAT6 activation and Th2 differentiation**. *Proc Natl Acad Sci U S A* 2002, **99**(20):13003-13008.

14. Aoki Y, Huang Z, Thomas SS, Bhide PG, Huang I, Moskowitz MA, Reeves SA: **Increased susceptibility to ischemia-induced brain damage in transgenic mice overexpressing a dominant negative form of SHP2**. *Faseb J* 2000, **14**(13):1965-1973.

15. Nguyen TV, Ke Y, Zhang EE, Feng GS: **Conditional deletion of Shp2 tyrosine phosphatase in thymocytes suppresses both pre-TCR and TCR signals**. *J Immunol* 2006, **177**(9):5990-5996.

16. Horvat A, Schwaiger F, Hager G, Brocker F, Streif R, Knyazev P, Ullrich A, Kreutzberg GW: **A novel role for protein tyrosine phosphatase shp1 in controlling glial activation in the normal and injured nervous system**. *J Neurosci* 2001, **21**(3):865-874.

17. Kamata T, Yamashita M, Kimura M, Murata K, Inami M, Shimizu C, Sugaya K, Wang CR, Taniguchi M, Nakayama T: **src homology 2 domain-containing tyrosine phosphatase SHP-1 controls the development of allergic airway inflammation**. *J Clin Invest* 2003, **111**(1):109-119.

18. Laxminarayana D, Khan IU, Kammer G: **Transcript mutations of the alpha regulatory subunit of protein kinase A and up-regulation of the RNA-editing gene transcript in lupus T lymphocytes**. *Lancet* 2002, **360**(9336):842-849.

19. Brandon EP, Zhuo M, Huang YY, Qi M, Gerhold KA, Burton KA, Kandel ER, McKnight GS, Idzerda RL: **Hippocampal long-term depression and depotentiation are defective in mice carrying a targeted disruption of the gene encoding the RI beta subunit of cAMP-dependent protein kinase**. *Proc Natl Acad Sci U S A* 1995, **92**(19):8851-8855.

20. Schwindinger WF, Giger KE, Betz KS, Stauffer AM, Sunderlin EM, Sim-Selley LJ, Selley DE, Bronson SK, Robishaw JD: **Mice with deficiency of G protein gamma3 are lean and have seizures**. *Mol Cell Biol* 2004, **24**(17):7758-7768.

21. Dubeykovskiy A, McWhinney C, Robishaw JD: **Runx-dependent regulation of G-protein gamma3 expression in T-cells**. *Cell Immunol* 2006, **240**(2):86-95.

22. Kitamura E, Danciger M, Yamashita C, Rao NP, Nusinowitz S, Chang B, Farber DB: **Disruption of the gene encoding the beta1-subunit of transducin in the Rd4/+ mouse**. *Invest Ophthalmol Vis Sci* 2006, **47**(4):1293-1301.

23. Lindemann M, Virchow S, Ramann F, Barsegian V, Kreuzfelder E, Siffert W, Muller N, Grosse-Wilde H: **The G protein beta3 subunit 825T allele is a genetic marker for enhanced T cell response**. *FEBS Lett* 2001, **495**(1-2):82-86.

24. Pineda VV, Athos JI, Wang H, Celver J, Ippolito D, Boulay G, Birnbaumer L, Storm DR: **Removal of G(ialpha1) constraints on adenylyl cyclase in the hippocampus enhances LTP and impairs memory formation**. *Neuron* 2004, **41**(1):153-163.

25. Norlin EM, Gussing F, Berghard A: **Vomeronasal phenotype and behavioral alterations in G alpha i2 mutant mice**. *Curr Biol* 2003, **13**(14):1214-1219.

26. Dalwadi H, Wei B, Schrage M, Spicher K, Su TT, Birnbaumer L, Rawlings DJ, Braun J: **B cell developmental requirement for the G alpha i2 gene**. *J Immunol* 2003, **170**(4):1707-1715.

27. Huang TT, Zong Y, Dalwadi H, Chung C, Miceli MC, Spicher K, Birnbaumer L, Braun J, Aranda R: **TCR-mediated hyper-responsiveness of autoimmune Galphai2(-/-) mice is an intrinsic naive CD4(+) T cell disorder selective for the Galphai2 subunit**. *Int Immunol* 2003, **15**(11):1359-1367.

28. Wang HG, Lu FM, Jin I, Udo H, Kandel ER, de Vente J, Walter U, Lohmann SM, Hawkins RD, Antonova I: **Presynaptic and postsynaptic roles of NO, cGK, and RhoA in long-lasting potentiation and aggregation of synaptic proteins**. *Neuron* 2005, **45**(3):389-403.

29. Vielkind S, Gallagher-Gambarelli M, Gomez M, Hinton HJ, Cantrell DA: **Integrin regulation by RhoA in thymocytes**. *J Immunol* 2005, **175**(1):350-357.

30. Zha Y, Marks R, Ho AW, Peterson AC, Janardhan S, Brown I, Praveen K, Stang S, Stone JC, Gajewski TF: **T cell anergy is reversed by active Ras and is regulated by diacylglycerol kinase-alpha**. *Nat Immunol* 2006, **7**(11):1166-1173.

31. Wen R, Chen Y, Schuman J, Fu G, Yang S, Zhang W, Newman DK, Wang D: **An important role of phospholipase Cgamma1 in pre-B-cell development and allelic exclusion**. *Embo J* 2004, **23**(20):4007-4017.

32. Koponen S, Kurkinen K, Akerman KE, Mochly-Rosen D, Chan PH, Koistinaho J: **Prevention of NMDA-induced death of cortical neurons by inhibition of protein kinase Czeta**. *J Neurochem* 2003, **86**(2):442-450.

33. Martin P, Villares R, Rodriguez-Mascarenhas S, Zaballos A, Leitges M, Kovac J, Sizing I, Rennert P, Marquez G, Martinez AC *et al*: **Control of T helper 2 cell function and allergic airway inflammation by PKCzeta**. *Proc Natl Acad Sci U S A* 2005, **102**(28):9866-9871.

34. Martin P, Duran A, Minguet S, Gaspar ML, Diaz-Meco MT, Rennert P, Leitges M, Moscat J: **Role of zeta PKC in B-cell signaling and function**. *Embo J* 2002, **21**(15):4049-4057.

35. Miyamoto A, Nakayama K, Imaki H, Hirose S, Jiang Y, Abe M, Tsukiyama T, Nagahama H, Ohno S, Hatakeyama S *et al*: **Increased proliferation of B cells and auto-immunity in mice lacking protein kinase Cdelta**. *Nature* 2002, **416**(6883):865-869.

36. Li MX, Jia M, Yang LX, Jiang H, Lanuza MA, Gonzalez CM, Nelson PG: **The role of the theta isoform of protein kinase C (PKC) in activity-dependent synapse elimination: evidence from the PKC theta knock-out mouse in vivo and in vitro**. *J Neurosci* 2004, **24**(15):3762-3769.

37. Sun Z, Arendt CW, Ellmeier W, Schaeffer EM, Sunshine MJ, Gandhi L, Annes J, Petrzilka D, Kupfer A, Schwartzberg PL *et al*: **PKC-theta is required for TCR-induced NF-kappaB activation in mature but not immature T lymphocytes**. *Nature* 2000, **404**(6776):402-407.

38. Pardo J, Buferne M, Martinez-Lorenzo MJ, Naval J, Schmitt-Verhulst AM, Boyer C, Anel A: **Differential implication of protein kinase C isoforms in cytotoxic T lymphocyte degranulation and TCR-induced Fas ligand expression**. *Int Immunol* 2003, **15**(12):1441-1450.

39. Cogram P, Hynes A, Dunlevy LP, Greene ND, Copp AJ: **Specific isoforms of protein kinase C are essential for prevention of folate-resistant neural tube defects by inositol**. *Hum Mol Genet* 2004, **13**(1):7-14.

40. Su TT, Guo B, Kawakami Y, Sommer K, Chae K, Humphries LA, Kato RM, Kang S, Patrone L, Wall R *et al*: **PKC-beta controls I kappa B kinase lipid raft recruitment and activation in response to BCR signaling**. *Nat Immunol* 2002, **3**(8):780-786.

41. Irvine EE, Vernon J, Giese KP: **AlphaCaMKII autophosphorylation contributes to rapid learning but is not necessary for memory**. *Nat Neurosci* 2005, **8**(4):411-412.

42. Bui JD, Calbo S, Hayden-Martinez K, Kane LP, Gardner P, Hedrick SM: **A role for CaMKII in T cell memory**. *Cell* 2000, **100**(4):457-467.

43. Zhuo M, Zhang W, Son H, Mansuy I, Sobel RA, Seidman J, Kandel ER: **A selective role of calcineurin aalpha in synaptic depotentiation in hippocampus**. *Proc Natl Acad Sci U S A* 1999, **96**(8):4650-4655.

44. Zhang BW, Zimmer G, Chen J, Ladd D, Li E, Alt FW, Wiederrecht G, Cryan J, O'Neill EA, Seidman CE *et al*: **T cell responses in calcineurin A alpha-deficient mice**. *J Exp Med* 1996, **183**(2):413-420.

45. Bueno OF, Brandt EB, Rothenberg ME, Molkentin JD: **Defective T cell development and function in calcineurin A beta -deficient mice**. *Proc Natl Acad Sci U S A* 2002, **99**(14):9398-9403.

46. Zeng H, Chattarji S, Barbarosie M, Rondi-Reig L, Philpot BD, Miyakawa T, Bear MF, Tonegawa S: **Forebrain-specific calcineurin knockout selectively impairs bidirectional synaptic plasticity and working/episodic-like memory**. *Cell* 2001, **107**(5):617-629.

47. Neilson JR, Winslow MM, Hur EM, Crabtree GR: **Calcineurin B1 is essential for positive but not negative selection during thymocyte development**. *Immunity* 2004, **20**(3):255-266.

48. Trama J, Go WY, Ho SN: **The osmoprotective function of the NFAT5 transcription factor in T cell development and activation**. *J Immunol* 2002, **169**(10):5477-5488.

49. Kaisho T, Takeda K, Tsujimura T, Kawai T, Nomura F, Terada N, Akira S: **IkappaB kinase alpha is essential for mature B cell development and function**. *J Exp Med* 2001, **193**(4):417-426.

50. Ren H, Schmalstieg A, van Oers NS, Gaynor RB: **I-kappa B kinases alpha and beta have distinct roles in regulating murine T cell function**. *J Immunol* 2002, **168**(8):3721-3731.

51. Azoitei N, Wirth T, Baumann B: **Activation of the IkappaB kinase complex is sufficient for neuronal differentiation of PC12 cells**. *J Neurochem* 2005, **93**(6):1487-1501.

52. Li ZW, Omori SA, Labuda T, Karin M, Rickert RC: **IKK beta is required for peripheral B cell survival and proliferation**. *J Immunol* 2003, **170**(9):4630-4637.

53. Kassed CA, Willing AE, Garbuzova-Davis S, Sanberg PR, Pennypacker KR: **Lack of NF-kappaB p50 exacerbates degeneration of hippocampal neurons after chemical exposure and impairs learning**. *Exp Neurol* 2002, **176**(2):277-288.

54. Hilliard BA, Mason N, Xu L, Sun J, Lamhamedi-Cherradi SE, Liou HC, Hunter C, Chen YH: **Critical roles of c-Rel in autoimmune inflammation and helper T cell differentiation**. *J Clin Invest* 2002, **110**(6):843-850.

55. Corn RA, Hunter C, Liou HC, Siebenlist U, Boothby MR: **Opposing roles for RelB and Bcl-3 in regulation of T-box expressed in T cells, GATA-3, and Th effector differentiation**. *J Immunol* 2005, **175**(4):2102-2110.

56. O'Riordan KJ, Huang IC, Pizzi M, Spano P, Boroni F, Egli R, Desai P, Fitch O, Malone L, Ahn HJ *et al*: **Regulation of nuclear factor kappaB in the hippocampus by group I metabotropic glutamate receptors**. *J Neurosci* 2006, **26**(18):4870-4879.

57. Feng B, Cheng S, Hsia CY, King LB, Monroe JG, Liou HC: **NF-kappaB inducible genes BCL-X and cyclin E promote immature B-cell proliferation and survival**. *Cell Immunol* 2004, **232**(1-2):9-20.

58. Meffert MK, Chang JM, Wiltgen BJ, Fanselow MS, Baltimore D: **NF-kappa B functions in synaptic signaling and behavior**. *Nat Neurosci* 2003, **6**(10):1072-1078.

59. Horwitz BH, Zelazowski P, Shen Y, Wolcott KM, Scott ML, Baltimore D, Snapper CM: **The p65 subunit of NF-kappa B is redundant with p50 during B cell proliferative responses, and is required for germline CH transcription and class switching to IgG3**. *J Immunol* 1999, **162**(4):1941-1946.

60. Brambilla R, Bracchi-Ricard V, Hu WH, Frydel B, Bramwell A, Karmally S, Green EJ, Bethea JR: **Inhibition of astroglial nuclear factor kappaB reduces inflammation and improves functional recovery after spinal cord injury**. *J Exp Med* 2005, **202**(1):145-156.

61. Chen CL, Singh N, Yull FE, Strayhorn D, Van Kaer L, Kerr LD: **Lymphocytes lacking I kappa B-alpha develop normally, but have selective defects in proliferation and function**. *J Immunol* 2000, **165**(10):5418-5427.

62. Memet S, Laouini D, Epinat JC, Whiteside ST, Goudeau B, Philpott D, Kayal S, Sansonetti PJ, Berche P, Kanellopoulos J *et al*: **IkappaBepsilon-deficient mice: reduction of one T cell precursor subspecies and enhanced Ig isotype switching and cytokine synthesis**. *J Immunol* 1999, **163**(11):5994-6005.

63. Reif K, Okkenhaug K, Sasaki T, Penninger JM, Vanhaesebroeck B, Cyster JG: **Cutting edge: differential roles for phosphoinositide 3-kinases, p110gamma and p110delta, in lymphocyte chemotaxis and homing**. *J Immunol* 2004, **173**(4):2236-2240.

64. Alonzi T, Middleton G, Wyatt S, Buchman V, Betz UA, Muller W, Musiani P, Poli V, Davies AM: **Role of STAT3 and PI 3-kinase/Akt in mediating the survival actions of cytokines on sensory neurons**. *Mol Cell Neurosci* 2001, **18**(3):270-282.

65. Donahue AC, Hess KL, Ng KL, Fruman DA: **Altered splenic B cell subset development in mice lacking phosphoinositide 3-kinase p85alpha**. *Int Immunol* 2004, **16**(12):1789-1798.

66. Deane JA, Trifilo MJ, Yballe CM, Choi S, Lane TE, Fruman DA: **Enhanced T cell proliferation in mice lacking the p85beta subunit of phosphoinositide 3-kinase**. *J Immunol* 2004, **172**(11):6615-6625.

67. Backman SA, Stambolic V, Suzuki A, Haight J, Elia A, Pretorius J, Tsao MS, Shannon P, Bolon B, Ivy GO *et al*: **Deletion of Pten in mouse brain causes seizures, ataxia and defects in soma size resembling Lhermitte-Duclos disease**. *Nat Genet* 2001, **29**(4):396-403.

68. Anzelon AN, Wu H, Rickert RC: **Pten inactivation alters peripheral B lymphocyte fate and reconstitutes CD19 function**. *Nat Immunol* 2003, **4**(3):287-294.

69. Lai WS, Xu B, Westphal KG, Paterlini M, Olivier B, Pavlidis P, Karayiorgou M, Gogos JA: **Akt1 deficiency affects neuronal morphology and predisposes to abnormalities in prefrontal cortex functioning**. *Proc Natl Acad Sci U S A* 2006, **103**(45):16906-16911.

70. Kane LP, Mollenauer MN, Weiss A: **A proline-rich motif in the C terminus of Akt contributes to its localization in the immunological synapse**. *J Immunol* 2004, **172**(9):5441-5449.

71. Li G, Anderson RE, Tomita H, Adler R, Liu X, Zack DJ, Rajala RV: **Nonredundant role of Akt2 for neuroprotection of rod photoreceptor cells from light-induced cell death**. *J Neurosci* 2007, **27**(1):203-211.

72. Tschopp O, Yang ZZ, Brodbeck D, Dummler BA, Hemmings-Mieszczak M, Watanabe T, Michaelis T, Frahm J, Hemmings BA: **Essential role of protein kinase B gamma (PKB gamma/Akt3) in postnatal brain development but not in glucose homeostasis**. *Development* 2005, **132**(13):2943-2954.

73. Dey N, Howell BW, De PK, Durden DL: **CSK negatively regulates nerve growth factor induced neural differentiation and augments AKT kinase activity**. *Exp Cell Res* 2005, **307**(1):1-14.

74. Schmedt C, Saijo K, Niidome T, Kuhn R, Aizawa S, Tarakhovsky A: **Csk controls antigen receptor-mediated development and selection of T-lineage cells**. *Nature* 1998, **394**(6696):901-904.

75. Lee BC, Avraham S, Imamoto A, Avraham HK: **Identification of the nonreceptor tyrosine kinase MATK/CHK as an essential regulator of immune cells using Matk/CHK-deficient mice**. *Blood* 2006, **108**(3):904-907.

76. Jones SB, Lu HY, Lu Q: **Abl tyrosine kinase promotes dendrogenesis by inducing actin cytoskeletal rearrangements in cooperation with Rho family small GTPases in hippocampal neurons**. *J Neurosci* 2004, **24**(39):8510-8521.

77. Zipfel PA, Grove M, Blackburn K, Fujimoto M, Tedder TF, Pendergast AM: **The c-Abl tyrosine kinase is regulated downstream of the B cell antigen receptor and interacts with CD19**. *J Immunol* 2000, **165**(12):6872-6879.

78. Moresco EM, Donaldson S, Williamson A, Koleske AJ: **Integrin-mediated dendrite branch maintenance requires Abelson (Abl) family kinases**. *J Neurosci* 2005, **25**(26):6105-6118.

79. Yang EJ, Yoon JH, Chung KC: **Bruton's tyrosine kinase phosphorylates cAMP-responsive element-binding protein at serine 133 during neuronal differentiation in immortalized hippocampal progenitor cells**. *J Biol Chem* 2004, **279**(3):1827-1837.

80. Kouro T, Nagata K, Takaki S, Nisitani S, Hirano M, Wahl MI, Witte ON, Karasuyama H, Takatsu K: **Bruton's tyrosine kinase is required for signaling the CD79b-mediated pro-B to pre-B cell transition**. *Int Immunol* 2001, **13**(4):485-493.

81. Schaeffer EM, Yap GS, Lewis CM, Czar MJ, McVicar DW, Cheever AW, Sher A, Schwartzberg PL: **Mutation of Tec family kinases alters T helper cell differentiation**. *Nat Immunol* 2001, **2**(12):1183-1188.

82. Garcon F, Bismuth G, Isnardon D, Olive D, Nunes JA: **Tec kinase migrates to the T cell-APC interface independently of its pleckstrin homology domain**. *J Immunol* 2004, **173**(2):770-775.

83. Sommers CL, Rabin RL, Grinberg A, Tsay HC, Farber J, Love PE: **A role for the Tec family tyrosine kinase Txk in T cell activation and thymocyte selection**. *J Exp Med* 1999, **190**(10):1427-1438.

84. Shen R, Ouyang YB, Qu CK, Alonso A, Sperzel L, Mustelin T, Kaplan MH, Feng GS: **Grap negatively regulates T-cell receptor-elicited lymphocyte proliferation and interleukin-2 induction**. *Mol Cell Biol* 2002, **22**(10):3230-3236.

85. Saxton TM, Cheng AM, Ong SH, Lu Y, Sakai R, Cross JC, Pawson T: **Gene dosage-dependent functions for phosphotyrosine-Grb2 signaling during mammalian tissue morphogenesis**. *Curr Biol* 2001, **11**(9):662-670.

86. Gong Q, Cheng AM, Akk AM, Alberola-Ila J, Gong G, Pawson T, Chan AC: **Disruption of T cell signaling networks and development by Grb2 haploid insufficiency**. *Nat Immunol* 2001, **2**(1):29-36.

87. Flemming A, Brummer T, Reth M, Jumaa H: **The adaptor protein SLP-65 acts as a tumor suppressor that limits pre-B cell expansion**. *Nat Immunol* 2003, **4**(1):38-43.

88. Aoki K, Nakamura T, Fujikawa K, Matsuda M: **Local phosphatidylinositol 3,4,5-trisphosphate accumulation recruits Vav2 and Vav3 to activate Rac1/Cdc42 and initiate neurite outgrowth in nerve growth factor-stimulated PC12 cells**. *Mol Biol Cell* 2005, **16**(5):2207-2217.

89. Salojin K, Zhang J, Cameron M, Gill B, Arreaza G, Ochi A, Delovitch TL: **Impaired plasma membrane targeting of Grb2-murine son of sevenless (mSOS) complex and differential activation of the Fyn-T cell receptor (TCR)-zeta-Cbl pathway mediate T cell hyporesponsiveness in autoimmune nonobese diabetic mice**. *J Exp Med* 1997, **186**(6):887-897.

90. Vogel KS, El-Afandi M, Parada LF: **Neurofibromin negatively regulates neurotrophin signaling through p21ras in embryonic sensory neurons**. *Mol Cell Neurosci* 2000, **15**(4):398-407.

91. Heumann R, Goemans C, Bartsch D, Lingenhohl K, Waldmeier PC, Hengerer B, Allegrini PR, Schellander K, Wagner EF, Arendt T *et al*: **Transgenic activation of Ras in neurons promotes hypertrophy and protects from lesion-induced degeneration**. *J Cell Biol* 2000, **151**(7):1537-1548.

92. Swan KA, Alberola-Ila J, Gross JA, Appleby MW, Forbush KA, Thomas JF, Perlmutter RM: **Involvement of p21ras distinguishes positive and negative selection in thymocytes**. *Embo J* 1995, **14**(2):276-285.

93. Ohno M, Frankland PW, Chen AP, Costa RM, Silva AJ: **Inducible, pharmacogenetic approaches to the study of learning and memory**. *Nat Neurosci* 2001, **4**(12):1238-1243.

94. Pritchard CA, Bolin L, Slattery R, Murray R, McMahon M: **Post-natal lethality and neurological and gastrointestinal defects in mice with targeted disruption of the A-Raf protein kinase gene**. *Curr Biol* 1996, **6**(5):614-617.

95. Chen AP, Ohno M, Giese KP, Kuhn R, Chen RL, Silva AJ: **Forebrain-specific knockout of B-raf kinase leads to deficits in hippocampal long-term potentiation, learning, and memory**. *J Neurosci Res* 2006, **83**(1):28-38.

96. Tsukamoto H, Irie A, Nishimura Y: **B-Raf contributes to sustained extracellular signal-regulated kinase activation associated with interleukin-2 production stimulated through the T cell receptor**. *J Biol Chem* 2004, **279**(46):48457-48465.

97. Tansey MG, Chu GC, Merlie JP: **ARIA/HRG regulates AChR epsilon subunit gene expression at the neuromuscular synapse via activation of phosphatidylinositol 3-kinase and Ras/MAPK pathway**. *J Cell Biol* 1996, **134**(2):465-476.

98. Owaki H, Varma R, Gillis B, Bruder JT, Rapp UR, Davis LS, Geppert TD: **Raf-1 is required for T cell IL2 production**. *Embo J* 1993, **12**(11):4367-4373.

99. Fleischmann A, Hvalby O, Jensen V, Strekalova T, Zacher C, Layer LE, Kvello A, Reschke M, Spanagel R, Sprengel R *et al*: **Impaired long-term memory and NR2A-type NMDA receptor-dependent synaptic plasticity in mice lacking c-Fos in the CNS**. *J Neurosci* 2003, **23**(27):9116-9122.

100. Koizumi T, Ochi Y, Kobayashi S, Nakanishi M, Tokuhisa T: **Deregulated expression of c-fos disturbs proliferative responses of B cells to sIg cross-linking**. *Cell Immunol* 1994, **155**(2):384-393.

101. Hiroi N, Marek GJ, Brown JR, Ye H, Saudou F, Vaidya VA, Duman RS, Greenberg ME, Nestler EJ: **Essential role of the fosB gene in molecular, cellular, and behavioral actions of chronic electroconvulsive seizures**. *J Neurosci* 1998, **18**(17):6952-6962.

102. Carrozza ML, Jacobs H, Acton D, Verma I, Berns A: **Overexpression of the FosB2 gene in thymocytes causes aberrant development of T cells and thymic epithelial cells**. *Oncogene* 1997, **14**(9):1083-1091.

103. Palmada M, Kanwal S, Rutkoski NJ, Gustafson-Brown C, Johnson RS, Wisdom R, Carter BD: **c-jun is essential for sympathetic neuronal death induced by NGF withdrawal but not by p75 activation**. *J Cell Biol* 2002, **158**(3):453-461.

104. Petrak D, Memon SA, Birrer MJ, Ashwell JD, Zacharchuk CM: **Dominant negative mutant of c-Jun inhibits NF-AT transcriptional activity and prevents IL-2 gene transcription**. *J Immunol* 1994, **153**(5):2046-2051.

105. Hartenstein B, Teurich S, Hess J, Schenkel J, Schorpp-Kistner M, Angel P: **Th2 cell-specific cytokine expression and allergen-induced airway inflammation depend on JunB**. *Embo J* 2002, **21**(23):6321-6329.

106. Meixner A, Karreth F, Kenner L, Wagner EF: **JunD regulates lymphocyte proliferation and T helper cell cytokine expression**. *Embo J* 2004, **23**(6):1325-1335.

107. Sato S, Sanjo H, Takeda K, Ninomiya-Tsuji J, Yamamoto M, Kawai T, Matsumoto K, Takeuchi O, Akira S: **Essential function for the kinase TAK1 in innate and adaptive immune responses**. *Nat Immunol* 2005, **6**(11):1087-1095.

108. Chi H, Sarkisian MR, Rakic P, Flavell RA: **Loss of mitogen-activated protein kinase kinase kinase 4 (MEKK4) results in enhanced apoptosis and defective neural tube development**. *Proc Natl Acad Sci U S A* 2005, **102**(10):3846-3851.

109. Chi H, Lu B, Takekawa M, Davis RJ, Flavell RA: **GADD45beta/GADD45gamma and MEKK4 comprise a genetic pathway mediating STAT4-independent IFNgamma production in T cells**. *Embo J* 2004, **23**(7):1576-1586.

110. Waetzig V, Herdegen T: **A single c-Jun N-terminal kinase isoform (JNK3-p54) is an effector in both neuronal differentiation and cell death**. *J Biol Chem* 2003, **278**(1):567-572.

111. Amura CR, Marek L, Winn RA, Heasley LE: **Inhibited neurogenesis in JNK1-deficient embryonic stem cells**. *Mol Cell Biol* 2005, **25**(24):10791-10802.

112. Dong C, Yang DD, Wysk M, Whitmarsh AJ, Davis RJ, Flavell RA: **Defective T cell differentiation in the absence of Jnk1**. *Science* 1998, **282**(5396):2092-2095.

113. Chen JT, Lu DH, Chia CP, Ruan DY, Sabapathy K, Xiao ZC: **Impaired long-term potentiation in c-Jun N-terminal kinase 2-deficient mice**. *J Neurochem* 2005, **93**(2):463-473.

114. Yang DD, Conze D, Whitmarsh AJ, Barrett T, Davis RJ, Rincon M, Flavell RA: **Differentiation of CD4+ T cells to Th1 cells requires MAP kinase JNK2**. *Immunity* 1998, **9**(4):575-585.

115. Fischer AM, Katayama CD, Pages G, Pouyssegur J, Hedrick SM: **The role of erk1 and erk2 in multiple stages of T cell development**. *Immunity* 2005, **23**(4):431-443.

116. Mazzucchelli C, Vantaggiato C, Ciamei A, Fasano S, Pakhotin P, Krezel W, Welzl H, Wolfer DP, Pages G, Valverde O *et al*: **Knockout of ERK1 MAP kinase enhances synaptic plasticity in the striatum and facilitates striatal-mediated learning and memory**. *Neuron* 2002, **34**(5):807-820.

117. Pages G, Guerin S, Grall D, Bonino F, Smith A, Anjuere F, Auberger P, Pouyssegur J: **Defective thymocyte maturation in p44 MAP kinase (Erk 1) knockout mice**. *Science* 1999, **286**(5443):1374-1377.

118. Xia Z, Dickens M, Raingeaud J, Davis RJ, Greenberg ME: **Opposing effects of ERK and JNK-p38 MAP kinases on apoptosis**. *Science* 1995, **270**(5240):1326-1331.

119. Tanaka N, Kamanaka M, Enslen H, Dong C, Wysk M, Davis RJ, Flavell RA: **Differential involvement of p38 mitogen-activated protein kinase kinases MKK3 and MKK6 in T-cell apoptosis**. *EMBO Rep* 2002, **3**(8):785-791.

120. Alberola-Ila J, Hogquist KA, Swan KA, Bevan MJ, Perlmutter RM: **Positive and negative selection invoke distinct signaling pathways**. *J Exp Med* 1996, **184**(1):9-18.

121. Dong C, Yang DD, Tournier C, Whitmarsh AJ, Xu J, Davis RJ, Flavell RA: **JNK is required for effector T-cell function but not for T-cell activation**. *Nature* 2000, **405**(6782):91-94.

122. Nishina H, Fischer KD, Radvanyi L, Shahinian A, Hakem R, Rubie EA, Bernstein A, Mak TW, Woodgett JR, Penninger JM: **Stress-signalling kinase Sek1 protects thymocytes from apoptosis mediated by CD95 and CD3**. *Nature* 1997, **385**(6614):350-353.

123. Nishina H, Bachmann M, Oliveira-dos-Santos AJ, Kozieradzki I, Fischer KD, Odermatt B, Wakeham A, Shahinian A, Takimoto H, Bernstein A *et al*: **Impaired CD28-mediated interleukin 2 production and proliferation in stress kinase SAPK/ERK1 kinase (SEK1)/mitogen-activated protein kinase kinase 4 (MKK4)-deficient T lymphocytes**. *J Exp Med* 1997, **186**(6):941-953.

124. Hayashi K, Ohshima T, Mikoshiba K: **Pak1 is involved in dendrite initiation as a downstream effector of Rac1 in cortical neurons**. *Mol Cell Neurosci* 2002, **20**(4):579-594.

125. Gomez M, Kioussis D, Cantrell DA: **The GTPase Rac-1 controls cell fate in the thymus by diverting thymocytes from positive to negative selection**. *Immunity* 2001, **15**(5):703-713.

126. Walmsley MJ, Ooi SK, Reynolds LF, Smith SH, Ruf S, Mathiot A, Vanes L, Williams DA, Cancro MP, Tybulewicz VL: **Critical roles for Rac1 and Rac2 GTPases in B cell development and signaling**. *Science* 2003, **302**(5644):459-462.

127. Orioli D, Colaluca IN, Stefanini M, Riva S, Dotti CG, Peverali FA: **Rac3-induced neuritogenesis requires binding to Neurabin I**. *Mol Biol Cell* 2006, **17**(5):2391-2400.

128. Tskvitaria-Fuller I, Seth A, Mistry N, Gu H, Rosen MK, Wulfing C: **Specific patterns of Cdc42 activity are related to distinct elements of T cell polarization**. *J Immunol* 2006, **177**(3):1708-1720.

129. Duchniewicz M, Zemojtel T, Kolanczyk M, Grossmann S, Scheele JS, Zwartkruis FJ: **Rap1A-deficient T and B cells show impaired integrin-mediated cell adhesion**. *Mol Cell Biol* 2006, **26**(2):643-653.

130. Wang Y, Su B, Xia Z: **Brain-derived neurotrophic factor activates ERK5 in cortical neurons via a Rap1-MEKK2 signaling cascade**. *J Biol Chem* 2006, **281**(47):35965-35974.

131. Fischer KD, Zmuldzinas A, Gardner S, Barbacid M, Bernstein A, Guidos C: **Defective T-cell receptor signalling and positive selection of Vav-deficient CD4+ CD8+ thymocytes**. *Nature* 1995, **374**(6521):474-477.

132. Doody GM, Bell SE, Vigorito E, Clayton E, McAdam S, Tooze R, Fernandez C, Lee IJ, Turner M: **Signal transduction through Vav-2 participates in humoral immune responses and B cell maturation**. *Nat Immunol* 2001, **2**(6):542-547.

133. McFarland KN, Wilkes SR, Koss SE, Ravichandran KS, Mandell JW: **Neural-specific inactivation of ShcA results in increased embryonic neural progenitor apoptosis and microencephaly**. *J Neurosci* 2006, **26**(30):7885-7897.

134. Zhang L, Camerini V, Bender TP, Ravichandran KS: **A nonredundant role for the adapter protein Shc in thymic T cell development**. *Nat Immunol* 2002, **3**(8):749-755.

135. Sakai R, Henderson JT, O'Bryan JP, Elia AJ, Saxton TM, Pawson T: **The mammalian ShcB and ShcC phosphotyrosine docking proteins function in the maturation of sensory and sympathetic neurons**. *Neuron* 2000, **28**(3):819-833.

136. Miyamoto Y, Chen L, Sato M, Sokabe M, Nabeshima T, Pawson T, Sakai R, Mori N: **Hippocampal synaptic modulation by the phosphotyrosine adapter protein ShcC/N-Shc via interaction with the NMDA receptor**. *J Neurosci* 2005, **25**(7):1826-1835.

137. Korhonen JM, Said FA, Wong AJ, Kaplan DR: **Gab1 mediates neurite outgrowth, DNA synthesis, and survival in PC12 cells**. *J Biol Chem* 1999, **274**(52):37307-37314.

138. Itoh S, Itoh M, Nishida K, Yamasaki S, Yoshida Y, Narimatsu M, Park SJ, Hibi M, Ishihara K, Hirano T: **Adapter molecule Grb2-associated binder 1 is specifically expressed in marginal zone B cells and negatively regulates thymus-independent antigen-2 responses**. *J Immunol* 2002, **168**(10):5110-5116.

139. Yamasaki S, Nishida K, Hibi M, Sakuma M, Shiina R, Takeuchi A, Ohnishi H, Hirano T, Saito T: **Docking protein Gab2 is phosphorylated by ZAP-70 and negatively regulates T cell receptor signaling by recruitment of inhibitory molecules**. *J Biol Chem* 2001, **276**(48):45175-45183.
